# Comparative Genomics, Evolution, and Drought-Induced Expression of Dehydrin Genes in Model *Brachypodium* Grasses

**DOI:** 10.3390/plants10122664

**Published:** 2021-12-03

**Authors:** Maria Angeles Decena, Sergio Gálvez-Rojas, Federico Agostini, Ruben Sancho, Bruno Contreras-Moreira, David L. Des Marais, Pilar Hernandez, Pilar Catalán

**Affiliations:** 1 Escuela Politécnica Superior de Huesca, Universidad de Zaragoza, Ctra. Cuarte km 1, 22071 Huesca, Spain; mdecena@unizar.es (M.A.D.); ruben.sancho.cohen@gmail.com (R.S.); 2 ETSI Informática, Universidad de Málaga, Blvr Louis Pasteur 35, 29071 Málaga, Spain; galvez@uma.es (S.G.-R.); fagostini@conicet.gov.ar (F.A.); 3 Instituto de Botánica del Nordeste, UNNE-CONICET, Corrientes W3402, Argentina; 4 Grupo de Bioquímica, Biofísica y Biología Computacional (BIFI, UNIZAR), Unidad Asociada al CSIC, 50018 Zaragoza, Spain; bcontreras@eead.csic.es; 5 Estación Experimental de Aula Dei-Consejo Superior de Investigaciones Científicas, Av. Montañana 1005, 50059 Zaragoza, Spain; 6 Civil and Environmental Engineering Department, Faculty of Environmental and Life Science, Massachusetts Institute of Technology, 15 Vassar Street, Cambridge, MA 02139, USA; dldesmar@mit.edu; 7 Instituto de Agricultura Sostenible, IAS-CSIC, Menendez Pidal Ave, 14004 Córdoba, Spain; 8 Departamento de Ciencias Agrarias y del Medio Natural, Tomsk State University, 36 Lenin Ave, 634050 Tomsk, Russia

**Keywords:** *Bdhn* genes, *cis*-regulatory elements, comparative genomics, dehydrin structure, dehydrin-gene expression, duplicated genes, drought-related traits, drought-tolerant ecotypes, phylogenetics

## Abstract

Dehydration proteins (dehydrins, DHNs) confer tolerance to water-stress deficit in plants. We performed a comparative genomics and evolutionary study of DHN genes in four model *Brachypodium* grass species. Due to limited knowledge on dehydrin expression under water deprivation stress in *Brachypodium,* we also performed a drought-induced gene expression analysis in 32 ecotypes of the genus’ flagship species *B. distachyon* showing different hydric requirements. Genomic sequence analysis detected 10 types of dehydrin genes (*Bdhn*) across the *Brachypodium* species. Domain and conserved motif contents of peptides encoded by *Bdhn* genes revealed eight protein architectures. *Bdhn* genes were spread across several chromosomes. Selection analysis indicated that all the *Bdhn* genes were constrained by purifying selection. Three upstream *cis*-regulatory motifs (BES1, MYB124, ZAT) were detected in several *Bdhn* genes. Gene expression analysis demonstrated that only four *Bdhn*1-*Bdhn*2, *Bdhn*3, and *Bdhn*7 genes, orthologs of wheat, barley, rice, sorghum, and maize genes, were expressed in mature leaves of *B. distachyon* and that all of them were more highly expressed in plants under drought conditions. *Brachypodium* dehydrin expression was significantly correlated with drought-response phenotypic traits (plant biomass, leaf carbon and proline contents and water use efficiency increases, and leaf water and nitrogen content decreases) being more pronounced in drought-tolerant ecotypes. Our results indicate that dehydrin type and regulation could be a key factor determining the acquisition of water-stress tolerance in grasses.

## 1. Introduction

Water deprivation is one of the main abiotic stresses that affect plant development and fitness [1,2]. Water deficit stress in plants is mostly caused by low soil water content but also by other abiotic stresses such as salinity and extreme temperature [3,4]—which sometimes interact [2]—affecting the survival of plants in a wide range of environmental conditions [4,5]. Plant species adapted to dry environments have developed mechanisms to protect their cells from water stress deficit. Among the several physiological and genomic regulatory mechanisms triggered by water limitation in plants, there is an almost universal response in the upregulation of dehydrins [1,4,6]. Dehydrins (DHN) belong to group 2 LEA (Late-Embryogenesis-Abundant) proteins [7], and are intrinsically disordered hydrophilic proteins that acquire structure when bound to ligands, such as membranes, acting as chaperones that impede the aggregation or inactivation of other proteins under desiccation to maintain the biological activity of the cell [8,9]. They show a high hydration capacity and can also bind large quantities of cations, retaining water in the drying cells and preventing ionic unbalance and protein denaturation. DHNs are also called RAB proteins because they are usually responsive to abscisic acid [9]. DHNs accumulate in all vegetative tissues under water stress, though with different specificities [4], as well as during seed development [8].

**Figure 1:**
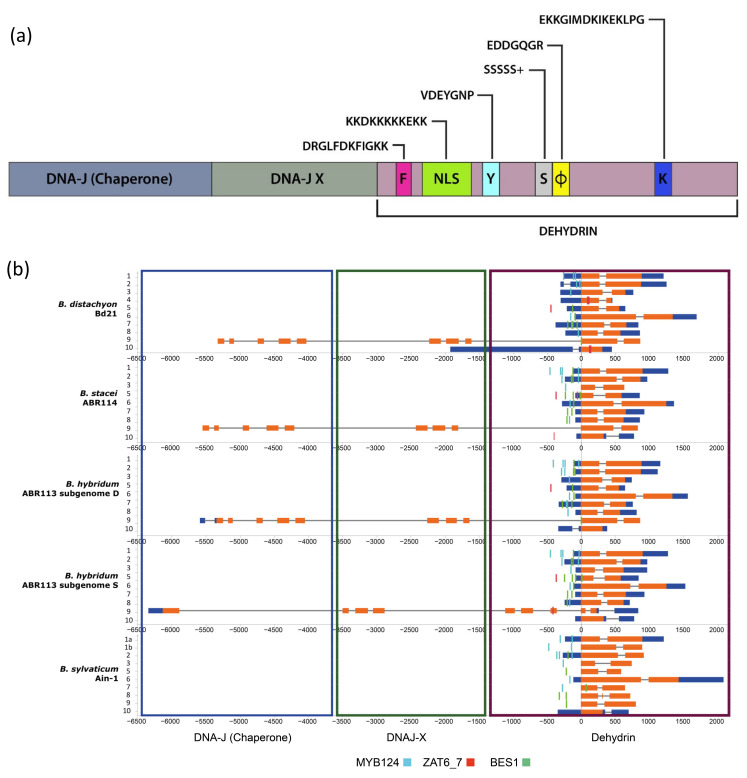

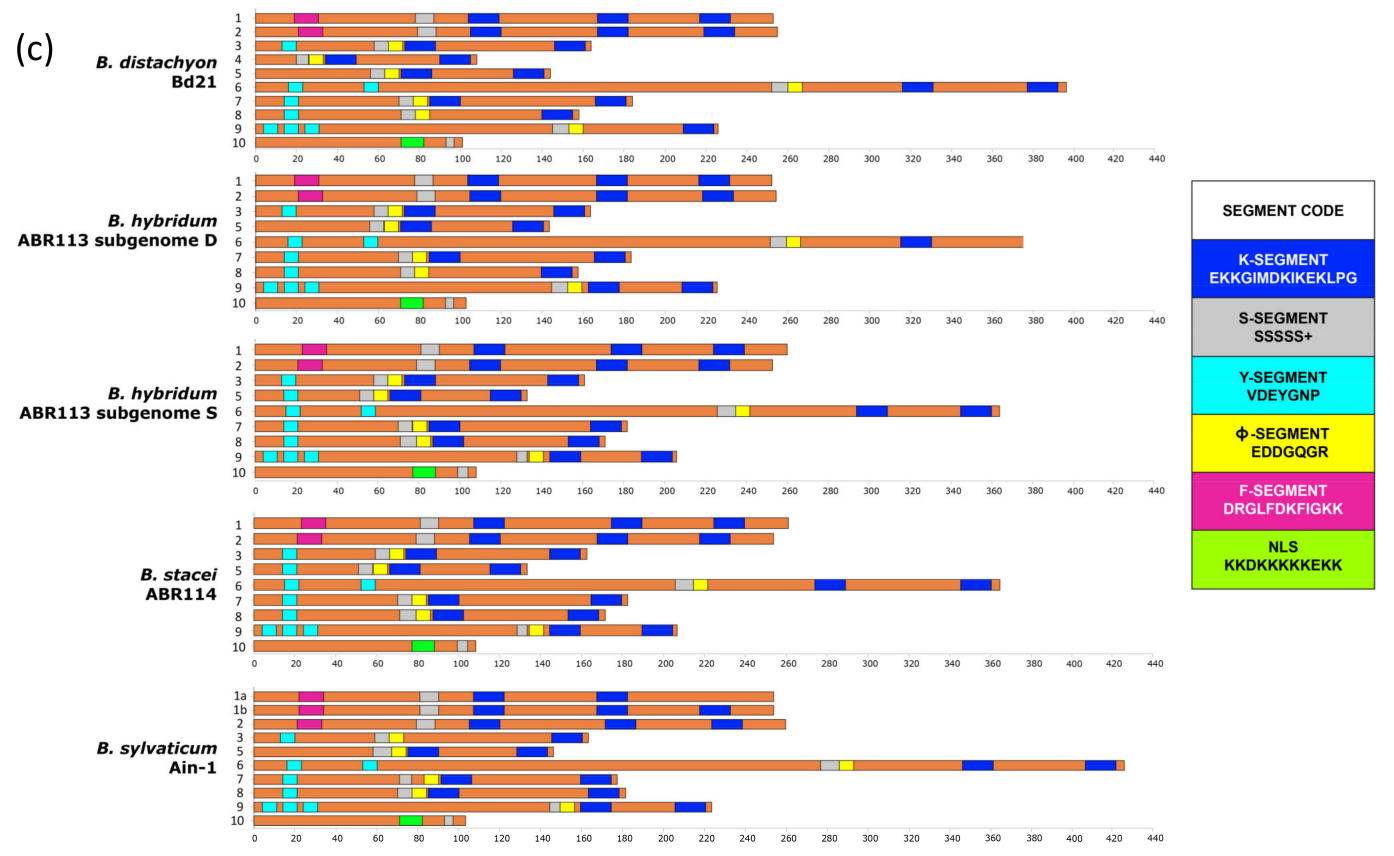
Schematic structure of a typical grass DHN gene showing the positions of the main domains and coding segments (**a**). Gene structure of *Brachypodium distachyon* Bd21, *B. stacei* ABR114, *B. hybridum* ABR113 (D and S subgenomes), and *B. sylvaticum* Ain-1 dehydrins showing: (**b**) the three detected DNA J (purple rectangle), DNA J-X (light green rectangle) and dehydrin (dark green rectangle) domains, and their CDSs (orange bars), introns (grey lines), and 5′ and 3′ UTRs (blue bars); (**c**) the conserved motifs of their CDSs with their respective K (dark blue), S (grey), Y (light blue), ϕ (yellow), F (pink), and NLS (light green) segments. *Brachypodium* dehydrin gene codes (*Bdhn1–Bdhn10*) correspond to those indicated in Table 1. *Cis*-regulatory elements BES1, MYB124, and ZAT are mapped in the promoters of each gene (see color codes in the chart; the figure could be also visualized in http://zeta.uma.es/public/journal/brachy/DHN_Brachy_4_varieties.html, 29 November 2021).

The dehydrin protein family is characterized by a modular sequence domain (Dehydrin) that contains three conserved segments (Y, S, K; [8,10]; Figure 1a). The dehydrin identifier K-segment motif is present in all plant DHNs except for HIRD11 (see Section 2). Perdiguero et al. demonstrated the induction of dehydrin genes lacking the K-segment under drought stress in *Pinus* [11]. The S-segment is located between the Y and K segments and, when phosphorylated, transfers dehydrins and other nuclear localization signal (NLS) binding proteins from the cytosol to the nucleus [10,12]. The Y-segment motif is located towards the N-terminus of the protein coding region. The Y, S, and K segments are interspersed by other less conserved segments, named interpatter or ϕ-segments that mostly contain small, polar, and charged amino acids [13]. Recent studies have described another conserved F-segment motif in some seed plant DHNs [11,14]. This segment, when present, has a unique copy and is located between the N-terminus of the protein coding region and the S-segment. An additional NLS-segment motif was found in some dehydrin families of maize [12]. Different combinations of conserved motifs have been used to classify the dehydrin domain into major architectures, with five of them (K_n_, SK_n_, K_n_S, Y_n_K_n_, Y_n_SK_n_) being common across angiosperms [10]. The dehydrin domain is occasionally fused with upstream DNAJ-X and DNA-J containing gene domains in some DHN genes [10]. DNA-J is a heat shock protein (Hsp40) that prevents the aggregation of unfolded proteins and functions as a chaperone folding protein when associated with Hsp70 under water-stress [10,15] (Figure 1a). Evolutionary studies of spermatophyte dehydrins identified Kn or SKn as the oldest ancestral architectures whereas those containing the Y segment arose only in the angiosperms [10,16]. Two main architectural changes apparently occurred after the ancestral whole genome duplication events of the angiosperms, the Y3SK2-Y3K1 and Y1SK2-K6 dehydrins [10]. Whereas K6 developed a novel function in cold protection (but see [16] for the important role of Kn dehydrins in drought protection), Y3K1 showed signatures of pseudogenization in some families (e.g., the Brassicaceae) [10]. At the infrageneric level, the dehydrin phylogeny of 11 *Oryza* species showed the splits of four high-to-weakly supported domain clades and a lack of dehydrin-specific subclades [9].

DHNs have been extensively studied in grasses due to their key role as agents conferring water-stress tolerance in cereal and forage crops [7,9,17,18]. Expression of dehydrins under drought stress has been positively associated with plant biomass and grain yield [19]. Several in vitro studies have demonstrated that DHN expression enhances plant stress tolerance [20,21]. Dehydrins maintain the osmotic balance of cells and their chlorophyll contents, bind metals to scavenge ROS, and bind to DNA and phospholipids [20,22]. Despite these advances, some of the biological functions of DHNs have not been fully established yet [10]. Few dehydrins have been characterized biochemically, like those involved in enzyme and membrane bioprotection in *Triticum* [23] and *Zea* [24] and in protection against ROS in *Arabidopsis* and *Brassica* [25,26]. Their protective activities have been experimentally demonstrated in some cases using circular dichroism (e.g., *Zea mays* DHN1 binding to lipid vesicles, [27]; *Hordeum vulgare* Dhn5 protection of lactate deshydrogenase under drought and cold conditions, [28]).

*Brachypodium* is a model system for grasses due to its intermediate evolutionary position between the temperate cereals (Triticeae) and the tropical biofuel (*Miscanthus*, *Paspalum*) crops [29]. The three annual species of the genus have been selected as a model complex for polyploidy (diploids *B. distachyon* and *B. stacei* and derived allotetraploid *B. hybridum*; [30]) and one of its perennial species has been selected as a model species for perenniality (diploid *B. sylvaticum*; [31]). Transgenic plants of its flagship species *B. distachyon* have been analyzed to identify candidate genes that enhance drought stress tolerance in plants [32,33,34] though none of them specifically addressed dehydrins. Inspection of an early version of the *B. distachyon* reference genome Bd21 detected 36 LEA2 encoding genes but did not characterize the dehydrin genes [35]. The dehydrin gene content, structure, evolution, and expression in response to drought among species and accessions of *Brachypodium* has not been investigated yet.

Given the significance of DHNs in water stress response of grasses, we analyzed the members of the dehydrin gene family in the four *Brachypodium* model species and in 54 *B. distachyon* ecotypes showing different hydric requirements and drought tolerances using in silico analysis of genome annotations. Due to the lack of studies on dehydrin expression under water deprivation stress in *Brachypodium* we also performed DHN expression analysis in 32 *B. distachyon* ecotypes under different drought and watered conditions. The aims of our study were: (i) to identify and characterize the *Brachypodium* dehydrin genes (*Bdhn*) and the structure and biochemical properties of the encoded proteins, comparing them with those of the closely related cereal crops (*Hordeum*, *Oryza*, *Sorghum*, *Triticum-Aegilops*, *Zea*), and to evaluate the presence of enriched sequence motifs that may have potential regulatory effects (ii) to analyze the syntenic distributions and origins of the *Bdhn* genes, identify gene duplication events, and test the functionality of paralogs, (iii) to investigate their expression profiles under control vs. dry conditions and compare them to those of closely related cereals, and (iv) to correlate the dehydrin gene expressions with the phenotypic and environmental traits of the plants and test their potential phylogenetic signal.

## 2. Results

### 2.1. Dehydrin Genes of Brachypodium Species and Outgroup Grasses

Genome searches in Phytozome and Ensembl Plants retrieved 47 dehydrin gene sequences collected from the reference genomes of the four sequenced *Brachypodium* species [*B. distachyon* Bd21 2n = 2x = 10, x = 5 (10); *B. sylvaticum* Ain-1 2n = 2x = 18, x = 9 (10); *B. stacei* ABR114 2n = 2x = 20, x = 10 (9); *B. hybridum* ABR113 2n = 4x = 30, x = 5 + 10 (18; 9 from its *B. distachyon*-type D subgenome and 9 from its *B. stacei*-type S subgenome)] (Table 1; Figure 1b). A total of 54 orthologous DHN sequences were retrieved from the reference genomes of six outgroup grass species [*Aegilops tauschii* 2n = 2x = 14, x = 7 (9); *Hordeum vulgare* 2n = 2x = 14, x = 7 (8); *Zea mays* 2n = 2x = 20, x = 10 (7); *Oryza sativa* 2n = 2x = 24, x = 12 (6); *Sorghum bicolor* 2n = 2x = 20, x = 10 (5); *Triticum aestivum* 2n = 6x = 42, x = 7 (19); Supplementary Table S1]. A new nomenclature was created for the *Brachypodium* dehydrin genes (*Bdhn*1 to *Bdhn*10; Table 1; Figure 1b,c). In several instances we used the same numbers as those of the orthologous *Hordeum vulgare* DHN genes (*Bdhn* 6-7, and *Bdhn* 9-10) (ENA database: AF043086, AF043092; AF043086 and Genbank database: AY681974) and the orthologous *Oryza sativa* DHN genes (*Bdhn*1-2 and *Bdhn*8) (RAP database: Os02g0669100, Os11g0454300). *Bdhn*3 was numbered according to prior annotation in *Brachypodium* (GeneBank: XM_010229280), whereas the remaining *Bdhn* genes were numbered consecutively as *Bdhn*4 and *Bdhn*5. The *Bdhn* genes were also classified according to Panther protein gene families (PTH33346 and PTH34941) and subfamilies (ERD14, XERO1, SF14, SF19, SF23, HIRD11; Table 1). All *Bdhn*10 genes found in the studied species of *Brachypodium* lacked the K-segment (Figure 1c). However, those dehydrins showed an extraordinary sequence identity with typical DHNs, including those with a modified K-segment, like DHN-13 from *H. vulgare* [36]. The absence of the K-segment has been also reported in one dehydrin of pine species [11] and four dehydrins of rice species (OnDHN6, OrDHN7, OlDHN3, OlDHN6; [9]). These *Brachypodium Bdhn*10 dehydrins, with architecture K*(NLS)S, belong to HIRD11 proteins and show orthology with DHN genes from *H. vulgare*, *O. sativa*, *S. bicolor,* and *Z. mays* (Supplementary Table S1).

The *Brachypodium* dehydrins showed different lengths, ranging from 86.3 (*Bdhn*10) to 323 amino acid residues (*Bdhn*6), molecular weights from 9762.90 to 30945.54 kDa, and isoelectric points from 4.41 (*Bdhn*2) to 7.6 (*Bdhn*7) (9.4 (*Bdhn*4) in *B. distachyon*) (Supplementary Table S2). All dehydrins presented a negative GRAVY value, indicating that they are hydrophilic proteins. All *Bdhn* genes except *Bdhn*5 were structurally conserved across the four *Brachypodium* species (Figure 1b,c). *Bdhn*4 was only present in *B. distachyon*, while *Bdhn*1 was duplicated in *B. sylvaticum* (*Bdhn*1a, *Bdhn*1b) (Table 1; Figure 1b,c). All *Bdhn* genes but *Bdhn*9 showed a single dehydrin domain. *Bdhn*9 encoded a protein of 508 aa with DHN–DNAJ-X–DNA-J domains in all species except in *B. sylvaticum,* which showed a gene consisting only of the DHN domain (222 aa) (Supplementary Table S2; Figure 1b). Eight gene architectures were found along the *Bdhn*1–*Bdhn*10 genes (FSK_2_, FSK_3_, SϕK_2_, YSϕK_2_, YSϕK, Y_3_SϕK, Y_3_SϕK_2_, NLS-K*S), with YSϕK_2_ being the most common architecture present in four genes (*Bdhn*3, *Bdhn*6, *Bdhn*7, *Bdhn*8; Table 1, Figure 1c). *Bdhn* 3D modeling indicated that the disordered *Brachypodium* dehydrins lacked tertiary structure whereas their secondary structure consisted of different numbers of α-helices (1–4) and β-sheets in some inferred proteins (Supplementary Figure S1).

Orthology analysis indicates that *Bdhn* genes were also present in the other surveyed grasses. In total, 6 out of 10 *Bdhn* genes were found in *A. tauschii; Bdhn*1-2 copies were also present in *H. vulgare* and *T. aestivum, Bdhn*3 in *T. aestivum, Bdhn*4-5 in *H. vulgare, Bdhn*7-8 in *T. aestivum, Bdhn*9 in *O. sativa* and *S. bicolor,* and *Bdhn*10 in *H. vulgare, O. sativa, S. bicolor,* and *Z. mays* (Supplementary Table S1). By contrast, five DHN genes of *O. sativa* (Os01g0702500, Os11g0453900, Os01g0624400, Os01g0225600, Os03g0655400), four of *H. vulgare* (HORVU6Hr1G083960, HORVU6Hr1G011050, HORVU5Hr1G103460, HORVU3Hr1G089300), and three of *Z. mays* (Zm00001d017547, Zm00001d043730, Zm00001d013647) had no orthologous sequences in *Brachypodium*. Pairwise amino acid sequence similarities indicated that *Bdhn*4 and *Bdhn*5 were the most similar proteins, followed by *Bdhn*1 and *Bdhn*2. *Bdhn*10 was the most dissimilar dehydrin. Dehydrins with YSϕK_2_ structure were in general similar to each other both in *Brachypodium* and in the other grasses.

### 2.2. Cis-Regulatory Elements of Bdhn Genes

We performed de novo discovery analysis of *cis*-regulatory elements (CREs) of *Bdhn* genes, searching for binding sites of transcription factors (TF) that accumulate around the transcriptional start site and that may control gene expression (Supplementary Figure S2). The analysis consistently identified three clusters, BES1/BZR1, MYB124, and ZAT binding sites (Table 2; Supplementary Figure S2a) that are related with different drought-response signaling pathways. BES1/BZR1 and MYB124 motifs were present in all studied promoters, though MYB124 was predominant in the promoters of *Bdhn*1 and *Bdhn*2 and BES1/BZR1in those of *Bdhn*7 genes and more abundantly in those of the aridic *B. stacei* and *B. hybridum* species. By contrast, ZAT was only found in the promoters of *Bdhn*4, *Bdhn*5, and *Bdhn*10 genes in annual species (Table 2; Supplementary Figure S2a).

### 2.3. The Brachypodium Dehydrin Tree

**Figure 2:**
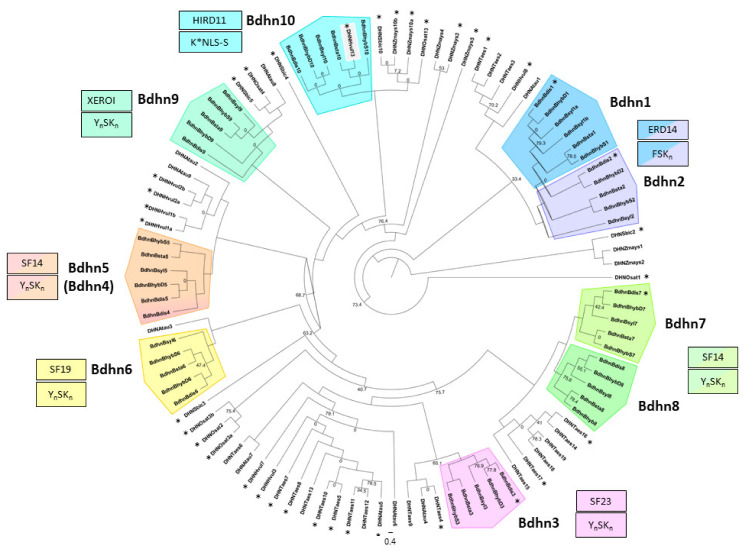
Maximum likelihood *Brachypodium Bdhn* tree. Unrooted IQtree cladogram showing the relationships among the dehydrin *Bdhn* gene clades and orthologous grass sequences (Atau: *Aegilops tauchii*; Hvul: *Hordeum vulgare*; Osat: *Oryza sativa*; Sbic: *Sorghum bicolor*; Taes: *Triticum aestivum*; Zmays: *Zea mays*) and among *Brachypodium* species and genomes within each clade. *Bdhn* clades are identified by colors, *Bdhn* codes, gene architecture, and Panther subfamily codes (see Table 1). Duplicated *Bdhn* genes form sister clades or fall within the same clade. Ultrafast bootstrap support (<80%) is shown on branches; the remaining branches are fully supported. Asterisks indicate dehydrin genes differentially expressed under drought vs. control conditions (see text and Supplementary Table S10). Scale bar: number of mutations per site.

To explore the evolutionary relationship among dehydrin genes, we constructed a ML *Brachypodium* dehydrin gene tree from 47 *Bdhn* protein coding regions present in the four studied *Brachypodium* species and in six outgroup grasses (Figure 2). All internal branches but three and all the dehydrin-type clades showed strong to relatively high bootstrap support values (BS ≥ 70%). The most divergent split separated the duplicated *Bdhn*1-*Bdhn*2 (ERD14) clade from the rest, followed by the isolated *Bdhn*10 (HIRD11) clade. Within the remaining group of Y_n_SK_n_ dehydrin structural genes, there was a divergence of the *Bdhn*9 (XEROI) clade, followed by subsequent divergences of the Y_n_SK_n_ *Bdhn*4-*Bdhn*5, *Bdhn*6, *Bdhn*3*,* and *Bdhn*7/*Bdhn*8 clades (Figure 2). All *Bdhn* clades were monophyletic except the paraphyletic *Bdhn*9 and *Bdhn*10 clades, which included orthologous sequences from closely related outgroups. Intra-clade branches were overall well-supported except for the poorly informative *Bdhn*10 clade.

### 2.4. Chromosome Distributions and Selection Analysis of Duplicated Bdhn Genes

**Figure 3:**
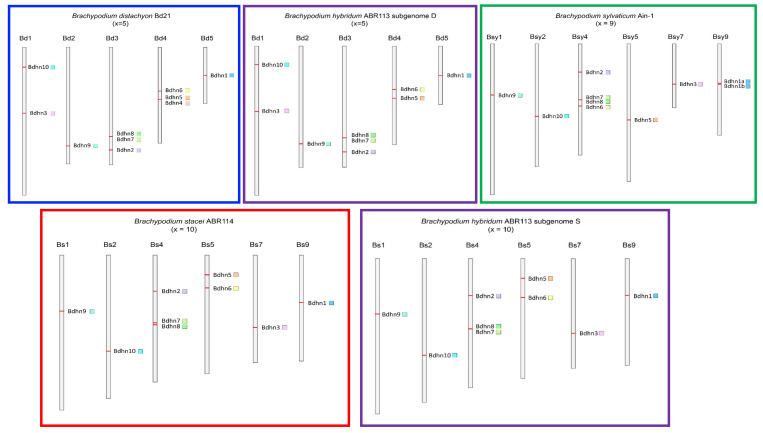
Chromosomal location of *Bdhn* genes in the studied *Brachypodium* species. *B. distachyon* (blue rectangle), *B. stacei* (red)*, B. hybridum* subgenomes S and D (purple), and *B. sylvaticum* (green). *Bdhn* genes are mapped on the chromosome with their respective color flags (see Figure 2).

We analyzed the physical distributions of *Bdhn* genes on the chromosomes of the studied species to detect the potential occurrence of tandem or segmental duplication. We also performed selection tests on the coding regions of the *Bdhn* genes to explore the potential loss of selective constrains on them. Dehydrin genes were distributed among the five chromosomes of *B. distachyon Bd21* and the D subgenome of *B. hybridum ABR113* (Bd), in 6 out of 10 chromosomes of *B. stacei ABR114* and the S subgenome of *B. hybridum ABR113* (Bs), and in 6 out of 9 chromosomes of *B. sylvaticum Ain-1* (Bsy) (Figure 3, Supplementary Table S3). We detected four tandem duplications and two segmental duplications of *Bdhn* genes. In *B. distachyon* and *B. hybridum* D subgenome our analysis detected tandem duplications of *Bdhn*7-*Bdhn*8 in Bd3 and of *Bdhn*4-*Bdhn*5 in Bd4 (only *B. distachyon*), and a segmental duplication of *Bdhn*1-*Bdhn*2 in Bd3 and Bd5 (Figure 3, Supplementary Table S3). *B. stacei* and *B. hybridum* S subgenome and *B. sylvaticum* showed a tandem duplication of *Bdhn*7-*Bdhn*8 in Bs4 and Bsy4, and a segmental duplication of *Bdhn*1-*Bdhn*2 in Bs4 and Bs9 and in Bsy4 and Bsy9, respectively. In addition, *B. sylvaticum* showed a tandem duplication of *Bdhn*1a and *Bdhn*1b in Bsy9 not found in the other *Brachypodium* species studied (Figure 3, Supplementary Table S3). Overall, all the selection tests performed with branch-sites models (aBSREL (adaptive Branch-Site Random Effects Likelihood) and BUSTED (Branch-Site Unrestricted Statistical Test for Episodic Diversification) for internal branches only and for leaf branches only) failed to detect evidence of positive selection in all the *Bdhn* genes and species all (*p*-values >0.05) except for two significant cases. aBSREL tests for internal branches only detected evidence of positive selection in *Bdhn*6 and *Bdhn*10 (one branch each) where ω2 rate class values were >1 but for very low percentages of tree branches (*Bdhn*6: *p* = 0.04, ω1 = 0.140 (97%), ω2 = 53.9 (2.6%); *Bdhn*10: *p* = 0.02, ω1 = 0.0 (99%), ω2 = 100,000 (1.1%); Supplementary Materials S1). Whereas aBSREL tests for positive selection modeling both site-level and branch-level ω heterogeneity, BUSTED performs a gene-wide (and not site-specific) test for positive selection. Similarly, selection tests performed at sites [MEME (Mixed Effects Model of Evolution)] did not detect evidence of positive selection at any site across the sequences of the *Bdhn* genes except for two significant or marginally significant positions in genes *Bdhn*1-*Bdhn*2 (site 139, *p* = 0.04; site 163, *p* = 0.02) and two in *Bdhn*6 (site 266, *p* = 0.04; site 412, *p* = 0.05) (Supplementary Materials S1). The greater power of MEME indicates that selection acting at individual sites is considerably more widespread than constant models would suggest. It also suggests that natural selection is predominantly episodic, with transient periods of adaptive evolution masked by the prevalence of purifying or neutral selection on other branches.

### 2.5. Dehydrin Gene Clusters, Phylogenetics, and Climate Niche Variation in B. distachyon

**Figure 4:**
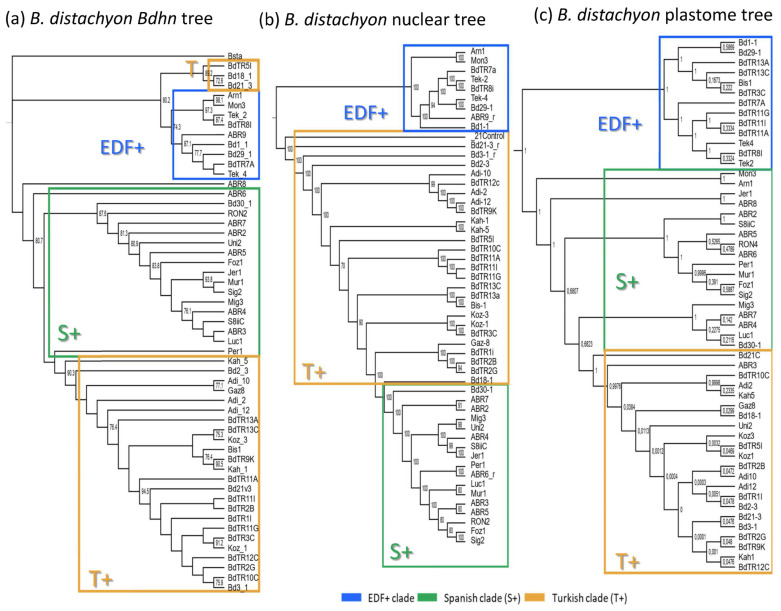
(**a**) Maximum likelihood *B. distachyon Bdhn* tree. Consensus tree constructed for 54 *B. distachyon* ecotypes from concatenated exon and intron aligned sequences of six dehydrin genes (*Bdhn*1, *Bdhn*2, *Bdhn*3, *Bdhn*6, *Bdhn*7, and *Bdhn*8). The EDF+ (extremely delayed flowering time, blue) clade, T+ (Turkish and East Mediterranean, orange), and S+ (Spain and West Mediterranean, green) lineages correspond to those indicated in [37,38]. Bootstrap support is indicated on branches. Accession codes correspond to those indicated in Supplementary Table S4. (**b**) *B. distachyon* nuclear species tree of [37] based on nuclear genome-wide 3.9 million SNPs. (**c**) *B. distachyon* plastome tree of [38] based on full plastome sequences.

The genomes of 54 *B. distachyon* ecotypes distributed across the Mediterranean region (Supplementary Table S4) were annotated for the 10 *Bdhn* gene clusters for comparative genomics, evolutionary, and phylogenetic signal of drought-related traits and climate niche analyses. Most ecotypes (74.07%) contained all 10 *Bdhn* genes (Supplementary Figure S3) and were used for downstream analyses. Independent ML trees obtained for each separate *Bdhn* gene, based on exon and intron sequences, showed differently resolved topologies (Supplementary Figure S4). Six of those 10 gene trees (*Bdhn*1, *Bdhn*2, *Bdhn*3, *Bdhn*6, *Bdhn*7, *Bdhn*8) recovered a congruent topology for some *B. distachyon* groups. A ML tree constructed from their concatenated sequences produced a single combined *B. distachyon Bdhn* tree (Figure 4a) showing a resolution similar to those observed previously in the *B. distachyon* nuclear-SNPs [37] (Figure 4b) and plastome [38] (Figure 4c) trees. The *Bdhn* tree revealed a relatively well-supported EDF+ clade (74% BS) and the successive but weakly supported divergences of the S+ and T+ lineages, with a clade of T+ lineages (Bd18-1, Bd21-3, BdTR5i) resolved as sister to the EDF+ clade (Figure 4a). Topological congruence Kishino-Hasekawa (KH), Shimodaira-Hasekawa (SH), and Shimodaira Approximately Unbiased (AU) tests performed between the *B. distachyon Bdhn* tree and the nuclear-SNP and plastome trees indicated that the topology of the *Bdhn* tree did not significantly differ (*p* < 0.001) from the topologies of the two compared trees (Supplementary Table S5), indicating that all three data sets recover congruent evolutionary histories for the divergences of the main *B. distachyon* lineages. However, the *Bdhn* tree visually resembled more the plastome tree than the nuclear tree (Figure 4).

Climate niches were constructed for the studied *B. distachyon* ecotypes to explore if they present differences in climate niche parameters that could be related to distinct responses to drought. The optimal climate niche of each *B. distachyon* ecotype was inferred from PCA of 19 climate variables (Supplementary Table S6 and Figure S5). The main PC1 axis (51.8% of variance) values allowed us to classify the *B. distachyon* ecotypes into cold (>2.5; ABR2, ABR3, ABR4, ABR5, ABR6, RON2), warm (<2.5; Bd2-3, Bd21, Bd3-1, Bis1, Kah1, Kah5, Koz1, Koz3), and mesic (−2.5 to 2.5, remaining) climate class ecotypes.

### 2.6. Differential Expression of Bdhn Genes in Brachypodium distachyon Ecotypes under Drought and Temperature Stress Conditions

We made use of data from an extensive transcriptome study of 32 *B. distachyon* ecotypes [39], which enabled us to explore intraspecific variation in how *Bdhn* genes are expressed in response to drought in mature plants. Development and tissue specific expression analysis of dehydrin genes was performed in 32 out of the 54 genomically sequenced ecotypes of *B. distachyon* (Supplementary Table S4). Only 4 out of the 10 identified *Bdhn* genes were expressed in mature leaves of all 32 studied *B. distachyon* ecotypes: *Bdhn*1a (Bradi5g10860.1), *Bdhn*2 (Bradi3g51200.1), *Bdhn*3 (Bradi1g37410.1), *Bdhn*7 (Bradi3g43870.1); Supplementary Table S7. These annotated dehydrins showed significant differential expression (DE) levels between the watered (W) and dry (D) conditions, independently of temperature conditions in both separate CD-CW-HD-HW and averaged D vs. W comparative tests (Supplementary Table S8 and Figure S6a,b). By contrast, the dehydrin expressions did not show significant differences between cool (C) and hot (H) conditions under drought treatment, and only *Bdhn*3 and *Bdhn*7 showed significant differences in CW vs. HW conditions, though none of them did in averaged C vs. H comparative tests (Supplementary Figure S6a,c).

**Figure 5:**
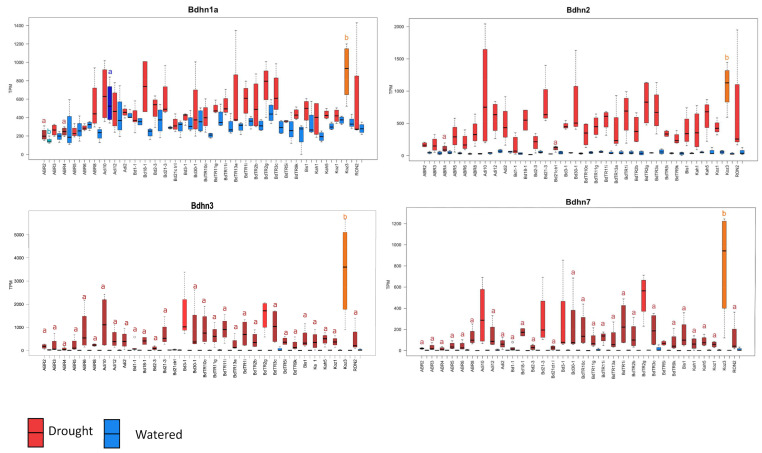
Differentially expressed *Bdhn*1a, *Bdhn*2, *Bdhn*3, and *Bdhn*7 dehydrin genes (transcript per million, TPM) in 32 ecotypes of *B. distachyon* under drought (D, red) vs. watered (W, blue) conditions. Different letters in the boxplots indicate significant group differences (Tukey HSD tests) (see also Supplementary Figure S7).

The four dehydrins showed significantly increased expression levels in drought conditions in most accessions (Wilcoxon tests, Supplementary Table S8; Tukey tests, Figure 5, Supplementary Figure S7). The DE levels were also significantly different among ecotypes, especially within the dry treatment, being highest in warm ecotypes Koz3 (*Bdhn*1a, *Bdhn*3, *Bdhn*7) and Adi10 (*Bdhn*2) and lowest in cold ecotypes ABR2 (*Bdhn*1) and ABR4 (*Bdhn*2, *Bdhn*3, *Bdhn*7) (Figure 5, Supplementary Figure S7). On average drought increased dehydrin expression by about 5.74% in *Bdhn*1a, 39% in *Bdhn*2, 67.8% in *Bdhn*3, and 97.8% in *Bdhn*7 compared to well-watered plants (Supplementary Figure S6b). Overexpression of dehydrins caused by drought stress was significantly correlated between all *Bdhn* gene pairs (Supplementary Table S9 and Figure S8).

A *B. distachyon*–*T. aestivum* DE comparative analysis found that the drought-induced *Bdhn*1, *Bdhn*2, *Bdhn*3, and *Bdhn*7 genes belong to the same ortholog groups as 15 out of the 16 differentially expressed wheat dehydrin (DHN) genes (Supplementary Table S10) under natural field drought stress [40] or greenhouse imposed drought stress [6,41]. In wheat there is a physical clustering of several dehydrin genes in two gene clusters located in the 5L and 6L groups of wheat chromosomes (Supplementary Table S10; Supplementary Figure S9). The 6L cluster contains 25 dehydrins and includes nine DHN3 genes and three DHN4 genes (all orthologs to *Bdhn*3), whereas the 5L cluster has 13 DHN38 genes of which six are orthologs to *Bdhn*7. In addition, the DHN11 genes, located in another portion of 6L chromosomes are orthologs to the *Bdhn*1 and *Bdhn*2 genes. We observed that orthologs from *B. distachyon* and *T. aestivum* tended to show a similar pattern of expression response to soil drying. Specifically, the duplicated *Bdhn*1 and *Bdhn*2 genes and the DHN11(A1) genes, the *Bdhn*7 gene and the duplicated DHN38 (B1, B2) genes, and the *Bdhn*3 gene and the duplicated DHN3 (A1, A6, B6, D1, D4, D6, D8, D9) genes were all upregulated in drought treatments (Supplementary Table S10 and Figure S9; [40]).

### 2.7. Effects of Drought on Dehydrin Gene Expression and Drought-Response Phenotypic Traits

The potential effect of drought on dehydrin expression levels and on correlated changes of phenotypic and physiological drought-response traits of the plants was evaluated in 32 *B. distachyon* ecotypes. The 12 drought-response phenotypic traits studied (leaf_rwc: relative water content in leaf; leaf_wc: water content in leaf; lma: leaf mass per area; pro: leaf proline content; abvgrd: above ground biomass; blwgrd: below ground biomass; ttlmass: total plant mass; rmr: root mass ratio; delta13c: carbon isotope, a proxy for lifetime integrated WUE; leafc: leaf carbon content; leafn: leaf nitrogen content; cn: leaf carbon/nitrogen ratio) also showed significant different values in dry vs. watered conditions across ecotypes (Supplementary Table S11). On average, drought significantly decreased the values of six traits (17.14% in abvgrd, 34.78% in ttlmass, 4% in leaf_rwc, 36.5% in leaf_wc, 12.5% in leafn, 2.8% in WUE) and significantly increased those of five traits (33% in pro, 5.5% in rmr, 2.96% in leafc, 5.71% in lma, 21.4% in cn) compared to watered conditions, but did not significantly affect the blwgrd trait (Supplementary Figure S10) [2].

Drought-induced effects caused significant positive and negative correlations between the averaged expressed values of the four *Bdhn* genes and changes in most phenotypic trait values (Supplementary Table S12 and Figure S11). Regression models for independent correlations between the *Bdhn*1a, *Bdhn*2, *Bdhn*3, and *Bdhn*7 expressions and the changes in the 12 phenotypic traits showed significant positive correlations for most dehydrin *Bdhn* genes with pro, blwgrd, rmr, WUE, leafc, and cn, negative correlations with leaf_rwc, leaf_wc, and leafn, and non-significant correlations with ttlmass and abvgrd (Supplementary Figure S11).

### 2.8. Phylogenetic Signal of Dehydrin Expression, Phenotypic Trait Changes and Climate Variation in the Brachypodium distachyon Bdhn Tree

**Figure 6:**
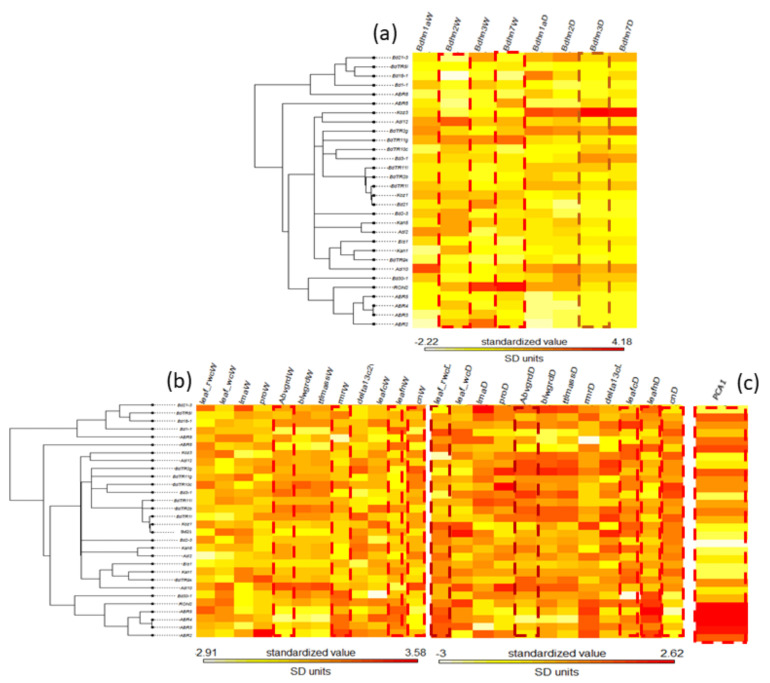
Maximum likelihood *B. distachyon Bdhn* tree cladogram showing the relationships of 30 ecotypes. Phyloheatmaps of normalized values for different sets of variables: (**a**) dehydrin (*Bdhn*1, *Bdhn*2, *Bdhn*3, *Bdhn*7) gene expression values under watered (W) and drought (D) conditions; (**b**) drought-response phenotypic traits (leaf_rwc: relative water content in leaf; leaf_wc: water content in leaf; lma: leaf mass per area; pro: leaf proline content; abvgrd: above ground biomass; blwgrd: below ground biomass; ttlmass: total plant mass; rmr: root mass ratio; delta13c: carbon isotope, a proxy for lifetime integrated WUE; leafc: leaf carbon content; leafn: leaf nitrogen content; cn: leaf carbon/nitrogen ratio) values under watered (W) and drought (D) conditions; (**c**) climate niche PC1 values. Traits showing significant phylogenetic signal are highlighted with dotted lines (see Supplementary Table S13b). Watered (W): soil irrigated to field capacity every second day; Drought (D): soil water content reduced by ~5% each day (during the 10 days experiment).

The potential phylogenetic signal of dehydrin expression, phenotypic trait changes, and climate variation was evaluated on both the *B. distachyon* nuclear species tree [37] and the *B. distachyon* dehydrin *Bdhn* tree. None of the dehydrin gene expression values under W or D conditions and few phenotypic and climate traits had significant K or lambda values on the *B. distachyon* nuclear species tree (Supplementary Table S13a and Figure S12). By contrast, *Bdhn*2W, *Bdhn*7W, and *Bdhn*3D expression values, phenotypic change of rmrW, leafnW, cnW, abwgrdW, leafcD, leafnD, and cnD traits’ values and climate niche PCA1 values carried phylogenetic signal (or marginal phylogenetic signal for leaf_rwcD and abvgrdD values) when tested on the dehydrin *Bdhn* tree (Supplementary Table S13b, Figure 6).

## 3. Discussion

### 3.1. The Dehydrin Gene Family in Brachypodium

Our comparative genomic analysis of the dehydrin genes in the reference genomes of four *Brachypodium* species and in the genomes of 54 ecotypes of *B. distachyon* has allowed us to identify 47 *Bdhn* genes. This is almost twice the number of LEA2 genes previously found in *B. distachyon* Bd21 [35]. Orthology and evolutionary analysis indicate that most of these proteins were probably present in the ancestor of the grasses (Supplementary Table S1; Figure 2). In contrast to previous non-monophyletic infra-generic phylogenies of dehydrin genes (e.g., *Oryza*; [9]), the *Brachypodium* dehydrin tree showed 8 out of 10 monophyletic and highly supported *Bdhn* clades (Figure 2). Segmental and tandem duplications of ancestral *Bdhn*1-*Bdhn*2 and recent *Bdhn7*-*Bdhn*8 genes have been detected in all studied *Brachypodium* species (Figure 2 and Figure 3; Supplementary Figure S3); the tandemly duplicated *Bdhn*7 and *Bdhn*8 genes probably evolved, in turn, from a duplication of an ancestral *Bdhn*3 gene through insertion/deletions (*Bdhn*7-8) and the loss one K-segment (*Bdhn*8) (Figure 1c and Figure 2). However, *Bdhn*4 is inferred to have originated from a tandem duplication of *Bdhn*5 exclusively in the ancestor of the *B. distachyon* lineage and *Bdhn*1b from a tandem duplication of *Bdhn*1a in the *B. sylvaticum* lineage (this Supplementary Table S3 and Figure S3; Figure 2). The allotetraploid *B. hybridum* exhibits homeologous copies inherited from its diploid progenitor species for the same sets of tandemly and segmentally duplicated genes (Table 1; Figure 2 and Figure 3). Nonetheless, the loss of the *Bdhn*4 gene in its D subgenome probably occurred after the hybridization and whole genome duplication (WGD) event that generated this reference genome [30,42], as this gene is largely present in the *B. distachyon* ecotypes studied (Supplementary Figure S3). Our data support the hypothesis of a highly dynamic evolution of duplications and losses of dehydrin paralogs in *Brachypodium*. This was also evident in other grasses such as *Hordeum vulgare* [43], *Triticum aestivum* [7], and some *Oryza* species [9].

The consensus ML phylogenetic tree of *Brachypodium* species based on 10 *Bdhn* genes (Figure 2) depicted a congruent topology in seven gene clades (*Bdhn*1, *Bdhn*3, *Bdhn*(4)5, *Bdhn*7, *Bdhn*8, *Bdhn*10), resulting in more ancestral *B. stacei/B. hybridum-*S sequences followed by the split of more recent *B. distachyon/B. hybridum-*D and *B. sylvaticum* copies. This resolution was fully congruent with that of the *Brachypodium* species tree [44]; the branch swaps observed in the three remaining *Bdhn*2, *Bdhn*6, and *Bdhn*9 clades (Figure 2) likely resulted from incomplete lineage-sorting events. Therefore, the evolution of the dehydrin genes was in pace with the organismal evolution of the *Brachypodium* lineages, supporting their species-level evolutionary synchrony. Although the *Bdhn* intraspecific phylogenies of *B. distachyon* ecotypes were more variable or unresolved (Supplementary Figure S4), the consensus topology of the combined *B. distachyon Bdhn* tree and its congruence with the nuclear and plastome trees indicates that the conserved dehydrin genes also track the fast divergences of the recent *B. distachyon* ecotypes (Supplementary Table S5; Figure 4). Selection tests of *Bdhn* genes have consistently failed to detect evidence of positive selection at branch-sites or sites, including the duplicated *Bdhn* genes (Supplementary Materials S1). Our results indicate that all *Brachypodium* dehydrins are likely functional and that the duplicated paralogs are under selective constraint, irrespective of their ancestral or recent origins (Figure 2).

The amino acid composition, structure, and biochemical features inferred for the *Brachypodium* dehydrins (Supplementary Table S2) support their potential roles as regulators of the water-deficit in the cells [8,9,10]. *Bdhn* dehydrins variation in size, molecular weight, and pI and their GRAVY values fall within those observed for rice dehydrins [9]. The large differences in *Bdhn* pI values [≤6 (*Bdhn*1, 2, 9)–>9 (*Bdhn*3, 4, 5, 6, 7, 8)] suggest that those proteins may be located in specific compartments of the cell, like the cytoplasm and the nucleus. *Bdhn* dehydrins with high pI values and with phosphorylated S-segments (Y_n_SK_n_; e.g., *Bdhn*3, *Bdhn* 4, *Bdhn*5, *Bdhn* 6, *Bdhn*7, *Bdhn*8) may bind negatively charged molecules such as DNA and NLS proteins [8]. The *Bdh*10 dehydrins (HIRD11 family), which lack the K-segment, could bind different ions and reduce the formation of reactive oxygen species (ROS), like that observed for the AtHIRD11 ortholog [26]. The three-domain complex architecture of *Bdhn*9 (Dna-J–Dna-JX–DHN) has been also observed in other grass dehydrins, like DHN1 of rice [9] and *Setaria italica* [45]; the DnaJ domain may have a chaperone function [9].

### 3.2. Dehydrin Expression Induction in Brachypodium distachyon

Dehydrin expression varies considerably both in plant tissue and developmental stage and under different abiotic stress conditions [7,8,9]. Most DHN genes have shown high expression profiles in seeds and immature seedling stages in wheat and rice [7,9]; however, their expression decreases in mature or late-development tissues or is even absent in some organs, like mature leaves in rice [9]. Although the expression data was restricted to mature leaf tissue, our analyses demonstrated that four (*Bdhn*1a, *Bdhn*2, *Bdhn*3, *Bdhn*7) out of the ten detected *Brachypodium Bdhn* dehydrin genes were constitutively and inductively expressed in mature leaves of *B. distachyon* plants (Supplementary Tables S7 and S8 and Figures S6 and S7; Figure 5). In silico and RT-PCR expression analysis showed that YSK2-type dehydrins were upregulated in drought-stressed shoots of wheat, whereas Kn-type dehydrins were preferentially expressed in cold-stressed shoots [7]. Other drought-PEG treatments have shown considerable upregulation of eight DHN genes in rice shoots [9]. The effect of drought significantly contributed to transcriptional upregulation of the four genes in dry vs. watered plants in all *B. distachyon* ecotypes (Figure 5), irrespective of the temperature treatment (Supplementary Figures S6 and S7). This analysis demonstrated a 2.5–21.5-fold increase in the expression (TPM) of *Bdhn*1a but a much higher and variable 2–490-fold increase in those of *Bdhn*2, *Bdhn*3, and *Bdhn*7 under dry than under water conditions (Supplementary Table S8 and Figure S6; Figure 5). *Bdhn*1a and *Bdhn*2 are (FS)K_n_-type genes whereas *Bdhn*3 and *Bdhn*7 are Y_n_SK_n_-type genes (Table 1; Figure 2); thus our results partially depart from those found in wheat by [7] suggesting that both types of genes are preferentially upregulated by drought rather than by temperature in mature leaves of *B. distachyon*. By contrast, our comparative structural analysis between *B. distachyon* differentially expressed *Bdhn* genes in this work, and the previously reported wheat dehydrin genes differentially expressed in flag leaves under natural field [40] or greenhouse-imposed [6,41] drought stress, inferred that 15 out of the 16 DE wheat dehydrins belong to the same *Brachypodium* DE ortholog groups (Supplementary Table S10 and Figure S9). These shared differential gene expression responses to drought by orthologous dehydrin genes’ induction in both species reinforce the potential of *B. distachyon* as a model system for cereals such as bread wheat. Our results also highlight the likely importance of these four dehydrins in the protection of *B. distachyon* plants to water stress conditions in mature individuals, the developmental stage when they face the most severe drought conditions of their life cycle [46].

The drought-induced upregulation of *Bdhn* genes was different among the studied *B. distachyon* ecotypes for all four *Bdhn* genes (Supplementary Table S8 and Figure S7; Figure 5). Recent analysis of dehydrin gene expression in *B. distachyon* and its close *B. stacei* and *B. hybridum* species have shown that Bradi1g37410 (*Bdhn*3) is strongly upregulated by drought (>400-fold higher expression in dry plants compared to control plants), although the level of induction depended on genotype [47]. Our data support these results and additionally demonstrate that drought induction also upregulates the expressions of the *Bdhn*1a, *Bdhn*2, and *Bdhn*7 genes and that increased expression is most evident in the warm climate ecotypes (Koz3, Adi10) and less so in the cold climate ecotypes (ABR2, ABR4), whereas mesic climate ecotypes show intermediate expression levels (Supplementary Table S8 and Figure S7; Figure 5). Earlier classification of *B. distachyon* ecotypes into drought-tolerant, -intermediate, and -susceptible, based on phenotypic plant water content and wilting index values [48], roughly correspond to our warm, mesic, and cold ecotype climate classes, thought their plants were subjected to uncontrolled severe drought treatments which may have confounded plant size with soil water content. The significant differences found between the drought-induced dehydrin expression levels in our climate class *B. distachyon* ecotypes suggest that ecotypes adapted to warm climates may have developed higher *Bdhn*1a, *Bdhn*2, *Bdhn*3, and *Bdhn*7 expression responses as a strategy to protect the mature plants against harsh water deprivation conditions and to ensure the survival and reproduction of the individuals in their habitats. By contrast, in mesic and, especially, in cold climate adapted ecotypes those inductions are much lower possibly due to the absence or mitigated presence of the natural stressor.

The constitutive and induced expression of the stress-responsive dehydrins is upregulated by the presence of specific *cis*-regulatory elements in the promoter region of their genes [49]. Our de novo analysis of *cis*-regulatory elements performed in silico consistently found three TF-binding sites, BES1/BZR1, MYB124, and ZAT, across the *Bdhn* promoters of the studied *Brachypodium* species, and more abundantly in the promoter regions of the *Bdhn*1a and *Bdhn*2 genes (Table 2; Supplementary Figure S2a). BES1/BZR1 is a brassinosteroid signaling positive regulator (BZR1) family protein involved in the regulation pathway in response to drought [50]. The MYB gene protein MYB124 is related to the abscisic acid (ABA) response, an hormone-regulated pathway implicated in multiple stress response such as drought or cold stress [51,52]. The ZAT C2H2 zinc finger is involved in response to salinity stress [53]. The presence of these *cis*-regulatory elements in the promoters of most *Bdhn* genes suggest that these dehydrins could be highly upregulated in *Brachypodium* plants under different environmental stressors such as drought, cold, and salinity, and that MYB124 and BES1 may play an important role in the induction of the *Bdhn*1a and *Bdhn*2 genes in the studied ecotypes, especially in those adapted to warm climates.

### 3.3. Correlated Dehydrin and Phenotypic Drought Response and Phylogenetic Signal in Brachypodium distachyon

Water deficit stress affects the physiology, the phenotypes, and the fitness of plants [1,2]. As shown earlier by [2], drought effect significantly influenced the changes of *B. distachyon* ecotypes’ phenotypic traits, reducing the water contents, total plant mass, and leaf nitrogen content of dry plants but increasing their root biomass, leaf carbon content, proline, and WUE (Supplementary Table S11 and Figure S10). Our linear regression models indicated that the expressions of the four *Bdhn* genes were significantly correlated with the changes of most phenotypic traits in the drought treatment (Supplementary Table S12 and Figure S11). The high correlations observed between the expression of *Bdhn*1a, *Bdhn*2, *Bdhn*3, and *Bdhn*7 genes and the decrease of leaf water and nitrogen contents and the increase of belowground biomass, root mass, WUE, and leaf carbon and proline content were strongly associated to drought stress. Proline can serve as an osmoprotectant and a signaling molecule triggering adaptive responses to cell water stress. Martinez et al. reported a significant decrease in plant water content but no significant changes in proline content in *B. distachyon* ecotypes under drought stress [46]. By contrast, Fisher et al. found significant differences in both traits across *B. distachyon* ecotypes under mild and severe drought treatments, with drought-tolerant ecotypes showing more prominent water and proline responses than the drought-intermediate and drought-susceptible ecotypes [48]. Our data corroborate the last results and further illustrate that drought-induced proline production is significantly higher in warm-to-mesic climate *B. distachyon* ecotypes (Adi10, Koz3) and lower in cold climate ecotypes (ABR5, ABR4, ABR3) (Supplementary Table S11) and that those differences overlap with the significant differences observed in their dehydrin overexpression profiles (Supplementary Table S8; Figure 5). In cool seasonal plants WUE is expected to increase with aridity [54]. However, Manzaneda et al. found that drought-avoider *B. distachyon* ecotypes showed lower WUE than aridic drought-escape *B. hybridum* ecotypes, although the former had higher values of WUE plasticity related to climate than the second [55]. Des Marais et al. also found an association of WUE with climate as *B. distachyon* ecotypes from cooler climates were more plastic in their WUE than those from warmer climates [2]. Our data indicate that the overall dehydrin expression is significantly correlated with WUE (Supplementary Figure S11). In addition, WUE shows a great plasticity across ecotypes of any climate class and under both drought and watered conditions (Supplementary Table S12).

Phylogenetic signal measures the statistical dependence among species’ trait values due to their phylogenetic relationships [56]. The potential evolutionary signal of dehydrin expression values and phenotypic trait values gave different results when tested on the *B. distachyon* nuclear species tree or the *B. distachyon Bdhn* tree. The absence of phylogenetic signal for the dehydrin expressions and the residual signal for some of their associated drought-response phenotypic traits in the nuclear species tree (Supplementary Table S13a and Figure S12) indicates that these drought-response mechanisms may have evolved independently and at different times along the life history of *B. distachyon*. However, several flowering time traits and their molecular regulators have shown a strongly correlated evolution with the nuclear species tree [37], supporting the important role of flowering time in shaping the divergences of the *B. distachyon* lineages. Conversely, the significant phylogenetic signal of some dehydrin expressions, drought response phenotypic traits changes, and climate niche data variation on the *B. distachyon Bdhn* tree (Supplementary Table S13b; Figure 6) suggests that the evolution of the *Bdhn* genes is determined by the adaptation of the *B. distachyon* ecotypes to more dry or more wet environmental conditions. It is surprising the high topological similarity found between the *Bdhn* tree and the plastome tree in contrast with its more dissimilar topology with respect to the nuclear species tree (Figure 4) for nuclear dehydrin genes that encode cytoplasmic and nuclear but not chloroplast proteins [8]. The relative congruence detected between the *Bdhn* and plastome trees could be a consequence of incomplete lineage sorting events of the recently evolved *B. distachyon* lineages [38]. However, it could also imply a yet unknown organellar effect on the cellular response mechanism to adaptation to drought, like the role played by the chloroplast in inducing the expression of nuclear heat-response genes during heat stress in plants [57]. Our data open new ways to investigate the potential implication of this organelle in the induction of drought-response nuclear genes, like those encoding for dehydrins, and in their evolutionary history.

## 4. Material and Methods

### 4.1. Identification of Dehydrin Sequences

Dehydrins of *B. distachyon*, *B. stacei*, *B. hybridum*, *B. sylvaticum*, and other grass outgroups (Aegilops tauschii, Hordeum vulgare, Oryza sativa, Sorghum bicolor, Triticum aestivum, and Zea mays) were identified using three searching approaches. First, the Phytozome v.12.1 database [58]) was searched for DHN gene sequences of *B. distachyon*, *B. stacei*, *B. hybridum*, and *B. sylvaticum* (Table 1). Phytozome dehydrin sequences were retrieved using BioMart to filter sequences having DHN Pfam code (Pfam00257). This search was repeated in the Ensembl [59] and Genbank [60] databases, aiming to retrieve all dehydrin genes present in Brachypodium. Redundant sequences and incomplete transcripts were deleted. Second, a consensus K-segment was used as a query sequence to search for complete DHN genes within the retrieved sequences using the BlastP tool [61]. The presence of a K-segment with a maximum threshold of 4 mismatches with respect to the query was used to characterize a protein sequence as a dehydrin. Third, orthologous dehydrin genes from six additional grass species with complete sequenced genomes were searched in Ensembl Plants, Phytozome, Genbank, and Panther (http://pantherdb.org/data/, 29 November 2021) databases using BioMart and used as reference outgroups. The Pfam00257 code was used to find DHN orthologous sequences from the six outgroup species, discarding also redundant sequences or sequences without the K-segment.

The theoretical molecular weight (mol. wt), isoelectric point (pI), instability index, and grand hydrophathicity average index (GRAVY) values of the different dehydrins were predicted using the ProtParam tool (http://web.expasy.org/protparam, 29 November 2021). In silico dehydrin structures were modeled using the web server version of RaptorX (http://raptorx.uchicago.edu/BindingSite/, 29 November 2021; [62,63]) and their structural properties were analyzed using Icn3D 3D structure viewing tool [64].

### 4.2. Structural Analysis, Conserved Motifs, and Cis-Regulatory Elements (CREs)

The inferred DHN polypeptide sequences were used to analyze the presence of conserved motifs and to characterize the structure of the dehydrins. A custom search tool (Supplementary Data, https://github.com/Bioflora/Brachypodium_dehydrins (accessed on 28 October 2021)) was designed to find the conserved K (EKKGIMDKIKEKLPG), Y (VDEYGNP), S (SSSSS+), ϕ (EDDGQGR), F (DRGLFDKFIGKK), and NLS (KKDKKKKKEKK) motifs present in the dehydrin domain. A consensus sequence for each segment was retrieved and used as a query in a BLASTP search (https://blast.ncbi.nlm.nih.gov/Blast.cgi?PAGE=Proteins, 29 November 2021), allowing a maximum threshold of 4 mismatches with respect to the query. The dehydrin architectures were established according to the presence and distribution of their conserved motifs.

De novo discovery of CREs was performed on windows of –500-to-+200 bp both sides of the transcriptional start site (TSS) of DHN genes in all the studied *Brachypodium* species and ecotypes. We searched for over-represented motifs using RSAT::Plants [65] tool peak-motifs, as described in [66]. This analysis was run four times, using as genome background model the respective reference genome of each *Brachypodium* species under study (*B. distachyon* Bd21 v3.0.46. JGI, *B. stacei* ABR114 v1.1.JGI, *B. hybridum* ABR113 v1.1.JGI, *B. sylvaticum* Ain1v1.1.JGI). Significant enrichment of the discovered motifs was assessed using as negative control promoters from the same number of randomly picked genes [66]. Candidate motifs were chosen based on their k-mer significance and number of sites and subsequently clustered to avoid redundancies using the matrix-clustering tool [67]. A total of 60 potential *cis*-regulatory motifs were retrieved and subsequently filtered, comparing their k-mer significance and number of sites with the negative controls (Supplementary Figure S2b). Of them, 29 motifs were clustered to avoid redundancies due to different identifications of the same CRE. Selected motifs were finally scanned along each *Bdhn* promoter to locate potential CREs using a matrix scan and a maximum threshold of 9 based on the median length of the 3 motif logos (Table 2).

### 4.3. Multiple Alignments and Phylogenetic Analysis

Multiple sequence alignment (MSA) of the nucleotide coding sequences of all the *Brachypodium* species and other grass outgroups’ dehydrin genes was performed with ClustalW in MEGA v.5 [68] using default settings. The start codon of each dehydrin gene was set using the Phytozome annotations and the sequences were adjusted manually to fit the reading frames. Alignments of dehydrin sequences, including exons and introns were performed with MAFFT v.7.215 [69] in Geneious Prime 2021 (https://www.geneious.com/prime/ (accessed on 28 October 2021)). These alignments and their respective coding sequences were used for downstream phylogenetic analyses and for the selection tests. There was no missing data in any of the single alignments; the alignment of combined data from the six *Bdhn* genes (*Bdhn*1, *Bdhn*2, *Bdhn*3, *Bdhn*6, *Bdhn*7, *Bdhn*8) used to reconstruct the *B. distachyon Bdhn* tree showed an extremely low percentage of missing data (2.7%) caused by the lack of dehydrin copies in some accessions. Maximum likelihood (ML) phylogenetic trees were constructed with IQTREE 1.6.12 [70] imposing the best-fit nucleotide substitution model of each data set according to the Bayesian Information Criterion (BIC). Branch support for the best tree was estimated through 1000 ultrafast bootstrap replicates.

### 4.4. Chromosomal Location, Gene Duplication, and Selection Analysis

Physical locations of the *Brachypodium* dehydrin genes in the 5 chromosomes of *B. distachyon*, 10 of *B. stacei*, and 9 of *B. sylvaticum* were obtained from Phytozome and Ensembl. They were mapped to their respective chromosomes using gff3 annotation coordinates for each dehydrin gene. Tandemly and segmentally duplicated genes were identified on the chromosomes; tandemly duplicated dehydrin genes were those distributed adjacent to an homologous dehydrin gene on the same chromosome or within a sequence distance of 50 kb [71].

The signature of positive selection (ω > 1) on each *Bdhn* gene for the four studied *Brachypodium* species (five genomes/subgenomes) was tested through both branch-site (aBSREL [72], BUSTED [73]) and site (MEME [74]) tests with Datamonkey2 [75,76]; https://www.datamonkey.org/, accessed on 29 November 2021). aBSREL and BUSTED models searched for positive selection at all sites and internal branches only or leaf branches only across the entire phylogeny. MEME (Mixed Effects Model of Evolution) tested for potential diversifying selection at individual sites under a proportion of branches.

### 4.5. Clustering and Phylogeny of Dehydrin Genes in Brachypodium distachyon Ecotypes

Annotations of the locations of dehydrin genes in the genomes of 54 *B. distachyon* ecotypes (Supplementary Table S3) were used to map them into the five *B. distachyon* chromosomes using a custom tool (Supplementary Materials, https://github.com/Bioflora/Brachypodium_dehydrins (accessed on 28 October 2021)). Protein sequences were obtained from primary transcript files and specific dehydrin genes were extracted from pseudomolecules using coordinates from gff3 annotation files. The dehydrin protein sequences were aligned using BLOSUM62. Dehydrin genes of all ecotypes were classified into different clusters based on a similarity threshold of 95%. Only clusters containing three or more sequences were selected. Unclassified sequences were iteratively compared to the previous blocks and classified into new clusters following the procedures of the first analysis. The remaining unclassified sequences were identified manually and classified as dehydrins whenever possible. Dehydrin *Bdhn*4 and *Bdhn*5 were annotated together in certain *B. distachyon* lines. Those sequences were manually curated and their presence in the genomes corroborated using BLASTN (Supplementary Materials S2) at Brachypan database [37]. Maximum-likelihood (ML) phylogenetic analysis was performed with coding and non-coding sequences of the ten *Brachypodium* dehydrin genes across the 54 ecotypes of *B. distachyon* using IQTREE and the procedures indicated above.

### 4.6. Expression Analysis of Dehydrin Genes in Brachypodium distachyon

Expression analysis of dehydrin genes was performed from a transcriptome study of 32 ecotypes of *B. distachyon* [39] (Supplementary Table S4) using replicates of the plant materials employed in the ecophysiological study of [2]. Seeds were stratified at 6 °C for two weeks and then grown in the greenhouse at soil field water capacity for 3 weeks. Light levels were set at 400–1000 lmol m_2 photosynthetically active radiation (PAR; mean of 825 lmol m_2) for 10 h d_1 (short-day conditions to prevent rapid flowering). Grown plants (21 days from initial pot emergence) were subjected to watered (W) vs. dry (D) conditions for a 10 day experiment, following the experimental design described in [2] (see Supplementary Materials Table S3 for more details). Irrigated plants were watered to soil field capacity every second day whereas soil water content was reduced by ~5% each day in dry plants. The plants under both treatments were simultaneously exposed to cool (C, daytime ~25 °C) or hot (H, daytime ~35 °C) conditions, however the temperature stress conditions did not affect substantially the expression of dehydrin genes (see Section 2). Fully expanded leaves from 31-days old individuals (four replicates) per ecotype and treatment were excised below the lamina, flash-frozen on liquid nitrogen and then stored at −80 °C until RNA extraction. RNA isolation was performed using the Sigma Total Plant RNA kit. RNA-Sequencing of 3′ cDNA tag libraries (with fragment of 300–500 bp) was conducted on an Illumina HiSeq2500 platform obtaining 100 bp Single-End (SE) reads. This method yielded only one sequence per expressed transcript in the RNA pool, allowing for higher sequencing coverage per gene [39]. SE reads were checked for quality with FastQC and adapters and low quality reads were removed and filtered with Trimmomatic-0.32 [77]. Total TPM values were quantified with Kallisto v0.43.1 [78], normalized with Sleuth [79], and annotated for dehydrins using the *B. distachyon* Bd21 v.3.1 reference genome (http://phytozome.jgi.doe.gov/, 29 November 2021; [80]). TPM values of annotated dehydrins of plants under the combined WC, WH, DC, and DH treatments were extracted from the large TPM abundance database (Supplementary Table S7). The *B. distachyon* RNAseq data were deposited in the European Nucleotide Archive (ENA; https://www.ebi.ac.uk/ena(accessed on 28 October 2021)) under accession codes ERR6133302 to ERR6133575 (project PRJEB45871) and those of *Bdhn* genes in Github (https://github.com/Bioflora/Brachypodium_dehydrins (accessed on 28 October 2021)).

Summary statistic (mean, median, SD, range) values and boxplots of differentially expressed (DE) dehydrin (TPM) data were computed for each ecotype and expressed dehydrin gene using the stats package in R. Statistically significant differences between median values of samples under drought (W vs. D) and temperature (C vs. H) stresses, and within each of the W and D treatments of the drought experiment were tested with ggplot and geom_signif functions in R. Wilcoxon pairwise difference tests for all pairs of compared samples with *p*-values adjusted with the Benjamini–Hochberg procedure to correct for multiple comparisons, Kruskal–Wallis rank tests for the whole group of samples within each group, and posthoc Tukey tests for among ecotypes differences were computed using the base, dplyr, ggplot2, ggpubrr, ggsignif, lm FSA, car, and multicompView packages of R.

### 4.7. Drought-Induced Changes in Dehydrin Expressions, Phenotypic, Physiological, and Climatic Niche Traits, and Phylogenetic Signal in Brachypodium distachyon

Values of 12 drought-response traits under W and D treatments (leaf_rwc; leaf_wc; lma; pro; abvgrd; blwgrd; ttlmass; rmr; delta13c (WUE); leafc; leafn; cn) were measured in the same individual samples (replicates) used in the transcriptomic analyses (Supplementary Materials S4); these phenotypic characters corresponded to those studied by [2]. Summary statistics and significance tests were computed for the 12 traits under W and D treatments following the same procedures mentioned above. The *B. distachyon* dehydrin genes differentially expressed in leaves of plants under W and D conditions in this work were compared to the wheat dehydrin genes differentially expressed under drought conditions in previous transcriptomics analyses. The wheat RNAseq analyses were carried out in flag leaves of individuals under field drought stress [40] and field rain shelter and greenhouse experiments [6,41,81]. The reported DE wheat dehydrins were used to perform the comparisons. Thus, 60 wheat dehydrin gene sequences [82] were retrieved through Blast analysis from the Wheat@URGI portal https://wheat-urgi.versailles.inrae.fr, 29 November 2021, [83]. Orthology of the expressed *B. distachyon* and *T. aestivum* dehydrin genes was primarily retrieved from Ensembl Plants using BioMart, and only few cases were retrieved using Blast and the homoeologies previously established in [39] (Supplementary Table S10).

Environmental climate data was retrieved for the studied *B. distachyon* ecotypes from worldclim (19 temperature and precipitation variables; Supplementary Table S6). Climatic niche optima were constructed for each ecotype based on occurrence data and the first axis of the ordination of the climatic variables (PCA1) was computed with the dudi.pca function of the ade4 package [84] in R. The climatic niches of the *B. distachyon* ecotypes were classified in climatic classes warm, mesic, or cold according to their PCA1 eigenvalues (see Section 2; Supplementary Table S6).

To address potential correlations between the dehydrin gene expressions and the changes in drought-response phenotypic traits, linear regression model analyses were performed for testing the effect of particular *Bdhn* gene expression on phenotypic changes using the lm function of the R stats package.

A consensus ML phylogenetic tree of 30 *B. distachyon* ecotypes based on the expressed dehydrin genes (*Bdhn* tree) was topologically contrasted to that of the *B. distachyon* nuclear species tree based on genome-wide >3.9 million syntenic SNPs [37] using the KH, SH, and AU tests with resampling estimated log-likelihood (RELL) optimization and 1 million bootstrap replicates in PAUP* [85]. We also tested for topological congruence of the *Bdhn* tree and the *B. distachyon* plastome tree based on full plastome sequences of these ecotypes [38] using the same testing approach.

Dehydrin expression level, drought-response phenotypic change, and climatic niche (PCA1) variation traits were tested for phylogenetic signal using Blomberg’s K [86] and Pagel’s lambda [87] with the *phylosig* function of the package *phytools* [88] in R. For both tests, values close to 1 indicate that trait values are consistent with the tree topology (phylogenetic signal) and those close to 0 that there is no influence of shared ancestry on trait values (phylogenetic independence). Phylogenetic signal was assessed on both the *B. distachyon* nuclear species tree and the *B. distachyon Bdhn* tree. Phyloheatmaps were generated for the standardized values of these continuous characters with *phytools*.

## 5. Conclusions

We annotated and analyzed the ten *Brachypodium* dehydrin genes (*Bdhn*1–*Bdhn*10) present in the reference genomes of the three annual (*B. distachyon, B. stacei, B. hybridum*) and one perennial (*B. sylvaticum*) species of the genus. Most *Bdhn* genes have orthologs in other close grass species. Ancestral segmental and tandem duplications have been, respectively, detected in all species for the *Bdhn*1/*Bdhn*2 and *Bdhn*7/*Bdh*8 genes, and recent tandem duplications in *B. distachyon* for *Bdhn*4/*Bdhn*5 and in *B. sylvaticum* for *Bdhn*1a/*Bdhn*1b genes. Structural and biochemical properties of the *Brachypodium* dehydrins indicate that these disordered proteins may be present in the cytoplasmic and nuclear compartments of the cell. The three *cis*-regulatory elements identified in the promoter regions of the *Bdhn* genes suggests that the predominant regulation of the *Bdhn* genes is via ABA- and brassinosterioid-mediated response metabolic pathways. Only four dehydrin genes (*Bdhn*1a, *Bdhn*2, *Bdhn*3, *Bdhn*7) are expressed in mature laves of *B. distachyon*. Differential expression levels of these dehydrins are mainly induced by drought rather than temperature conditions and are genotype-dependent, being significantly higher in warm than in mesic or cold climate ecotypes. Drought-mediated dehydrin upregulation is significantly correlated with leaf water and nitrogen contents decreases and root biomass and leaf proline increase, which are also genotype-dependent. Reverse genetic experiments of over-expression or silencing of these differentially expressed *Bdhn* genes in *Brachypodium* would be an excellent avenue for future research to confirm their role in response to drought stress.

## Figures and Tables

**Table 1:** Dehydrin genes found in the studied *Brachypodium distachyon*, *B. stacei*, *B. hybridum* (subgenomes D and S), and *B. sylvaticum* species. *Brachypodium* dehydrin gene codes (*Bdhn*1–*Bdhn*10) given in this study, the protein structure and their corresponding Panther family and subfamilies gene codes. Phytozome dehydrin gene codes correspond to the respective gene numbers in the reference genome of each species deposited in Phytozome.

*Bdhn*	Structure	Panther Family	Panther Subfamily	Phytozome Dehydrin Gene Codes				
				*B. distachyon* (Bd21)	*B. stacei* (ABR114)	*B. hybridum* D (ABR113)	*B. hybridum* S (ABR113)	*B. sylvaticum* (Ain1)
*Bdhn1a*		PTH33346	ERD14	Bradi5g10860	Brast09G089800	Brahy.D05G0138300	Brahy.S09G0091400	Brasy9G143400
*Bdhn1b*	FSK_n_							Brasy9G149400
*Bdhn2*	FSK_n_			Bradi3g51200	Brast04G110500	Brahy.D03G0707100	Brahy.S04G0117200	Brasy4G117200
*Bdhn3*	Y_n_SK_n_		SF23	Bradi1g37410	Brast07G152200	Brahy.D01G0507100	Brahy.S07G0169500	Brasy7G144000
*Bdhn4*	Y_n_SK_n_		SF14	Bradi4g22280				
*Bdhn5*	Y_n_SK_n_			Bradi4g22290	Brast05G049400	Brahy.D04G0319400	Brahy.S05G0054400	Brasy5G271500
*Bdhn6*	Y_n_SK_n_		SF 19	Bradi4g19525	Brast05G075400	Brahy.D04G0277700	Brahy.S05G0083200	Brasy4G237500
*Bdhn7*	Y_n_SK_n_		SF14	Bradi3g43870	Brast04G194300	Brahy.D03G0604200	Brahy.S04G0208500	Brasy4G220100
*Bdhn8*	Y_n_SK_n_			Bradi3g43855	Brast04G197200	Brahy.D03G0604000	Brahy.S04G0208900	Brasy4G219800
*Bdhn9*	Y_n_SK_n_		XERO I	Bradi2g47575	Brast01G171900	Brahy.D02G0637300	Brahy.S01G0182800	Brasy1G228900
*Bdhn10*	K*NLSL-S	PTHR34941	HIRD 11	Bradi1g13330	Brast02G251900	Brahy.D01G017200	Brahy.S02G0268100	Brasy2G277500

**Table 2:** Upstream putative *cis*-regulatory elements (CREs) found in the promoter region (–500-to-+200 bp) of the *Bdhn* genes of Brachypodium distachyon, *B. stacei*, *B. hybridum* (subgenomes D and S), and *B. sylvaticum* using Rsat::plants tools and the corresponding reference genome as background. Family identification, motif code (ID), N-cor (normalized correlation), and Sig (significance value) for the highest hit, matches to transcription factor (TF) binding sites, and *Bdhn* genes with the number of sites found within each species and gene. Species and reference genomes: BD, *B. distachyon* Bd21; BS, *B. stacei* ABR114; BHD, *B. hybridum* subgenome D ABR113; BHS, *B. hybridum* subgenome S ABR113; BS, *B. sylvaticum* Ain1. Mapping positions of these *cis*-regulatory motifs are indicated in Figure 1b.

Family ID	Motif ID	Ncor	Sig	TF Binding Site	*Bdhn* Genes (No. Sites Found)
BRI1-EMS suppressor/brassinazole-resistant	BES1/BZR1	0.719	4.08	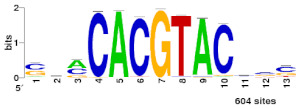	**BD**: *Bdhn*5(1) *Bdhn*6(1) *Bdhn*7(3) *Bdhn*9(1)
					**BHD**: *Bdhn*1(1) *Bdhn*2(2) *Bdhn*5(1) *Bdhn*6(1) *Bdhn*7(3) *Bdhn*9(1)
					**BHS**: *Bdhn*1(1) *Bdhn*2(2) *Bdhn*5(4) *Bdhn*6(1) *Bdhn*7(2) *Bdhn*8(1)
					**BS**: *Bdhn*1(1) *Bdhn*2(2) *Bdhn*5(3) *Bdhn*6(1) *Bdhn*7(2) *Bdhn*8(1)
					**BSY**: *Bdhn*2(1) *Bdhn*5(1) *Bdhn*7(1) *Bdhn*8(2) *Bdhn*9(1)
Myb	MYB124	0.598	2.18	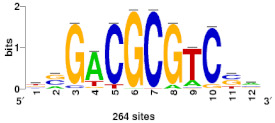	**BD**: *Bdhn*1(5) *Bdhn*2(4) *Bdhn*3(2) *Bdhn*6(1) *Bdhn*7(2) *Bdhn*8(2)
					**BHD**: *Bdhn*1(7) *Bdhn*2(4) *Bdhn*3(2) *Bdhn*6(1) *Bdhn*7(2) *Bdhn*8(2)
					**BHS**: *Bdhn*1(7) *Bdhn*2(4) *Bdhn*3(2) *Bdhn*6(1) *Bdhn*8(2)
					**BS**: *Bdhn*1(8) *Bdhn*2(4) *Bdhn*3(2) *Bdhn*6(1) *Bdhn*8(2)
					**BSY**: *Bdhn*1a(4) *Bdhn*1b(4) *Bdhn*2(4) *Bdhn*3(2) *Bdhn*6(1) *Bdhn*7(2)
C2H2 zinc finger	ZAT	0.738	4.91	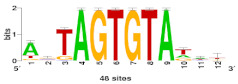	**BD**: *Bdhn*4(3) *Bdhn*5(1) *Bdhn*10(2)
					**BHD**: *Bdhn*5(1)
					**BHS**: *Bdhn*5(1) *Bdhn*10(1)
					**BS**: *Bdhn*5(2) *Bdhn*10(1)

## Data Availability

The complete protocol, input and output data, and Supplementary information are available at Github (https://github.com/Bioflora/Brachypodium_dehydrins (accessed on 28 October 2021)).

## Supplementary Materials

The following are available online at https://www.mdpi.com/article/10.3390/plants10122664/s1, Figure S1: Inferred three dimensional structure of some *Brachypodium* dehydrins. All species’ dehydrins showed α-helices (1 to 4) whereas only some of them presented β-sheets (*Bdhn*1 two in all species except *B. distachyon*, *Bdhn*2 two to three in *B. sylvaticum*, and *Bdhn*6 and *Bdhn*9 three in *B. hybridum*S and *B. sylvaticum*): (A) *B. distachyon Bdhn*4; (B) *B. hybridumD Bdhn*3; (C) *B. stacei Bdhn*1; (D) *B.hybridumS Bdhn*10; (E): *B. sylvaticum Bdhn*1b, Figure S2: BES1/BZR1 (Basic leucine zipper; green), MYB124 (Myb gene protein; blue), and ZAT (C2H2 zinc finger; red) *cis*-regulatory elements found in 5’-upstream promoter region (–500-to-+200 bp) of the *Brachypodium Bdhn* genes. (a) Distributions of *cis*-motifs per species and reference genomes (BD, *B. distachyon* Bd21; BS, *B. stacei* ABR114; BHD, *B. hybridum* subgenome-D ABR113; BHS, *B. hybridum* subgenome-S ABR113; BSY, *B. sylvaticum* Ain1) and per *Bdhn* gene promoter (*Bdhn*1 to *Bdhn*10). Values indicate the number of each type of *cis*-motif found in the promoter region of each gene (see color codes in the chart). (b, c) Analysis of *cis*-regulatory element discovery with Rsat::plants tools in the 5-upstream promoter region (–500-to-+200 bp) of 47 *Bdhn* genes from four *Brachypodium* species. (b) Maximum k-mer significance values from each analysis are shown for *Bdhn* sequences from each species, (c) Maximum number of sites from each background analysis are shown for *Bdhn* sequences from each species. In each case, another 47 random gene sequences from the respective reference genome were analyzed ten times as controls (grey bars; C1-C10), Figure S3: Chromosomal location of the 10 *Bdhn* genes in 54 *B. distachyon* ecotypes. *Bdhn* genes (clusters) detected in each ecotype were compared against the dehydrins of the reference genome (Bd21 v3) and assigned to the cluster with highest score using global pairwise alignment (Needleman-Wunsch with BLOSUM62). *Bdhn* color codes and the accuracy of the annotations are indicated in the charts, Figure S4: Maximum likelihood *B. distachyon* dehydrin trees obtained from the aligned exon and intron sequences of each independent *Bdhn* gene (*Bdhn*1 to *Bdhn*10). Trees were constructed with IQTREE using the *B. stacei* outgroup sequence to root the tree. Bootstrap support is indicated on branches. Accession codes correspond to those indicated in Table S4, Figure S5: Bidimensional PCA plot of 54 *B. distachyon* ecotypes obtained from 19 climate variables (see Table S6). PC1 and PC2 axes comprise 48.5% and 22.4% of the variance, respectively. Ecotype codes correspond to those indicated in Table S4. Ellipses include ecotypes classified within cold (aquamarine), mesic (green), and warm (red) climate classes according to their PC1 values, Figure S6: Boxplots and Wilcoxon pairwise significance tests of differential gene expression values (normalized transcript per million, TPM) of the four expressed dehydrin genes (*Bdhn*1a, *Bdhn*2, *Bdhn*3, *Bdhn*7) under joint and separately analyzed drought and temperature stress conditions in 32 *B. distachyon* ecotypes. Averaged expression values for C (Cool), H (Hot), W (Watered), and D (Drought) treatments and their combinations (see text). (a) Pairwise comparative tests of combined CD-CW-HD-HW treatments; all dehydrins were significantly differentially expressed in all CD vs. CW and HD vs. HW tests, by contrast they were not significantly different in all CD vs. HD tests and in two CW vs. HW tests (*Bdhn*1a, *Bdhn*2); (b) Pairwise comparative tests of D vs. W treatments; all dehydrins were significantly differentially expressed; (c) Pairwise comparative tests of C vs. H treatments; none of the dehydrins were significantly differentially expressed, Figure S7: Differentially expressed *Bdhn*1a*, Bdhn*2*, Bdhn*3 and *Bdhn*7 dehydrin genes (normalized transcript per million, TPM) across 32 ecotypes of *B. distachyon* under drought (D, red) vs. watered (W, blue) conditions. Different letters in the boxplots indicate significant group differences (Tukey tests), Figure S8: Linear model regression plots of pairwise dehydrine *Bdhn* expression values (normalized transcript per million, TPM) under joint drought and watered conditions, Figure S9: Physical locations of orthologous *Brachypodium distachyon* and *Triticum aestivum* water stress responsive dehydrin genes. Drought-induced wheat dehydrin genes are highlighted in colors. *Brachypodium* chomosomes are drawn at 10x scale with respect to wheat chromosomes, Figure S10: Drought-response phenotypic changes as a function of drought treatment averaged across 32 *B. distachyon* ecotypes [leaf_rwc (relative water content in leaf); leaf_wc (water content in leaf); lma (leaf mass per area); pro (leaf proline content); abvrgd (above ground biomass); blwgrd (below ground biomass); ttlmass (total mass); rmr (root mass ratio); delta13c (carbon isotope, a proxy for lifetime integrated WUE); leafc (leaf carbon content); leafn (leaf nitrogen content); cn (leaf carbon/nitrogen ratio)]. Asterisks above boxes indicate Wilcoxon pairwise significant difference among drought (D, red) and watered (W, blue) conditions (*p*-value < 0.001, ***), Figure S11: Linear model regression plots of dehydrine *Bdhn*1a*, Bdhn*2*, Bdhn*3 and *Bdhn*7 expression values (normalized transcript per million, TPM) and drought-response phenotypic traits changes under total dry (D) and watered (W) conditions, Figure S12: Maximum Likelihood *B. distachyon* nuclear species tree cladogram showing the relationships of 30 ecotypes. Phyloheatmaps of normalized values for different sets of variables: (a) dehydrin (*Bdhn*1, *Bdhn*2, *Bdhn*3, *Bdhn*7) gene expression values under watered (W) and drought (D) conditions; (b) drought-response phenotypic traits (leaf_rwc; leaf_wc; lma; pro; abvrgd; blwgrd; ttlmass; rmr; delta13c; leafc; leafn; cn) values under watered (W) and drought (D) conditions; (c) climate niche PCA1 values. Traits showing significant phylogenetic signal are highlighted with dotted lines (see Table S13a), Table S1: Sampled dehydrin sequences from grass species closely related to *Brachypodium*. The accession code and the protein name correspond to those indicated in Phytozome and Genbank. Asterisks indicate the original names of DHN genes. Outgroup DHN sequences orthologous to the corresponding *Brachypodium* Bdhn genes are based on the analyses developed in this study; crosses indicate orthology information retrieved from Ensembl Plants and Phytozome, Table S2: Molecular traits of *Brachypodium Bdhn* proteins. No. aa, number of aminoacids; Mwt, molecular weight; pI, isolectric point; Instability index; GRAVY, grand hydrophathicity average index. Abbreviations of species and reference genomes: BD, *B. distachyon* Bd21; BHD, *B. hybridum* D-subgenome ABR113; BS, *B. stacei* ABR114; BHS, *B. hybridum* S-subgenome ABR113; BSY, *B. sylvaticum* Ain1, Table S3: Chromosomal location of *Bdhn* genes across the four studied *Brachypodium* species and genomes. Chr, chromosome number (*B. distachyon* Bd21: Bd1–Bd5; *B. hybridum* ABR113 subgenome D: Bd1–Bd5; *B. stacei* ABR114: Bs1–Bs10; *B. hybridum* ABR113 subgenome S: Bs1–Bs10; *B. sylvaticum* Ain-1: Bsy1–Bsy9). The highest density of dehydrin genes were found in the syntenic chromosomes Bd3 and Bd4 (*Bdhn*2, *Bdhn*4, *Bdhn*5, *Bdhn*6, *Bdhn*7, *Bdhn*8), Bs4 (*Bdhn*2, *Bdhn*6, *Bdhn*7), the equivalent *B. hybridum* D and S subgenomic chromosomes (except *Bdhn*4), and Bsy4 (*Bdhn*2, *Bdhn*6, *Bdhn*7, *Bdhn*8). Lenghts and positions correspond to the respective reference genomes, Table S4: Sampling origins of the four studied *Brachypodium* species and of 54 ecotypes of *B. distachyon*. All samples were used in the comparative genomic analysis of the dehydrin genes. Asterisks indicate *B. distachyon* accessions additionally used in the dehydrin expression and drought-response phenotypic traits changes analyses (32 ecotypes). Diamonds indicate accessions additionally used in the phylogenetic signal analysis (30 ecotypes), Table S5: Topological congruence tests between (a) the *B. distachyon* nuclear species tree (Gordon et al. 2017) and (b) the *B. distachyon* plastome tree (Sancho et al. 2018) *versus* the *B. distachyon* dehydrin *Bdhn* tree. Test(s) were performed for significance of likelihood-score differences. KH: Kishino-Hasegawa test using normal approximation, two-tailed test. SH: Shimodaira-Hasegawa test using RELL bootstrap (one-tailed test). AU: Shimodaira Approximately Unbiased test. Values for KH/SH/AU tests are P values for the null hypothesis of no difference between trees. *the null hypothesis is accepted. Number of bootstrap replicates = 1,000,000, Table S6: *Brachypodium distachyon* climate data. (a) Values of 19 current climate parameters retrieved from worldclim for the sampled localities of the studied *B. distachyon* ecotypes. Climate, climatic class of the *B. distachyon* ecotypes classified according to their PCA1 values (cold:> 2.5; mesic: (–2.5)–(2.5); warm: < –2.5; see Figure S5). (b) PCA1 and PCA2, coordinate values of the first and second PCA axes obtained from the climate PC analysis, Table S7: *Brachypodium distachyon* dehydrin expression data. Filtered and normalized transcripts per million (TPM) values of annotated dehydrins. Only four dehydrin genes (*Bdhn*1a, *Bdhn*2, *Bdhn*3, *Bdhn*7) were expressed in leaves of 31-days grown plants. Plants were subjected to drought (W: watered, D: Drought) and temperature (C: Cold, H: Hot) stress conditions (see text). Code indicates the sampling code used in the RNAseq analysis, Table S8: Summary statistics of dehydrin *Bdhn*1a, *Bdhn*2, *Bdhn*3 and *Bdhn*7 gene expressions under dry (D) vs. watered (W) conditions and comparative differential expression (DE) tests in *B. distachyon* ecotypes. (a) Kruskal-Wallis rank tests (D vs. W) for each *Bdhn* gene. (b) Wilcoxon pairwise tests of normalized TPM values across ecotypes, *p*-values were adjusted with the Benjamini–Hochberg (BH) procedure, controlling the false discovery rate, to correct for multiple comparisons; n. s., non significant, *p* ≤ 0.05* significant values are highlighted in bold, Table S9: Linear model (lm) regression analysis for comparative differential gene expressions of dehydrin *Bdhn* genes in the studied *B. distachyon* ecotypes. W (watered) and D (dry) conditions. Significant *p*-values (*p* ≤ 0.001***), Table S10: Comparative analysis of dehydrin genes showing upregulated expression under drought compared to watered conditions in *Brachypodium distachyon* and *Triticum aestivum*. Orthology between the differentially expressed genes in the two species was retrieved through Ensembl Plants using BioMart and Blast searches using orthologies previously established in Galvez et al., (2019) (§), Table S11: Summary statistics of 12 drought-response phenotypic traits [leaf_rwc (relative water content in leaf); leaf_wc (water content in leaf); lma (leaf mass per área); pro (leaf proline content); abvrgd (above ground biomass); blwgrd (below ground biomass); ttlmass (total mass); rmr (root mass ratio); delta13c (carbon isotope, a proxy for lifetime integrated WUE); leafc (leaf carbon content); leafn (leaf nitrogen content); cn (leaf carbon/nitrogen ratio)] in dry (D) vs. watered (W) *Brachypodium distachyon* plants. (a) Kruskall-Wallis rank tests (W vs. D) for each phenotypic trait. (b) comparative pairwise Wilcoxon tests in the studied *B. distachyon* ecotypes; *p*-values were adjusted with the Benjamini–Hochberg (BH) procedure, controlling the false discovery rate, to correct for multiple comparisons; n, number of replicates. n. s., non significant, * *p* ≤ 0.05*; significant values are highlighted in bold, Table S12: Linear model (lm) regression analysis for comparative *Brachypodium distachyon Bdhn* gene expressions and drought-induced phenotypic trait changes under total watered (W) and dry (D) conditions. Significant *p*-values (*p* ≤ 0.05 *; 0.01 **; 0.001 ***), Table S13: Phylogenetic signal of dehydrin gene expressions under watered (W) and dry (D) conditions, drought-induced phenotypic traits changes and climate niche variation assessed in (a) the *B. distachyon* nuclear-SNP tree and (b) the *B. distachyon Bdhn* tree using the *phylosig* option of the *phytools* R package. Blomberg’s K and Pagel’s lambda values close to one indicate phylogenetic signal and values close to zero phylogenetic independence. K, *p*-values based on 1000 randomizations; lambda, *p*-values based on the Likelihood Ratio test. Significant and marginal significant values are highlighted in bold.
