# Supplementary material for: Comparative Genomics, Evolution, and Drought-Induced Expression of Dehydrin Genes in Model Brachypodium Grasses
Source: Plants (Basel). 2021 Dec 3;10(12):2664. doi: 10.3390/plants10122664 (PMC8709310; doi:10.3390/plants10122664)
Supplement: Supplementary file 1 [file plants-10-02664-s001.zip › plants-1459797-SI.pdf]

Supplementary Tables

Supplementary Table S1. Sampled dehydrin sequences from grass species closely related to *Brachypodium*. The accession code and the protein name correspond to those indicated in Phytozome and Genbank. Asterisks indicate the original names of DHN genes. Outgroup DHN sequences orthologous to the corresponding *Brachypodium* Bdhn genes are based on the analyses developed in this study; crosses indicate orthology information retrieved from Ensembl Plants and Phytozome.

Panther familiy ortology	***Bdhn***	***Aegilops tauschii***		***Hordeum vulgare***		***Oryza sativa***		***Sorghum bicolor***		***Triticum aestivum***		***Zea mays***	
		**Accession**	**Name**	**Accession**	**Name**	**Accession**	**Name**	**Accession**	**Name**	**Accession**	**Name**	**Accession**	**Name**
ERD14	***Bdhn 1*** ***Bdhn 2***	AET6Gv20653900	DHNAtau 1	HORVU6Hr1G064620	DHNHvul 8*	LOC_Os02g44870	DHNOsat 1†	Sobic.004G286600	DHNSbic 2*†	TraesCS6A02G253300	DHNTaes 1	GRMZM2G147014	DHNZmays 1†
										TraesCS6D02G234700	DHNTaes 2	GRMZM2G373522	DHNZmays 2†
										TraesCS6B02G273400	DHNTaes 3		
SF23	***Bdhn 3***	AET6Gv20864900	DHNAtau 4	HORVU6Hr1G084010	DHNHvul 7*	LOC_Os11g26570	DHNOsat 2†	Sobic.009G116700	DHNSbic 3†	TraesCS6D02G332900	DHNTaes 4	Zm00001d010094	DHNZmays 3†
		AET6Gv20865700	DHNAtau5	HORVU6Hr1G084070	DHNHvul 3*			Sobic.010G041900	DHNSbic 4†	TraesCS6D02G333200	DHNTaes 5		
		AET6Gv20866400	DHNAtau 6							TraesCS6D02G333300	DHNTaes 6		
		AET6Gv20866000	DHNAtau 7							TraesCS6D02G333600	DHNTaes 7		
										TraesCS6A02G350700	DHNTaes 8		

								TraesCS6B02G383500	DHNTaes 9		
								TraesCS6B02G383600	DHNTaes 10		
								TraesCS6D02G333100	DHNTaes 11		
								TraesCS6A02G350600	DHNTaes 12		
								TraesCS6A02G350800	DHNTaes 13		
SF14	***Bdhn 4 Bdhn 5***	AET5Gv20866800	DHNAtau 2*	HORVU5Hr1G092120	DHNHvul1a*	LOC_Os11g26780	DHNOsat 3a†				
		AET5Gv20866700	DHNAtau 9	HORVU5Hr1G092160	DHNHvul 1b	LOC_Os11g26790	DHNOsat 3b†				
				HORVU5Hr1G092100	DHNHvul 2a*						
				HORVU5Hr1G092150	DHNHvul 2b						
	Bdhn 7 Bdhn 8							TraesCS5A02G424700	DHNTaes 14		
								TraesCS5A02G424800	DHNTaes 15		
								TraesCS5B02G426700	DHNTaes 16		
								TraesCS5B02G426800	DHNTaes 17		
								TraesCS5D02G433200	DHNTaes 18		
								TraesCS5D02G433300	***DHNTaes 19***		
SF19	***Bdhn 6***	AET4Gv20132600	DHNAtau 3*							GRMZM2G052364	DHNZmays 4†

										GRMZM2G098750	DHNZmays 5†
XEROI	***Bdhn 9***	AET3Gv20620600	DHNAtau 8			LOC_Os01g50700	DHNOsat 4	Sobic.003G270200	DHNSbic 5		
HIRD11	***Bdhn 10***			AY681974	DHNHvul 13*	LOC_Os03g45280	DHNOsat 13	Sobic.001G149500	DHNSbic 10	GRMZM2G169372	DHNZmays 10a
										GRMZM2G448511	DHNZmays 10b

Supplementary Table S2. Molecular traits of *Brachypodium Bdhn* proteins. No. aa, number of aminoacids; Mwt, molecular weight; pI, isolectric point; Instability index; GRAVY, grand hydrophathicity average index. Abbreviations of species and reference genomes: BD, *B. distachyon* Bd21; BHD, *B. hybridum* D-subgenome ABR113; BS, *B. stacei* ABR114; BHS, *B. hybridum* S-subgenome ABR113; BSY, *B. sylvaticum* Ain1.

Bdhn	**No. aa**					**Mwt (Kda)**					**pI**					**Instability index**					**GRAVY**				
	BD	BHD	BS	BHS	BSY	BD	BHD	BS	BHS	BSY	BD	BHD	BS	BHS	BSY	BD	BHD	BS	BHS	BSY	BD	BHD	BS	BHS	BSY
***Bdhn*1a**	252	252	259	259	252	27506.5	27575.6	28096.2	28096.2	27255.3	5.24	5.30	5.54	5.54	5.30	58.09	60.80	58.20	58.20	61.23	-1.17	-1.18	-1.16	-1.16	-1.09
***Bdhn*1b**					252					27354.4					5.42					55.47					-1.11
***Bdhn*2**	254	254	252	252	258	27504.6	27490.6	27304.4	27352.5	28032.2	5.24	5.24	5.30	5.30	5.37	64.07	63.54	65.46	64.41	61.02	-1.04	-1.04	-1.10	-1.12	-1.13
***Bdhn*3**	163	163	160	160	162	16348.6	16348.6	16153.4	15978.2	16107.3	9.13	9.13	7.17	6.79	8.87	15.43	15.43	17.31	18.55	14.94	-1.07	-1.07	-1.03	-1.00	-1.03
***Bdhn*4**	107					11015.2					9.40					47.63					-1.24				
***Bdhn*5**	143	143	132	132	145	14468.8	14469.8	13518.8	13504.8	14730.0	8.86	8.86	9.16	9.16	8.07	28.95	28.95	30.08	29.44	32.68	-1.05	-1.05	-1.08	-1.08	-1.06
***Bdhn*6**	395	395	362	363	423	37817.7	37751.5	34828.3	35013.6	40262.1	9.03	9.05	8.53	8.55	8.06	8.01	7.75	12.31	9.67	7.23	-0.67	-0.69	-0.76	-0.76	-0.67
***Bdhn*7**	183	183	181	181	180	18455.9	18429.9	18340.8	18340.8	18203.7	9.25	9.25	9.05	9.05	9.25	18.26	17.79	21.10	21.10	16.35	-1.10	-1.08	-1.14	-1.14	-1.03
***Bdhn*8**	157	157	169	169	172	16125.5	16125.5	17248.6	17226.6	17528.0	9.07	9.07	9.05	9.05	9.33	21.05	21.05	22.31	22.76	29.13	-1.15	-1.15	-1.12	-1.12	-1.12
***Bdhn*9**	226	226	206	206	222	23501.6	23532.6	21570.5	21570.5	22965.1	5.79	5.68	5.99	5.99	6.06	43.84	41.28	42.75	42.75	41.40	-0.80	-0.83	-0.94	-0.94	-0.83
***Bdhn*10**	100	102	107	107	102	11313.5	11515.6	12109.3	12109.3	11529.7	7.23	6.87	7.25	7.25	6.87	41.87	43.14	42.39	42.39	43.14	-2.15	-2.15	-2.19	-2.19	-2.15

Supplementary Table S3. Chromosomal location of *Bdhn* genes across the four studied *Brachypodium* species and genomes. Chr, chromosome number (*B. distachyon* Bd21: Bd1-Bd5; *B. hybridum* ABR113 subgenome D: Bd1-Bd5; *B. stacei* ABR114: Bs1-Bs10; *B. hybridum* ABR113 subgenome S: Bs1-Bs10; *B. sylvaticum* Ain-1: Bsy1-Bsy9). The highest density of dehydrin genes were found in the syntenic chromosomes Bd3 and Bd4 (*Bdhn*2, *Bdhn*4, *Bdhn*5, *Bdhn*6, *Bdhn*7, *Bdhn*8), Bs4 (*Bdhn*2, *Bdhn*6, *Bdhn*7), the equivalent *B. hybridum* D and S subgenomic chromosomes (except *Bdhn*4), and Bsy4 (*Bdhn*2, *Bdhn*6, *Bdhn*7, *Bdhn*8). Lenghts and positions correspond to the respective reference genomes.

		***Bdhn*1a**	***Bdhn*1b**	***Bdhn*2**	***Bdhn*3**	***Bdhn*4**	***Bdhn*5**	***Bdhn*6**	***Bdhn*7**	***Bdhn*8**	***Bdhn*9**	***Bdhn*10**
B.distachyon	**Chr**	Bd5		Bd3	Bd1	Bd 4	Bd 4	Bd 4	Bd 3	Bd 3	Bd 2	Bd 1
Bd21	**Lenght**	28630136		59640145	75071545	48594894	48594894	48594894	59640145	59640145	59130575	75071545
	**from**	14358126		52061272	33400971	26400080	26401854	22188216	45300502	45290875	47751383	10098645
	**to**	14359531		52062769	33402000	26400807	26402677	22189942	45301669	45291925	47757368	10100931
	**direction**	forward		forward	reverse	reverse	reverse	forward	reverse	forward	forward	reverse
***B.hybridum*D**	**Chr**	Bd5		Bd3	Bd1		Bd 4	Bd 4	Bd3	Bd3	Bd 2	Bd 1
ABR113	**Lenght**	28673805		59422649	73190849		48381198	48381198	59422649	59422649	59597844	73190849
	**from**	14493652		51909664	32658384		25616401	21335117	45017036	45007503	47969138	9743153
	**to**	14494867		51910828	32659379		25617226	21336708	45018083	45008367	47975373	9743841
	**direction**	forward		forward	reverse		reverse	forward	reverse	forward	forward	reverse
B. stacei	**Chr**	Bs9		Bs4	Bs7		Bs5	Bs5	Bs4	Bs4	Bs1	Bs2
ABR114	**Lenght**	20576529		24645555	20893312		23048618	23048618	24645555	24645555	30086066	27792411
	**from**	9210852		6997868	14026099		3806913	6372578	13156720	13537514	10860306	18652003
	**to**	9212190		6999029	14026708		3849990	6374159	13157693	13538422	10866472	18652813
	**direction**	forward		reverse	forward		forward	reverse	forward	forward	reverse	forward
***B. hybridum*S**	**Chr**	Bs9		Bs4	Bs7		Bs5	Bs5	Bs4	Bs4	Bs1	Bs2
ABR113	**Lenght**	21007308		25447193	21638549		23591727	23591727	25447193	25447193	30608744	28346489
	**from**	7214663		7269373	14706419		3849103	7640493	13819476	13842937	10890765	19034663

	**to**	7216004		7270541	14707427		3849990	7642071	13820451	13843854	10897704	19035490
	**direction**	forward		reverse	forward		forward	forward	forward	reverse	reverse	forward
B. sylvaticum	**Chr**	Bsy9	Bsy9	Bsy4	Bsy7		Bsy5	Bsy4	Bsy4	Bsy4	Bsy1	Bsy2
Ain-1	**Lenght**	31923712	31923712	38747968	22318590		48035605	38747968	38747968	38747968	52666873	42817455
	**from**	13763612	14190187	9798501	14062257		26543391	21623366	19478535	19507464	17853540	25255158
	**to**	13765006	14191050	9799643	14062972		26543956	21625489	19479154	19508169	17853540	25256158
	**direction**	forward	reverse	reverse	forward		reverse	forward	forward	reverse	reverse	forward

Supplementary Table S4. Sampling origins of the four studied *Brachypodium* species and of 54 ecotypes of *B. distachyon*. All samples were used in the comparative genomic analysis of the dehydrin genes. Asterisks indicate *B. distachyon* accessions additionally used in the dehydrin expression and drought-response phenotypic traits changes analyses (32 ecotypes). Diamonds indicate accessions additionally used in the phylogenetic signal analysis (30 ecotypes).

Species	accession	longitude	latitude	Locality
B.stacei	ABR114	38.682846	1.398957	Spain: Balearic isles, Formentera, Torrent
B.hybridum	ABR113	38.782993	-9.250488	Portugal: Lisboa, Belas
B.sylvaticum	Ain1	36.768235	8.707878	Tunisia: Ain-Draham
B.distachyon	ABR2*♦	3,3000	43,6500	France: Herault, Octon
B.distachyon	ABR3*♦	0,0731	42,1805	Spain: Huesca, Aisa
B.distachyon	ABR4*♦	0,7168	42,2627	Spain: Huesca, Aren
B.distachyon	ABR5*♦	-0,5800	42,5810	Spain: Huesca, Jaca, Banaguas
B.distachyon	ABR6*♦	-2,2030	42,5810	Spain: Navarra, Los Arcos
B.distachyon	ABR8*♦	11,3197	43,3146	Italy: Siena
B.distachyon	ABR9	14,4895	46,0609	Croatia: Ljubjana
B.distachyon	Adi10*♦	38,3523	38,7707	Turkey: Adiyaman
B.distachyon	Adi12*♦	38,3523	38,7707	Turkey: Adiyaman
B.distachyon	Adi2*♦	38,3523	38,7707	Turkey: Adiyaman
B.distachyon	Arn1	0,7299	42,2565	Spain: Huesca, Arén
B.distachyon	Bd1_1*♦	28,2510	38,4170	Turkey: Manisa
B.distachyon	Bd18_1*♦	33,7300	39,3678	Turkey: Kaman
B.distachyon	Bd2_3*♦	44,4031	33,7609	Irak: Al Mansuriya
B.distachyon	Bd21_3*♦	44,5350	36,7660	Irak: near Salakudin
B.distachyon	Bd29_1	33,5639	44,5153	Ukraine: Krimea
B.distachyon	Bd3_1*♦	44,5350	36,7660	Irak: Al Mansuriya
B.distachyon	BdTR10C*♦	31,8849	37,7782	Turkey: Konya Province
B.distachyon	BdTR11A	31,8849	37,7782	Turkey: Konya Province
B.distachyon	BdTR11G*♦	27,4770	41,4220	Turkey: Kirklareli
B.distachyon	BdTR11I*♦	28,0402	39,7382	Turkey: Balikesir, Karakaya
B.distachyon	BdTR12C*	34,6503	39,7482	Turkey: Saray, Yozgat province
B.distachyon	BdTR13a*♦	32,4324	39,7565	Turkey: Ankara
B.distachyon	BdTR13C*	32,9881	39,4129	Turkey: Ankara
B.distachyon	BdTR1i*♦	28,5830	38,0930	Turkey: Aydin
B.distachyon	BdTR2B*♦	31,3311	40,0821	Turkey: Karahisarkozlu
B.distachyon	BdTR2G*♦	32,9850	40,3940	Turkey: Ankara
B.distachyon	BdTR3C*♦	32,9630	36,7830	Turkey: Balkusan
B.distachyon	BdTR5i*♦	32,9854	40,3936	Turkey: Cubuk
B.distachyon	BdTR7A	34,6500	39,7480	Turkey: Yozgat

B.distachyon	BdTR8i	34,0714	37,1885	Turkey: Berendi
B.distachyon	BdTR9K*♦	30,7886	39,7530	Turkey: Eskisehir
B.distachyon	Bis1*♦	41,0151	37,8735	Turkey: Bismil
B.distachyon	Foz1	-1,3050	42,6370	Spain: Navarra, Foz de Lumbier
B.distachyon	Gaz8	37,3910	37,1280	Turkey: Gaziantep
B.distachyon	Jer1	0,0120	42,0550	Spain: Huesca, Adahuesca
B.distachyon	Kah1*♦	38,5330	37,7340	Turkey: Kahta
B.distachyon	Kah5*♦	38,5330	37,7340	Turkey: Kahta
B.distachyon	Koz1*♦	41,6100	38,1520	Turkey: Kozluk
B.distachyon	Koz3*♦	41,6100	38,1520	Turkey: Kozluk
B.distachyon	Luc1	-0,8930	42,6100	Spain: Huesca, Berdun
B.distachyon	Mig3	-0,2050	42,1470	Spain: Huesca, Ibieca, San Miguel de Foces
B.distachyon	Mon3	-0,2090	41,6520	Spain: Zaragoza, Castejón de Monegros
B.distachyon	Mur1	0,8770	42,0980	Spain: Lleida, Castillo de Mur
B.distachyon	Per1	-1,7500	42,7370	Spain: Navarra, Puerto del Perdon
B.distachyon	S8iiC	0,1440	41,6054	Spain: Huesca, Zaidín
B.distachyon	Sig2	-1,0150	42,6130	Spain: Zaragoza, Sigüés
B.distachyon	Tek2	26,9310	41,0850	Turkey: Tekirdag
B.distachyon	Tek4	27,5191	41,0112	Turkey: Tekirdag

Supplementary Table S5. Topological congruence tests between **(a)** the *B. distachyon* nuclear species tree (Gordon et al. 2017) and **(b)** the *B. distachyon* plastome tree (Sancho et al. 2018) *versus* the *B. distachyon* dehydrin *Bdhn* tree. Test(s) were performed for significance of likelihood-score differences. KH: Kishino-Hasegawa test using normal approximation, two-tailed test**.** SH: Shimodaira-Hasegawa test using RELL bootstrap (one-tailed test). AU: Shimodaira Approximately Unbiased test**.** Values for KH/SH/AU tests are P values for the null hypothesis of no difference between trees**.** *the null hypothesis is accepted. Number of bootstrap replicates = 1,000,000.

	KH test					SH		
a)	**-lnL**	**Diff'-lnL**	**s.d.**	**T**	**P**	**SH-test**	**wtd-SH**	**AU**
**Tree1= Brachy nuclear tree (best)**	46255.5731	(best)						
**Tree2= Brachy *Bdhn* tree**	54391.9775	8136.40442	202.241	40.231	<0.0001*	0.0000*	0.0000*	~0*
**Tree1=Brachy *Bdhn* tree (best)**	5898.89581	(best)						
**Tree2=Brachy nuclear tree**	6170.77777	271.88196	59.738	4.551	<0.0001*	0.0000*	0.0000*	~0*
	* P < 0.05							
(b)								
**Tree1= Brachy plastome tree (best)**	1442.4868	(best)						
**Tree2= Brachy *Bdhn* tree**	2334.75366	892.26686	52.856	16.881	<0.0001*	0.0000*	0.0000*	~0*
**Tree1= Brachy *Bdhn* tree (best)**	5898.89581	(best)						
**Tree2= Brachy plastome tree**	6268.97012	370.07431	91.549	4.042	0.0001*	0.0003*	0.0003*	~0*
	* P < 0.05							

Supplementary Table S6. *Brachypodium distachyon* climate data. **(a)** Values of 19 current climate parameters retrieved from worldclim for the sampled localities of the studied *B. distachyon* ecotypes. Climate, climatic class of the *B. distachyon* ecotypes classified according to their PCA1 values (cold:> 2.5; mesic: (-2.5) – (2.5); warm: < -2.5; see Supplementary Figure S5). **(b)** PCA1 and PCA2, coordinate values of the first and second PCA axes obtained from the climate PC analysis.

(a)

ecotype	longitude	latitude	altitude	bio1	bio2	bio3	bio4	bio5	bio6	bio7	bio8	bio9	bio10	bio11	bio12	bio13	bio14	bio15	bio16	bio17	bio18	bio19	PCA1	Climate
ABR2	3.3	43.65	265	13.3	10	3.7	577.4	27.9	1.3	26.6	9.9	20.8	20.8	6	707	85	29	23	218	126	126	188	2.5793	Cold
ABR3	0.07311	42.1805	798	10.1	10	3.8	566.4	24.6	-1.5	26.1	12.1	3	17.5	3	688	79	40	18	206	143	162	143	4.8611	Cold
ABR4	0.7168	42.26265	932	9.9	9.5	3.6	582.3	24.2	-1.6	25.8	11.8	2.6	17.4	2.6	873	96	53	18	262	176	227	176	5.6901	Cold
ABR5	-0.58	42.581	986	8.7	9.8	3.9	540.9	22.6	-2.4	25	10.5	15.8	15.8	2	842	90	48	15	237	177	184	208	5.3953	Cold
ABR6	-2.203	42.581	557	12.1	10.1	3.9	557.9	26.6	0.9	25.7	8.8	19.3	19.3	5.1	668	71	36	18	191	131	131	174	3.4004	Cold
ABR8	11.319695	43.314569	300	13.7	9	3.4	600.3	28.7	2.5	26.2	10.5	21.6	21.6	6.4	757	101	29	29	262	117	117	203	2.1759	Mesic
Adi10	38.352277	38.770694	839	13.6	9.8	2.7	936.7	33.4	-2.8	36.2	12.2	25.3	25.3	1.3	459	63	2	57	172	13	25	161	-1.7567	Mesic
Adi12	38.352277	38.770694	839	13.6	9.8	2.7	936.7	33.4	-2.8	36.2	12.2	25.3	25.3	1.3	459	63	2	57	172	13	25	161	-1.7567	Mesic
Adi2	38.352277	38.770694	839	13.6	9.8	2.7	936.7	33.4	-2.8	36.2	12.2	25.3	25.3	1.3	459	63	2	57	172	13	25	161	-1.7567	Mesic
Bd1-1	28.251	38.417	644	13.8	11.5	3.8	660.1	30.2	0.7	29.5	5.6	22	22.4	5.6	698	146	8	72	360	38	39	360	-0.5973	Mesic
Bd18-1	33.730025	39.36784	1057	10.4	10.9	3.4	747.4	27.4	-4.3	31.7	0.4	19.4	19.6	0.4	445	63	6	48	161	29	49	161	0.7309	Mesic
Bd2-3	44.403075	33.760883	40	22.7	15.2	3.8	877.5	43.7	4.5	39.2	12.7	33.7	33.7	11.3	171	31	0	86	88	0	0	87	-5.7177	Warm
Bd21ctrl	44.535	36.766	1089	15	12.1	3.1	961.1	36	-2.8	38.8	3.6	27	27	2.6	728	136	0	86	385	3	3	349	-3.5166	Warm
Bd21-3	44.535	36.766	1089	15	12.1	3.1	961.1	36	-2.8	38.8	3.6	27	27	2.6	728	136	0	86	385	3	3	349	-3.5166	Warm
Bd3-1	44.535	36.766	1089	15	12.1	3.1	961.1	36	-2.8	38.8	3.6	27	27	2.6	728	136	0	86	385	3	3	349	-3.5166	Warm
Bd30-1	-3.558733	36.990489	810	14.7	11.4	3.8	603.2	31.7	2.2	29.5	8.4	22.9	22.9	7.5	467	63	5	54	181	24	24	175	-0.4576	Mesic
BdTR10c	31.884911	37.778233	1448	9.4	11.5	3.5	750	27.2	-5.6	32.8	-0.3	18.6	18.9	-0.3	522	77	10	49	209	36	52	209	0.8878	Mesic
BdTR11g	27.477	41.422	89	13	11.3	3.7	666.2	29.5	-0.3	29.8	6.2	20.9	21.5	4.5	598	78	18	37	227	74	86	202	1.0333	Mesic
BdTR11i	28.040197	39.738164	229	13.7	10.9	3.7	683.7	29.8	0.5	29.3	7	21.9	22.4	5	652	108	11	56	284	48	49	282	0.0435	Mesic
BdTR1i	28.583	38.093	956	12.6	11.5	3.7	697.5	29.7	-0.9	30.6	3.9	21.3	21.6	3.9	748	146	10	68	376	43	49	376	-0.2852	Mesic
BdTR2b	31.331114	40.082097	894	10.9	10.5	3.4	714.2	27.4	-2.9	30.3	1.5	19.6	19.8	1.5	488	61	17	34	159	60	85	159	1.5604	Mesic
BdTR2g	32.985	40.394	1531	7.3	10.5	3.3	722	23.9	-7.1	31	-2.4	16	16.2	-2.4	623	84	20	42	221	67	97	221	2.6038	Cold
BdTR5i	32.985367	40.393647	1531	7.3	10.5	3.3	722	23.9	-7.1	31	-2.4	16	16.2	-2.4	623	84	20	42	221	67	97	221	2.6038	Cold
BdTR9k	30.788631	39.75295	900	10.7	11	3.5	729.1	27.8	-3.5	31.3	9.8	19.5	19.8	1.1	419	52	11	37	136	45	67	134	1.1200	Mesic
Bis1	41.015083	37.876556	608	16.5	13.1	3.3	928.6	38.9	-0.6	39.5	9.7	28.3	28.3	4.5	548	83	1	70	237	6	10	219	-3.6626	Warm
Kah1	38.533	37.734	657	16.9	11	2.9	914.8	37.3	0.6	36.7	5.3	28.6	28.6	5.3	586	108	1	77	291	7	10	291	-3.5423	Warm
Kah5	38.533	37.734	657	16.9	11	2.9	914.8	37.3	0.6	36.7	5.3	28.6	28.6	5.3	586	108	1	77	291	7	10	291	-3.5423	Warm
Koz1	41.61	38.152	819	15.3	12.1	3	951.9	37.2	-2.1	39.3	8.3	27.4	27.4	3.1	703	104	1	69	303	10	10	283	-3.2693	Warm
Koz3	41.61	38.152	819	15.3	12.1	3	951.9	37.2	-2.1	39.3	8.3	27.4	27.4	3.1	703	104	1	69	303	10	10	283	-3.2693	Warm
RON2	-0.963	42.781	956	8.9	9.9	3.9	539.5	22.9	-2.2	25.1	3.1	16	16	2.2	952	101	52	16	274	190	190	257	5.4782	Cold

(b)

Variable contribution	PCA1	PCA2
bio1	7.04157791	2.8336316
bio2	5.1431788	0.23831544
bio3	3.59658313	1.20932109
bio4	7.92629573	0.71530785
bio5	9.29173338	0.57639096
bio6	0.48821208	6.97369089
bio7	8.7044993	0.30756729
bio8	0.03627953	10.374377
bio9	8.12122146	0.14333639
bio10	9.05581757	0.73901616
bio11	1.08408395	6.54349079
bio12	2.28358537	11.4483415
bio13	0.23276403	18.6365656
bio14	8.64758564	0.05494295
bio15	9.03019188	1.48194295
bio16	0.49621767	18.6220566
bio17	8.61189114	0.08756497
bio18	9.14009127	0.07915297
bio19	1.06819015	18.9349869

Supplementary Table S7. *Brachypodium distachyon* dehydrin expression data. Filtered and normalized transcripts per million (TPM) values of annotated dehydrins. Only four dehydrin genes (*Bdhn*1a, *Bdhn*2, *Bdhn*3, *Bdhn*7) were expressed in leaves of 31-days grown plants. Plants were subjected to drought (W: watered, D: Drought) and temperature (C: Cold, H: Hot) stress conditions (see text). Code indicates the sampling code used in the RNAseq analysis.

	Drougth (D)						Watered (W)				
**Code**	**accession**	***Bdhn*1a**	***Bdhn*2**	***Bdhn*3**	***Bdhn*7**	**Code**	**accession**	***Bdhn*1a**	***Bdhn*2**	***Bdhn*3**	***Bdhn*7**
BA030_HD_ABR2	ABR2	309,6	207,4	131,8	21	BA053_HW_ABR2	ABR2	199,6	27,2	5,3	1
BA101_HD_ABR2	ABR2	150,4	142,4	278,7	19	BA085_HW_ABR2	ABR2	143,6	67,5	39,1	3,6
BA366_CD_ABR2	ABR2	181,8	183,1	227,7	26,6	BA145_HW_ABR2	ABR2	132,7	36,5	0	1,7
BA439_CD_ABR2	ABR2	214,9	135,5	51,5	11	BA403_CW_ABR2	ABR2	163,6	53	30,2	2,9
BA006_HD_ABR3	ABR3	304,9	182,2	32,1	10,7	BA447_CW_ABR2	ABR2	120,5	52	26	4,7
BA103_HD_ABR3	ABR3	197,7	326	738,2	75,7	BA096_HW_ABR3	ABR3	248,9	29,4	8,5	3,8
BA418_CD_ABR3	ABR3	328,3	116,6	43,9	9,4	BA146_HW_ABR3	ABR3	132,4	13,2	3,3	0
BA465_CD_ABR3	ABR3	224,2	51,4	65,3	22,3	BA413_CW_ABR3	ABR3	193,5	46,6	26,9	1,8
BA038_HD_ABR4	ABR4	299,5	76,8	7,3	2,4	BA419_CW_ABR3	ABR3	205,1	69,4	10,1	1,3
BA170_HD_ABR4	ABR4	226,2	194,7	164,3	37,6	BA040_HW_ABR4	ABR4	109,5	27,1	1,2	0
BA368_CD_ABR4	ABR4	270,3	65,9	15,7	15,7	BA043_HW_ABR4	ABR4	230,1	35,3	7,3	3,7
BA521_CD_ABR4	ABR4	192,6	96,3	61,4	4,7	BA477_CW_ABR4	ABR4	594,4	80,7	5,8	2,7
BA024_HD_ABR5	ABR5	335,1	298,2	62,4	17	BA508_CW_ABR4	ABR4	143,5	50,5	5,8	3,2
BA104_HD_ABR5	ABR5	234,1	580,1	681,7	92,8	BA161_HW_ABR5	ABR5	143,9	33,7	7,6	2,4
BA454_CD_ABR5	ABR5	188,7	55,4	140,2	45	BA479_CW_ABR5	ABR5	418	55,8	3,9	0,4
BA522_CD_ABR5	ABR5	217,7	279,5	46,2	21,9	BA502_CW_ABR5	ABR5	254,8	23,3	9,4	6,1
BA037_HD_ABR6	ABR6	333,5	130,4	147,8	13,5	BA123_HW_ABR6	ABR6	357,3	39,7	3,6	10,8
BA099_HD_ABR6	ABR6	281,5	397,3	2175,5	102,5	BA153_HW_ABR6	ABR6	340,4	26,1	3,9	3,9
BA416_CD_ABR6	ABR6	289	199,9	778,6	33,6	BA437_CW_ABR6	ABR6	258,1	26,1	4,4	2,5
BA523_CD_ABR6	ABR6	256,7	90,5	300,3	16,6	BA452_CW_ABR6	ABR6	308,2	18,1	3,1	2,6
BA100_HD_ABR8	ABR8	298,2	144,7	199,2	63,3	BA008_HW_ABR8	ABR8	224,6	36,4	4,2	0
BA143_HD_ABR8	ABR8	379,6	336,2	262,1	112	BA138_HW_ABR8	ABR8	128,8	21,1	3,5	0
BA415_CD_ABR8	ABR8	502,2	312,7	177,6	84,1	BA360_CW_ABR8	ABR8	249	35,8	5,6	1,7
BA506_CD_ABR8	ABR8	940,2	644,1	285,3	251,7	BA517_CW_ABR8	ABR8	292,4	23,1	3,3	2,3
BA049_HD_Adi10	Adi10	440	197,7	215,9	112,4	BA067_HW_Adi10	Adi10	632,4	37,3	1,7	0,4
BA052_HD_Adi10	Adi10	357,1	249,5	258,2	64,7	BA105_HW_Adi10	Adi10	415,9	49,3	8,4	3,1
BA478_CD_Adi10	Adi10	820,4	1251,9	1961,7	462	BA428_CW_Adi10	Adi10	841,2	51	8,7	6,5
BA513_CD_Adi10	Adi10	1020,1	2043,2	2431,9	692,5	BA520_CW_Adi10	Adi10	341,2	20,1	8,1	2,9
BA036_HD_Adi12	Adi12	558	751,2	290,8	59,5	BA041_HW_Adi12	Adi12	339	81,9	4,9	2,4
BA140_HD_Adi12	Adi12	377,1	260,2	133	56,5	BA044_HW_Adi12	Adi12	396,1	43,2	0	0
BA455_CD_Adi12	Adi12	778,7	840	1104,1	330	BA407_CW_Adi12	Adi12	193,2	96	30,9	13,7
BA525_CD_Adi12	Adi12	240,8	515,5	466,2	113,3	BA423_CW_Adi12	Adi12	746,4	53,9	11,2	1,7

BA050_HD_Adi2	Adi2	458,9	161,7	37,1	11
BA094_HD_Adi2	Adi2	396,1	457,9	318,7	55,9
BA500_CD_Adi2	Adi2	528,3	913,4	957	123,8
BA509_CD_Adi2	Adi2	456,6	405,1	463,1	64,5
BA025_HD_Bd1-1	Bd1-1	475,8	282,7	88,6	27,7
BA051_HD_Bd1-1	Bd1-1	352,8	22	3,3	2,2
BA122_HD_Bd1-1	Bd1-1	235,1	42,5	53,7	4,5
BA442_CD_Bd1-1	Bd1-1	369,3	70,6	42	14,3
BA475_CD_Bd1-1	Bd1-1	582,4	352,4	578,5	79,5
BA093_HD_Bd18-1	Bd18-1	464,3	392	257,2	138,3
BA453_CD_Bd18-1	Bd18-1	1009,6	704,2	564	207,5
BA056_HD_Bd21	Bd21	292,8	125	23,5	12,3
BA163_HD_Bd21	Bd21	249,1	129,2	28,3	45,6
BA459_CD_Bd21	Bd21	440,2	148,4	65,8	36,5
BA499_CD_Bd21	Bd21	325,3	52,3	4,7	2,9
BA097_HD_Bd21-3	Bd21-3	478,7	621,3	567	243,4
BA111_HD_Bd21-3	Bd21-3	452,8	647	487,3	145,7
BA430_CD_Bd21-3	Bd21-3	966,8	1402	1464,4	692,9
BA512_CD_Bd21-3	Bd21-3	503,2	539,2	266,5	103,9
BA023_HD_Bd2-3	Bd2-3	531,4	253	121,1	16,7
BA088_HD_Bd2-3	Bd2-3	635,6	173,8	50,8	38,3
BA353_CD_Bd2-3	Bd2-3	291,6	40,1	6,9	1,9
BA494_CD_Bd2-3	Bd2-3	550,1	341,3	200,5	60,2
BA079_HD_Bd30-1	Bd30-1	353,5	492,1	287	76,5
BA162_HD_Bd30-1	Bd30-1	198,6	406,5	439,7	73,3
BA425_CD_Bd30-1	Bd30-1	1005,9	1632,6	2616,2	685,5
BA481_CD_Bd30-1	Bd30-1	396,7	515,6	283,3	55,5
BA166_HD_Bd3-1	Bd3-1	316,4	405,3	1017,8	55,9
BA398_CD_Bd3-1	Bd3-1	441,6	446,3	730,1	78
BA422_CD_Bd3-1	Bd3-1	427,5	544,5	3386,7	854,4
BA121_HD_BdTR10c	BdTR10c	242,1	214,3	471,6	66,8
BA173_HD_BdTR10c	BdTR10c	380,8	122,7	284,2	82
BA421_CD_BdTR10c	BdTR10c	603,2	483	1912,7	439,9
BA440_CD_BdTR10c	BdTR10c	415,4	490,6	1028,6	187,7
BA059_HD_BdTR11g	BdTR11g	452,4	370,4	615,1	63,5
BA090_HD_BdTR11g	BdTR11g	590,1	645,2	1214,5	215,9
BA397_CD_BdTR11g	BdTR11g	479,7	522,4	607	65,4

BA176_HW_Adi2	Adi2	402,2	68	5	3,8
BA357_CW_Adi2	Adi2	489,2	36,5	1,1	3,2
BA468_CW_Adi2	Adi2	398,6	65,7	4	2
BA354_CW_Bd1-1	Bd1-1	390,9	46,9	8,1	1,5
BA496_CW_Bd1-1	Bd1-1	320	17,6	14,4	4
BA060_HW_Bd18-1	Bd18-1	239,9	21,8	5	0
BA063_HW_Bd18-1	Bd18-1	278,7	16,2	2,8	0,9
BA375_CW_Bd18-1	Bd18-1	160,7	14,5	6,9	0,8
BA446_CW_Bd18-1	Bd18-1	266,7	14,6	6,3	0
BA069_HW_Bd21	Bd21	324,2	24,3	1,3	0,7
BA112_HW_Bd21	Bd21	363,1	70	24	5,7
BA386_CW_Bd21	Bd21	183,1	24,9	2,4	1
BA456_CW_Bd21	Bd21	330	59,6	37,8	6
BA054_HW_Bd21-3	Bd21-3	307,6	25,8	9,4	1,8
BA171_HW_Bd21-3	Bd21-3	276,1	26,2	18,8	0,7
BA458_CW_Bd21-3	Bd21-3	288,6	25,4	14,7	2
BA086_HW_Bd2-3	Bd2-3	181,2	37,4	5	2,1
BA114_HW_Bd2-3	Bd2-3	422,7	54,3	0	0
BA487_CW_Bd2-3	Bd2-3	327	82,5	6,9	3
BA492_CW_Bd2-3	Bd2-3	542,5	48,5	3,8	4,9
BA005_HW_Bd30-1	Bd30-1	266,2	37,1	5	3
BA018_HW_Bd30-1	Bd30-1	244,7	20,3	2,5	0,4
BA417_CW_Bd30-1	Bd30-1	504,7	32,9	8,1	2,9
BA474_CW_Bd30-1	Bd30-1	444,6	44	7,3	2,9
BA007_HW_Bd3-1	Bd3-1	321,4	34,7	4,6	1,5
BA012_HW_Bd3-1	Bd3-1	287,1	46,4	2,6	2,2
BA429_CW_Bd3-1	Bd3-1	477,4	43	20,9	0
BA434_CW_Bd3-1	Bd3-1	256,1	46,6	10	7,2
BA119_HW_BdTR10c	BdTR10c	196,7	36,9	0	0
BA131_HW_BdTR10c	BdTR10c	232	65	25,5	0
BA384_CW_BdTR10c	BdTR10c	223	29,9	1,3	0
BA436_CW_BdTR10c	BdTR10c	177,4	56,1	7,1	3,5
BA021_HW_BdTR11g	BdTR11g	320,5	63	6,2	1,4
BA032_HW_BdTR11g	BdTR11g	547	46,8	9,1	2,9
BA406_CW_BdTR11g	BdTR11g	373,6	78,3	51,5	22,4
BA493_CW_BdTR11g	BdTR11g	314,6	49,3	3,6	2,1
BA042_HW_BdTR11i	BdTR11i	274,4	30,1	2,8	2,1

BA510_CD_BdTR11g	BdTR11g	466,4	280,8	135,7	37,8
BA102_HD_BdTR11i	BdTR11i	483,9	567,1	981,6	139,8
BA128_HD_BdTR11i	BdTR11i	505,6	651	1554,8	175,1
BA408_CD_BdTR11i	BdTR11i	708,1	680,3	833,4	135,7
BA450_CD_BdTR11i	BdTR11i	429,9	314,4	305,8	46,7
BA361_CD_BdtR12c	BdtR12c	297,8	37	9,7	5,8
BA061_HD_BdTR13a	BdTR13a	382	213,3	110,5	41,8
BA144_HD_BdTR13a	BdTR13a	369,6	249,2	130,1	69,2
BA394_CD_BdTR13a	BdTR13a	363,5	160	95,3	31,8
BA460_CD_BdTR13a	BdTR13a	1347,2	930,2	735,4	270,3
BA108_HD_BdTR1i	BdTR1i	639,7	788	1133,3	488,1
BA134_HD_BdTR1i	BdTR1i	315,7	191,5	199,9	73,1
BA457_CD_BdTR1i	BdTR1i	579,7	591,8	246,9	79
BA480_CD_BdTR1i	BdTR1i	796,6	989,7	1329,2	364,4
BA003_HD_BdTR2b	BdTR2b	874,2	662,1	896,5	314,5
BA091_HD_BdTR2b	BdTR2b	658,5	524,5	502,4	140
BA389_CD_BdTR2b	BdTR2b	299,5	213,3	177,2	58,6
BA472_CD_BdTR2b	BdTR2b	320,5	222,3	168,1	32,5
BA124_HD_BdTR2g	BdTR2g	784,1	1119	2009,5	616,6
BA165_HD_BdTR2g	BdTR2g	402,6	473,2	1420,3	511,6
BA364_CD_BdTR2g	BdTR2g	1009,2	1135,3	2063,8	712,3
BA369_CD_BdTR2g	BdTR2g	806,3	537,8	578,2	230,2
BA073_HD_BdTR3c	BdTR3c	432,8	335,1	402,5	61,8
BA172_HD_BdTR3c	BdTR3c	676,9	752,3	1712,3	351,6
BA370_CD_BdTR3c	BdTR3c	542,5	585,9	354,6	53,6
BA424_CD_BdTR3c	BdTR3c	985,9	1131,8	1662	310,6
BA065_HD_BdTR5i	BdTR5i	358,7	296	254,4	57,5
BA082_HD_BdTR5i	BdTR5i	366,7	390,6	459,3	80,5
BA470_CD_BdTR5i	BdTR5i	367,3	369,8	607	94
BA473_CD_BdTR5i	BdTR5i	345,6	298,7	254,6	50,9
BA002_HD_BdTR9k	BdTR9k	377,8	193,6	54,1	21,5
BA033_HD_BdTR9k	BdTR9k	400,2	201,3	104,5	29,4
BA363_CD_BdTR9k	BdTR9k	514,8	390,2	585,7	209,9
BA410_CD_BdTR9k	BdTR9k	454,2	259,8	136,3	52,7
BA110_HD_Bis1	Bis1	606,4	739,8	1165,4	357,5
BA142_HD_Bis1	Bis1	544,7	393,2	352,6	133,2
BA373_CD_Bis1	Bis1	455,8	282,8	276,4	64,5

BA148_HW_BdTR11i	BdTR11i	230,3	39,3	4,2	1,4
BA377_CW_BdTR11i	BdTR11i	258,1	27,6	4	2,2
BA526_CW_BdTR11i	BdTR11i	443,8	72,9	8,3	5,2
BA022_HW_BdTR13a	BdTR13a	345,6	79,5	5,7	0
BA174_HW_BdTR13a	BdTR13a	324	45,8	2,6	2,6
BA371_CW_BdTR13a	BdTR13a	213,6	23,8	6,7	0
BA405_CW_BdTR13a	BdTR13a	310,6	30,7	14,6	3,8
BA070_HW_BdTR1i	BdTR1i	274,8	23,6	2,5	0
BA155_HW_BdTR1i	BdTR1i	375,1	48,1	5,9	3,8
BA469_CW_BdTR1i	BdTR1i	409,4	34,5	9	3,3
BA486_CW_BdTR1i	BdTR1i	348,4	78,2	5,4	2,6
BA115_HW_BdTR2b	BdTR2b	348,7	11,1	0	0
BA133_HW_BdTR2b	BdTR2b	270,5	58,2	6,4	3,9
BA378_CW_BdTR2b	BdTR2b	259,5	14,5	3,5	1,2
BA503_CW_BdTR2b	BdTR2b	377,6	73,2	10,9	2,1
BA107_HW_BdTR2g	BdTR2g	475,5	44,3	3,2	4,4
BA167_HW_BdTR2g	BdTR2g	401,5	36,3	7,7	3,3
BA445_CW_BdTR2g	BdTR2g	334,5	41,6	5,2	4,2
BA527_CW_BdTR2g	BdTR2g	594,9	48,9	7,4	2,5
BA071_HW_BdTR3c	BdTR3c	349,4	22	2,1	1,4
BA129_HW_BdTR3c	BdTR3c	227,6	38,8	2,6	2,6
BA362_CW_BdTR3c	BdTR3c	368,9	105,8	162,7	53,3
BA427_CW_BdTR3c	BdTR3c	234,2	75,7	8,1	3,9
BA160_HW_BdTR5i	BdTR5i	258,8	48,1	3,3	0,4
BA372_CW_BdTR5i	BdTR5i	119,9	23,4	9,3	1,5
BA464_CW_BdTR5i	BdTR5i	455,1	38,7	6,5	5,2
BA113_HW_BdTR9k	BdTR9k	270,8	55,8	0	0
BA125_HW_BdTR9k	BdTR9k	282,5	32,3	5,4	2,7
BA383_CW_BdTR9k	BdTR9k	309,8	41,3	2,6	0,4
BA515_CW_BdTR9k	BdTR9k	0	0	0	0
BA118_HW_Bis1	Bis1	252,7	32,8	2,3	0
BA156_HW_Bis1	Bis1	577,5	32,3	10,6	1,1
BA385_CW_Bis1	Bis1	237,8	29,9	3,4	1,7
BA390_CW_Bis1	Bis1	276,9	51,3	4,2	0,6
BA147_HW_Kah1	Kah1	167,3	41,4	3,1	0
BA169_HW_Kah1	Kah1	218	68,9	8,6	1,5
BA356_CW_Kah1	Kah1	135	47,9	3	0,8

BA519_CD_Bis1	Bis1	288,5	152,2	181,6	43,3
BA046_HD_Kah1	Kah1	251,7	91,2	14,7	1,5
BA168_HD_Kah1	Kah1	184,9	185,4	223,9	41
BA382_CD_Kah1	Kah1	552,3	517,7	470,1	81,4
BA495_CD_Kah1	Kah1	552,7	775	912,6	139,7
BA048_HD_Kah5	Kah5	401,4	219,2	144,2	44,3
BA095_HD_Kah5	Kah5	386,8	648,6	480,8	58,5
BA401_CD_Kah5	Kah5	444,7	708	526,8	88,6
BA489_CD_Kah5	Kah5	498,2	867	776	154,1
BA127_HD_Koz1	Koz1	337,2	319,5	341,6	41,9
BA132_HD_Koz1	Koz1	377,6	429,1	638,9	75,8
BA395_CD_Koz1	Koz1	458,4	415,4	127,4	27
BA507_CD_Koz1	Koz1	505,3	585,8	388	84,8
BA081_HD_Koz3	Koz3	523,6	592,8	892,7	120
BA388_CD_Koz3	Koz3	1094,8	1066,9	5661,6	1244,7
BA467_CD_Koz3	Koz3	1201,8	1447,1	4545,9	1203
BA089_HD_Koz-3	Koz3	773,9	1189,3	2664,1	681,1
BA035_HD_Ron2	Ron2	1429,9	1947,5	1398,8	364,2
BA379_CD_Ron	Ron2	264	166	74,8	15,9
BA432_CD_Ron2	Ron2	274,6	254,9	202,5	41,2
BA420_CW_Kah1	Kah1	254,1	46,2	11,5	3,2
BA047_HW_Kah5	Kah5	280,1	28,2	5,6	0,9
BA158_HW_Kah5	Kah5	268,7	128,5	16	3,1
BA399_CW_Kah5	Kah5	351,8	34	19,5	7,3
BA482_CW_Kah5	Kah5	315,1	30,4	5,6	2
BA141_HW_Koz1	Koz1	323,6	22,5	1	1
BA157_HW_Koz1	Koz1	353	68,7	9,5	0,6
BA501_CW_Koz1	Koz1	415,6	43,4	4,3	2,9
BA511_CW_Koz1	Koz1	397,2	79,4	31,8	10,6
BA057_HW_Koz3	Koz3	325,3	18,2	1,3	0
BA074_HW_Koz3	Koz3	435,3	20,5	2	1,1
BA411_CW_Koz3	Koz3	283,6	28,8	15,3	3,7
BA484_CW_Koz3	Koz3	332,2	47,8	9,3	2,3
BA026_HW_Ron2	Ron2	218	122,8	15,3	1,7
BA151_HW_Ron2	Ron2	350,5	38,4	7	1,5
BA409_CW_Ron2	Ron2	296,4	36,6	73,2	32,9
BA431_CW_Ron2	Ron2	259,2	15,4	6,5	4

Supplementary Table S8. Summary statistics of dehydrin *Bdhn*1a, *Bdhn*2, *Bdhn*3 and *Bdhn*7 gene expressions under dry (D) vs watered (W) conditions and comparative differential expression (DE) tests in *B. distachyon* ecotypes. **(a)** Kruskal-Wallis rank tests (D vs W) for each *Bdhn* gene. **(b)** Wilcoxon pairwise tests of normalized TPM values across ecotypes, p-values were adjusted with the Benjamini–Hochberg (BH) procedure, controlling the false discovery rate, to correct for multiple comparisons; n. s., non significant, p≤ 0.05* significant values are highlighted in bold.

(a)

Var	***Bdhn*1a**	***Bdhn*2**	***Bdhn*3**	***Bdhn*7**
H test	123.74	179.98	178.8	175.02
p-value	7.69E-06	3.38E-13	5.10E-15	1.75E-12

(b)

	***Bdhn*1a**			***Bdhn*2**			***Bdhn*3**			***Bdhn*7**		
**Ecotype**	**D**	**W**	**W-test**	**D**	**W**	**W-test**	**D**	**W**	**W-test**	**D**	**W**	**W-test**
**ABR2**	214.175	159.875	n.s	**167.1**	**46.05**	*	**172.425**	**18.65**	*	**19.4**	**2.3**	*
**ABR3**	263.775	215.833	n.s	169.05	48.467	n.s	219.875	15.167	n.s	29.525	2.3	n.s
**ABR4**	247.15	269.375	n.s	108.425	48.4	n.s	**62.175**	**5.025**	*	15.1	2.4	n.s
**ABR5**	252.633	272.233	n.s	311.233	37.6	n.s	294.767	6.967	n.s	51.6	2.967	n.s
**ABR6**	290.175	302.233	n.s	**204.525**	**23.433**	*	850.55	3.8	n.s	41.55	3	n.s
**ABR8**	530.05	255.333	n.s	359.425	31.767	n.s	231.05	4.367	n.s	127.775	1.333	n.s
**Adi10**	659.4	532.767	n.s	935.575	40.133	n.s	1216.925	8.4	n.s	332.9	4.167	n.s
**Adi12**	488.65	418.675	n.s	**591.725**	**68.75**	*	**498.525**	**11.75**	*	**139.825**	**4.45**	*
**Adi2**	461.1	430	n.s	511	56.733	n.s	437.6	3.367	n.s	63.567	3	n.s
**Bd1_1**	414.3	355.45	n.s	152.35	32.25	n.s	45.95	11.25	n.s	14.95	2.75	n.s
**Bd18-1**	736.95	259.3	n.s	548.1	19	n.s	410.6	3.9	n.s	172.9	0.45	n.s
**Bd21-3**	632.767	290.767	n.s	890.1	25.8	n.s	839.567	14.3	n.s	360.667	1.5	n.s
**Bd21ctrl**	326.85	300.1	n.s	113.725	44.7	n.s	30.575	16.375	n.s	24.325	3.35	n.s
**Bd2-3**	502.175	368.35	n.s	202.05	55.675	n.s	94.825	3.925	n.s	29.275	2.5	n.s
**Bd30-1**	488.675	365.05	n.s	**761.7**	**33.575**	*	**906.55**	**5.725**	*	**222.7**	**2.3**	*
**Bd3-1**	395.167	361.967	n.s	465.367	41.367	n.s	1711.533	9.367	n.s	329.433	1.233	n.s
**BdTR10c**	410.375	200.2	n.s	327.65	43	n.s	924.275	4.2	n.s	194.1	1.75	n.s
**BdTR11g**	497.15	388.925	n.s	**454.7**	**59.35**	*	**643.075**	**17.6**	*	**95.65**	**7.2**	*
**BdTR11i**	531.875	325.433	n.s	553.2	43.533	n.s	918.9	5.033	n.s	124.325	3.167	n.s

**BdTR13a**	697.567	298.45	n.s	434.5	44.95	n.s	313.733	7.4	n.s	**114.633**	**1.6**	*
**BdTr1i**	582.925	351.925	n.s	**640.25**	**46.1**	*	**727.325**	**5.7**	*	**251.15**	**2.425**	*
**BdTR2b**	538.175	318.55	n.s	405.55	43.85	n.s	436.05	7.2	n.s	136.4	1.65	n.s
**BdTR2g**	750.55	451.6	n.s	**816.325**	**42.775**	*	**1517.95**	**5.875**	*	**517.675**	**3.6**	*
**BdTR3c**	659.525	317.5	n.s	701.275	67.833	n.s	1032.85	57.633	n.s	194.4	19.533	n.s
**BdTR5i**	364.233	277.933	n.s	352.133	36.733	n.s	440.233	6.367	n.s	77.333	2.367	n.s
**BdTR9k**	436.75	197.433	n.s	261.225	24.533	n.s	220.15	2.667	n.s	78.375	1.033	n.s
**Bis1**	450.233	364.067	n.s	391.6	37.833	n.s	541.133	6.067	n.s	155.1	1.133	n.s
**Kah1**	385.4	202.367	n.s	392.325	54.333	n.s	405.325	7.7	n.s	65.9	1.833	n.s
**Kah5**	**432.775**	**303.925**	*	**610.7**	**55.275**	*	**481.95**	**11.675**	*	**86.375**	**3.325**	*
**Koz1**	481.85	388.6	n.s	500.6	63.833	n.s	257.7	15.2	n.s	55.9	4.7	n.s
**Koz3**	**898.525**	**344.1**	*	**1074.025**	**28.825**	*	**3441.075**	**6.975**	*	**812.2**	**1.775**	*
**RON2**	656.167	288.3	n.s	789.467	65.933	n.s	558.7	31.833	n.s	140.433	12.033	n.s

Supplementary Table S9. Linear model (lm) regression analysis for comparative differential gene expressions of dehydrin *Bdhn* genes in the studied *B. distachyon* ecotypes. W (watered) and D (dry) conditions. Significant p-values (p≤0.001***).

W+D	***Bdhn*1a ~ *Bdhn*2**	***Bdhn*1a ~ *Bdhn*3**	***Bdhn*1a ~ *Bdhn*7**	***Bdhn*2 ~ *Bdhn*3**	***Bdhn*2 ~ *Bdhn*7**	***Bdhn*3 ~ *Bdhn*7**
Median	-11.12	-14.77	-14.05	-68.64	-72.05	-37.35
Residual standard error:	134.6	169.5	156.3	220.8	210.7	237
F-statistic:	368	148.3	214.6	344.9	401	1840
p-value:	< 2.20E-16***	< 2.20E-16***	< 2.20E-16***	< 2.20E-16***	< 2.20E-16***	< 2.20E-16***

Supplementary Table S10. Comparative analysis of dehydrin genes showing upregulated expression under drought compared to watered conditions in *Brachypodium distachyon* and *Triticum aestivum*. Orthology between the differentially expressed genes in the two species was retrieved through Ensembl Plants using BioMart and Blast searches using orthologies previously established in Galvez et al. (2019) (§).

Brachypodium distachyon			***Triticum aestivum***			
**Gene name**	**RefSeq.v3.0 Name**	**Ref.Seqv3.1 Name**	**Original gene name**	**Gene name in Supplementary Table 1**	**RefSeq v2.1 Name**	**Differentially expressed genes under drought conditions (Galvez et al. 2019)**
*Bdhn*1	BRADI_5g10860v3	Bradi5g10860	DHN11-A1	DHNTaes 1	TraesCS6A02G253300	Mild stress
*Bdhn*2	BRADI_3g51200v3	Bradi3g51200	DHN11-B1	DHNTaes 3	TraesCS6B02G273400	
			DHN11-D1	DHNTaes 2	TraesCS6D02G234700	
*Bdhn*3	BRADI_1g37410v3	Bradi1g37410	DHN4-B1	DHNTaes 9	TraesCS6B02G383500	Severe Stress
			DHN4-D1	DHNTaes 4	TraesCS6D02G332900	Severe Stress
			DHN3-A1	DHNTaes 12	TraesCS6A02G350600	Mild stress
			DHN3-A5	DHNTaes 13	TraesCS6A02G350800	
			DHN3-A6	DHNTaes 8	TraesCS6A02G350700	Mild stress
			DHN3-B6	DHNTaes 10	TraesCS6B02G383600	Mild stress
			DHN3-D6	DHNTaes 5	TraesCS6D02G333200	Severe Stress
			DHN3-D4	DHNTaes 11	TraesCS6D02G333100	Severe Stress
			DHN3-D8	DHNTaes 6	TraesCS6D02G333300	
			DHN3-D9	DHNTaes 7	TraesCS6D02G333600	Severe Stress
*Bdhn*7	BRADI_3g43870v3	Bradi3g43870	DHN38-A1	DHNTaes 14	TraesCS5A02G424700	
			DHN38-B1	DHNTaes 16	TraesCS5B02G426700	Severe Stress
			DHN38-D1§	DHNTaes 18§	TraesCS5D01G433200	
			DHN38-A2	DHNTaes 15	TraesCS5A02G424800	
			DHN38-B2§	DHNTaes 17§	TraesCS5B01G426800	Severe Stress
			DHN38-D2	DHNTaes 19	TraesCS5D02G433300	

Supplementary Table S11. Summary statistics of 12 drought-response phenotypic traits [leaf_rwc (relative water content in leaf); leaf_wc (water content in leaf); lma (leaf mass per área); pro (leaf proline content); abvrgd (above ground biomass); blwgrd (below ground biomass); ttlmass (total mass); rmr (root mass ratio); delta13c (carbon isotope, a proxy for lifetime integrated WUE); leafc (leaf carbon content); leafn (leaf nitrogen content); cn (leaf carbon/nitrogen ratio)] in dry (D) vs watered (W) *Brachypodium distachyon* plants. **(a)** Kruskall-Wallis rank tests (W vs D) for each phenotypic trait. **(b)** comparative pairwise Wilcoxon tests in the studied *B. distachyon* ecotypes; p-values were adjusted with the Benjamini–Hochberg (BH) procedure, controlling the false discovery rate, to correct for multiple comparisons; n, number of replicates. n. s., non significant, *p≤ 0.05*; significant values are highlighted in bold.

(a)

**Var**	**Leaf_rwc**	**Leaf_wc**	**Lma**	**Pro**	**abvgrd**	**blwgrd**	**ttlmas**	**rmr**	**WUE**	**leafC**	**LeafN**	**C:N**
**t-test**	197.24	172.97	166.68	189.78	190.61	161.24	181.31	192.97	192.67	169.61	188.34	188.8
**df**	63	63	63	63	63	63	63	63	63	63	63	63
**p-value**	9.46E-16***	3.42E-12***	2.62E-11***	1.24E-14***	9.32E-15***	1.47E-10***	2.17E-13***	4.15E-15***	4.60E-15***	1.02E-11***	2.02E-14***	7.32E-15***

(b)

		**leaf_rwc**			**leafwc**			**lma**			**pro**			**abvgrd**			**blwgrd**		
**Ecotype**	**n**	**D**	**W**	**W-test**	**D**	**W**	**W-test**	**D**	**W**	**W-test**	**D**	**W**	**W-test**	**D**	**W**	**W-test**	**D**	**W**	**W-test**
ABR2	4	**95.92**	**99.09**	*	**275.41**	**327.41**	*	**31.09**	**27.07**	*	**39.54**	**17.51**	*	82.39	82.83	n.s	54.70	39.94	n.s
ABR3	4	**95.91**	**99.23**	*	325.50	341.29	n.s	24.83	23.35	n.s	**18.48**	**9.26**	*	94.28	113.30	n.s	57.44	52.51	n.s
ABR4	4	**96.62**	**99.44**	*	**312.84**	**357.86**	*	28.94	27.19	n.s	**15.44**	**8.76**	*	**53.93**	**63.83**	*	**32.19**	**29.34**	*
ABR5	3	95.28	100.14	n.s	309.74	355.00	n.s	27.51	23.67	n.s	12.79	7.68	n.s	83.10	84.11	n.s	55.12	33.31	n.s
ABR6	4	**95.07**	**99.73**	*	**300.31**	**334.41**	*	**27.93**	**25.84**	*	**24.37**	**10.16**	*	72.96	80.03	n.s	**40.48**	**30.95**	*
ABR8	4	**94.01**	**99.09**	*	327.95	359.65	n.s	**30.93**	**27.92**	*	**25.45**	**8.00**	*	**112.43**	**172.49**	*	**33.23**	**40.93**	*
Adi10	4	**92.50**	**99.49**	*	**333.21**	**372.90**	*	**28.86**	**24.35**	*	**54.79**	**10.94**	*	**133.87**	**225.64**	*	67.65	80.73	n.s
Adi12	4	**92.10**	**99.41**	*	**291.01**	**322.88**	*	**29.34**	**26.69**	*	**30.06**	**10.47**	*	**141.38**	**168.85**	*	**70.54**	**57.79**	*
Adi2	3	94.99	97.91	n.s	282.54	307.59	n.s	28.99	25.70	n.s	40.57	8.61	n.s	102.75	161.41	n.s	55.39	49.99	n.s
Bd1-1	2	96.65	99.49	n.s	318.06	340.96	n.s	27.89	26.05	n.s	22.48	8.68	n.s	69.95	70.85	n.s	32.68	28.03	n.s
Bd18-1	2	92.46	98.01	n.s	305.47	330.20	n.s	29.18	26.35	n.s	26.56	9.00	n.s	127.49	166.53	n.s	60.50	52.55	n.s
Bd21ctrl	4	**94.72**	**98.53**	*	348.66	370.79	n.s	29.95	28.19	n.s	**16.88**	**7.80**	*	104.63	104.94	n.s	**48.95**	**34.90**	*
Bd21-3	3	88.24	99.96	n.s	300.78	333.15	n.s	34.40	29.66	n.s	46.00	7.99	n.s	132.92	166.58	n.s	76.00	57.59	n.s
Bd2-3	4	**94.68**	**99.54**	*	301.70	323.22	n.s	**29.30**	**27.49**	*	**24.56**	**7.18**	*	**109.68**	**142.81**	*	52.55	48.69	n.s
Bd30-1	4	**91.94**	**98.74**	*	**290.94**	**360.88**	*	**29.98**	**24.18**	*	**39.52**	**8.27**	*	**108.47**	**150.05**	*	**55.83**	**70.51**	*
Bd3-1	3	**87.95**	**98.93**	*	**314.60**	**346.29**	*	**30.34**	**26.74**	*	**39.47**	**11.38**	*	**147.08**	**202.11**	*	64.45	66.88	n.s
BdTR10c	4	92.18	99.85	n.s	292.36	346.45	n.s	28.54	24.40	n.s	62.13	9.26	n.s	122.84	179.83	n.s	54.84	78.85	n.s
BdTR11g	4	**92.67**	**99.17**	*	**303.40**	**348.85**	*	30.29	28.51	n.s	**26.36**	**6.46**	*	**118.32**	**162.40**	*	58.96	59.50	n.s
BdTR11i	4	**92.16**	**98.87**	*	**301.41**	**356.48**	*	**31.49**	**26.81**	*	**44.23**	**7.77**	*	**123.81**	**151.69**	*	54.83	57.03	n.s

BdTR13a	3	**94.01**	**99.13**	*	**298.81**	**335.52**	*	**30.04**	**28.16**	*	**20.39**	**7.56**	*	**101.63**	**130.20**	*	50.92	49.18	n.s
BdTR1i	4	**91.62**	**98.50**	*	**291.95**	**310.40**	*	**27.84**	**25.64**	*	**42.46**	**11.32**	*	**132.79**	**172.05**	*	67.58	65.85	n.s
BdTR2b	4	90.58	99.16	n.s	286.28	334.79	n.s	30.04	25.59	n.s	32.55	9.08	n.s	135.43	184.43	n.s	69.31	74.33	n.s
BdTR2g	4	**88.23**	**99.03**	*	**275.58**	**321.83**	*	**30.19**	**25.78**	*	**54.82**	**10.80**	*	141.09	152.06	n.s	78.53	55.90	n.s
BdTR3c	4	**91.01**	**99.87**	*	**287.62**	**335.43**	*	**29.31**	**24.88**	*	**29.50**	**8.46**	*	106.26	174.36	n.s	58.46	55.66	n.s
BdTR5i	3	92.84	98.68	n.s	292.29	324.81	n.s	31.54	26.77	n.s	42.44	9.39	n.s	97.23	141.26	n.s	62.53	64.18	n.s
BdTR9k	4	**95.21**	**98.97**	*	303.20	315.82	n.s	27.63	28.14	n.s	**19.61**	**14.46**	*	133.27	151.78	n.s	58.40	51.30	n.s
Bis1	3	93.77	98.41	n.s	300.90	329.00	n.s	29.34	25.63	n.s	21.24	7.71	n.s	97.16	113.62	n.s	45.96	41.93	n.s
Kah1	4	**94.02**	**99.23**	*	315.47	334.60	n.s	29.18	28.18	n.s	**31.07**	**9.33**	*	**109.04**	**136.56**	*	**55.51**	**48.92**	*
Kah5	4	**93.11**	**98.86**	*	311.66	343.46	n.s	27.96	26.56	n.s	**33.53**	**7.36**	*	**122.20**	**161.41**	*	48.54	50.30	n.s
Koz1	2	95.95	99.58	n.s	332.48	335.81	n.s	26.64	26.38	n.s	16.15	7.35	n.s	82.20	135.72	n.s	56.78	46.36	n.s
Koz3	4	**85.40**	**99.62**	*	**310.63**	**366.61**	*	**30.12**	**23.45**	*	**44.27**	**5.49**	*	122.40	167.18	n.s	62.03	56.69	n.s
RON2	3	94.61	99.50	n.s	335.71	366.35	n.s	27.23	24.52	n.s	30.60	7.88	n.s	93.14	133.44	n.s	63.23	62.31	n.s

		ttlmass			rmr			WUE			leafc			leafn			cn		
**Ecotype**	**n**	**D**	**W**	**W-test**	**D**	**W**	**W-test**	**D**	**W**	**W-test**	**D**	**W**	**W-test**	**D**	**W**	**W-test**	**D**	**W**	**W-test**
ABR2	4	137.09	122.76	n.s	**38.89**	**33.48**	*	**11.32**	**10.27**	*	-31.00	-31.29	n.s	**397.18**	**394.22**	*	**35.17**	**39.21**	*
ABR3	4	151.72	165.80	n.s	**37.51**	**31.32**	*	12.01	9.45	n.s	**-30.66**	**-31.50**	*	**401.27**	**389.46**	*	**33.47**	**41.64**	*
ABR4	4	86.11	93.11	n.s	**37.90**	**31.43**	*	**11.37**	**9.41**	*	-30.98	-31.69	n.s	**402.26**	**397.76**	*	**35.51**	**42.42**	*
ABR5	3	138.14	117.42	n.s	39.48	27.78	n.s	10.80	9.06	n.s	-31.21	-32.05	n.s	397.90	389.95	n.s	37.03	43.31	n.s
ABR6	4	113.44	110.98	n.s	**35.52**	**27.32**	*	**11.77**	**9.26**	*	**-31.30**	**-31.93**	*	**404.47**	**389.55**	*	**34.51**	**42.18**	*
ABR8	4	**145.65**	**213.41**	*	**23.44**	**19.36**	*	12.49	11.39	n.s	**-31.33**	**-31.58**	*	393.03	383.02	n.s	31.77	34.08	n.s
Adi10	4	**201.51**	**305.91**	*	**33.44**	**26.40**	*	**12.53**	**12.19**	*	**-30.84**	**-31.58**	*	397.39	388.03	n.s	31.74	32.45	n.s
Adi12	4	211.91	226.64	n.s	**33.45**	**24.94**	*	**12.47**	**10.13**	*	**-30.34**	**-31.28**	*	**400.60**	**390.93**	*	**32.22**	**38.80**	*
Adi2	3	158.14	211.40	n.s	34.89	23.65	n.s	12.80	11.38	n.s	-30.63	-31.27	n.s	396.23	389.55	n.s	31.17	34.69	n.s
Bd1-1	2	102.62	98.88	n.s	32.62	28.27	n.s	12.27	8.92	n.s	-30.71	-31.69	n.s	396.41	386.51	n.s	32.63	43.38	n.s
Bd18-1	2	188.09	219.08	n.s	31.97	23.64	n.s	12.44	11.16	n.s	-30.57	-31.84	n.s	407.79	397.53	n.s	32.83	36.54	n.s
Bd21ctrl	4	**153.58**	**139.84**	*	**31.91**	**25.87**	*	**12.58**	**9.82**	*	**-30.22**	**-31.43**	*	**403.73**	**388.69**	*	**32.31**	**39.63**	*
Bd21-3	3	208.92	224.17	n.s	35.09	25.19	n.s	11.57	11.10	n.s	-31.03	-31.93	n.s	396.29	389.81	n.s	34.28	35.17	n.s
Bd2-3	4	**162.12**	**191.49**	*	**32.24**	**25.42**	*	12.63	10.64	n.s	**-30.73**	**-31.27**	*	**394.31**	**381.89**	*	**31.43**	**36.48**	*
Bd30-1	4	**164.29**	**220.57**	*	34.51	32.28	n.s	**11.14**	**10.05**	*	**-31.16**	**-32.11**	*	**386.45**	**376.32**	*	**34.98**	**37.58**	*
Bd3-1	3	**211.52**	**268.99**	*	**31.01**	**24.91**	*	**13.24**	**10.75**	*	**-30.66**	**-32.08**	*	**394.78**	**387.37**	*	**29.94**	**36.25**	*
BdTR10c	4	177.68	258.67	n.s	31.05	30.12	n.s	13.38	11.49	n.s	-30.71	-31.65	n.s	402.76	390.60	n.s	30.43	34.09	n.s
BdTR11g	4	**177.28**	**221.90**	*	**33.12**	**26.75**	*	**12.31**	**11.47**	*	**-31.34**	**-32.07**	*	401.69	391.18	n.s	32.85	34.58	n.s
BdTR11i	4	**178.64**	**208.72**	*	**31.09**	**27.21**	*	**12.52**	**10.94**	*	**-31.11**	**-31.91**	*	399.66	384.59	n.s	32.06	36.11	n.s
BdTR13a	3	**152.54**	**179.38**	*	**33.35**	**27.44**	*	**11.89**	**10.37**	*	-31.26	-32.08	n.s	**399.54**	**393.72**	*	**33.74**	**38.07**	*

BdTR1i	4	200.37	237.90	n.s	**33.23**	**26.66**	*	**12.99**	**11.00**	*	**-30.33**	**-31.11**	*	**401.43**	**389.86**	*	**31.01**	**35.67**	*
BdTR2b	4	204.81	258.75	n.s	33.70	28.43	n.s	12.36	9.81	n.s	-30.46	-31.97	n.s	402.35	396.22	n.s	32.69	40.70	n.s
BdTR2g	4	219.62	207.97	n.s	**35.40**	**27.05**	*	**12.52**	**12.08**	*	-30.43	-31.05	n.s	397.63	392.77	n.s	31.94	33.34	n.s
BdTR3c	4	**164.73**	**230.02**	*	**35.42**	**24.14**	*	**13.88**	**11.71**	*	**-30.84**	**-31.73**	*	**404.11**	**392.73**	*	**29.21**	**33.63**	*
BdTR5i	3	159.77	205.44	n.s	38.12	30.36	n.s	12.04	10.29	n.s	-31.08	-31.94	n.s	400.25	389.96	n.s	33.50	38.15	n.s
BdTR9k	4	191.67	203.08	n.s	**31.27**	**25.03**	*	**12.35**	**10.09**	*	**-30.75**	**-31.74**	*	**405.45**	**385.74**	*	**33.06**	**38.39**	*
Bis1	3	143.08	155.54	n.s	33.24	27.64	n.s	12.22	10.28	n.s	-31.34	-32.29	n.s	400.67	391.98	n.s	32.82	38.27	n.s
Kah1	4	164.55	185.48	n.s	**33.74**	**26.03**	*	**12.89**	**9.85**	*	**-30.89**	**-31.79**	*	**402.98**	**390.96**	*	**31.44**	**39.79**	*
Kah5	4	**170.74**	**211.70**	*	**28.57**	**23.54**	*	**12.54**	**10.60**	*	**-30.50**	**-31.22**	*	**403.40**	**393.09**	*	**32.24**	**37.22**	*
Koz1	2	138.98	182.08	n.s	40.08	25.48	n.s	12.58	10.89	n.s	-31.28	-31.70	n.s	404.89	397.19	n.s	32.45	36.64	n.s
Koz3	4	184.43	223.87	n.s	**33.05**	**25.26**	*	**13.20**	**11.25**	*	**-30.74**	**-31.80**	*	**405.07**	**393.25**	*	**30.73**	**35.26**	*
RON2	3	157.49	195.76	n.s	38.91	31.37	n.s	11.70	10.03	n.s	-30.61	-31.32	n.s	404.26	396.07	n.s	35.48	39.74	n.s

Supplementary Table S12. Linear model (lm) regression analysis for comparative *Brachypodium distachyon Bdhn* gene expressions and drought-induced phenotypic trait changes under total watered (W) and dry (D) conditions. Significant p-values (p≤ 0.05*; 0.01**; 0.001***).

	*Bdhn*1a				*Bdhn*2				*Bdhn*3				*Bdhn*7			
traits	median	Std error	F-statistic	p-value	median	Std error	F-statistic	p-value	median	Std error	F-statistic	p-value	median	Std error	F-statistic	p-value
leaf_rwc	-34.83	196.9	51.03	1.227E-11 ***	-41.95	265.3	169.3	<2E-16***	-8.7	564.8	136.8	<2E-16***	-4.23	145.1	129.7	<2E-16***
leaf_wc	-49.32	213.2	10.19	0.001609**	-70.69	321.3	43.09	3.51E-10***	-166.9	682.3	22.33	4.02E-06***	-43.52	174.8	16.68	2.32E-05***
lma	-54.08	213.8	9.029	0.0025955 **	-96.58	321.2	43.25	3.28E-10***	-188	689.2	17.37	4.38E05***	-46.48	175.3	17.44	4.22E-05***
pro	-46.12	210.7	15.82	9.36E-05***	-93.89	308.5	66.03	2.85E-14***	-127.6	649.7	47.99	4.37E-11***	-30.73	166.5	43.91	2.47E-10***
abvgrd	-53.51	217.9	0.1635	0.6864	-136.1	346.7	4.911	0.0277*	-270.9	713.4	1.055	0.30554	-71.82	181.8	0.08986	0.765
blwgrd	-40.97	208.7	20.63	9.07E-06***	-72.33	333.1	24.22	1.65E-06***	-165.2	687	18.89	2.09E-05***	-38.5	173	23.87	1.95E-06***
ttlmass	-52.88	216.7	2.607	0.1078	-175	350.4	0.1187	0.73078	-293.4	714.8	0.1826	0.6695	-58.21	181.3	1.305	0.255
rmr	-35.02	213.1	10.56	0.001331**	-77.24	313.3	56.99	1.06E-12***	-152.7	674.7	27.97	2.90E-07***	-41.8	174	20.85	8.16E-06***
WUE	-129.55	206.2	26.68	5.26E-07***	-52.93	308	66.88	2.04E-14***	-122.5	664.9	35.53	9.50E-09***	-31.64	167.8	39.49	1.67E-09***
leafC	-39.98	210.7	15.95	8.80E-05***	-90.2	323.7	39.07	2E-09***	-183.4	682.8	21.94	4.85E-06***	-50.17	175.8	15.87	9.14E-05***
leafN	-42.83	199.8	43.07	3.54E-10***	-62.2	311.9	59.54	3.77E-13***	-160.7	658.5	40.66	1.E-09***	-44.7	169	36.01	7.69E-09***
CN	-40.24	198.4	46.9	6.93E-11***	-58.73	307.7	67.44	1.64E-14***	-131.6	652.1	45.95	1.04E-10***	-33.83	168	39.09	1.98E-09***

Supplementary Table S13. Phylogenetic signal of dehydrin gene expressions under watered (W) and dry (D) conditions, drought-induced phenotypic traits changes and climate niche variation assessed in **(a)** the *B. distachyon* nuclear-SNP tree and **(b)** the *B. distachyon Bdhn* tree using the *phylosig* option of the *phytools* R package. Blomberg's K and Pagel's lambda values close to one indicate phylogenetic signal and values close to zero phylogenetic independence. K, p-values based on 1000 randomizations; lambda, p-values based on the Likelihood Ratio test. Significant and marginal significant values are highlighted in bold.

(a)

***Bdhn* gene**	**Treatment**	**K**	**P-value**	**lambda(ʎ)**	**logL(ʎ)**	**LR(ʎ=0)**	**P-value**
*Bdhn*1aW	W	0.00207548	0.579	6.6113E-05	-170094	-9.01E-04	1
*Bdhn*2W		0.00467069	0.235	6.6113E-05	-111372	-9.77E-04	1
*Bdhn*3W		0.00236035	0.553	6.6113E-05	-90.2705	-1.13E-03	1
*Bdhn*7W		0.00162038	0.695	6.6113E-05	-59.9152	-1.39E-03	1
*Bdhn*1aD	D	0.0038235	0.317	6.6113E-05	-194.936	-1.03E-03	1
*Bdhn*2D		0.00311909	0.364	6.6113E-05	-209.636	-1.03E-03	1
*Bdhn*3D		0.00257041	0.71	6.6113E-05	-237.428	-1.06E-03	1
*Bdhn*7D		0.00137242	0.753	6.6113E-05	-196.53	-1.04E-03	1

	Drought						Watered					
**Phenotypic trait**	**K**	**P-value (based on 1000 randomizations)**	**lambda(λ)**	**logL(λ)**	**LR(λ=0)**	**P-value**	**K**	**P-value**	**lambda(λ)**	**logL(λ)**	**LR(λ=0)**	**P-value**
leaf_rwc	0.00595303	0.202	0.8240433	-70.83287	-71.94764	0.1353942	0.00633355	0.137	6.6113E-05	-21.1366	-21.1361	1
leaf_wc	0.0191719	0.029	0.5318181	-129.0792	-129.2025	0.6194317	0.00601205	0.142	0.712778	-124.3118	-124.641	0.4171331
lma	0.00655413	0.198	0.4014225	-60.3207	-60.04825	1	**0.0127346**	**0.067**	0.5672695	-53.1973	-54.02652	0.1978138
pro	0.00178069	0.645	6.61128E-05	-118.2053	-118.2048	1	0.00751385	0.279	6.6113E-05	-65.7397	-65.73915	1
**abvrgd**	**0.0548587**	**0.005**	**0.9862024**	**-132.3802**	**-137.5324**	**0.00132712**	0.00982265	0.118	**0.9056281**	**-143.4905**	**-147.2048**	**0.00641901**
blwgrd	0.00799846	0.135	0.6778591	-112.6454	-113.232	0.2787568	0.00503958	0.192	0.372553	-117.8161	-117.602	1
**ttlmass**	**0.0254584**	**0.02**	**0.9586421**	**-144.0437**	**-147.1176**	**0.01315614**	0.00760606	0.15	**0.8313881**	**-153.3088**	**-155.4348**	**0.0392068**
rmr	0.0117638	0.1	**0.8587116**	**-71.60054**	**-74.00027**	**0.02846858**	**0.0198041**	**0.031**	**0.8500612**	**-65.57032**	**-69.23347**	**0.00679529**
**delta13c**	**0.0206935**	**0.023**	0.5455282	-9.438273	-9.102205	1	0.00079528	0.921	6.6113E-05	-9.565582	-9.565045	1
leafc	0.00564166	0.206	6.61128E-05	-86.81046	-86.80988	1	0.00344133	0.413	6.6113E-05	-84.79111	-84.79067	1
**leafn**	**0.0127895**	**0.066**	**0.8379002**	**-52.22653**	**-57.04403**	**0.00190906**	0.00111356	0.809	0.6412875	-71.1111	-72.40537	0.1076397
**cn**	**0.015233**	**0.051**	**0.8350859**	**-24.49639**	**-28.06983**	**0.00750945**	0.00101427	0.842	0.6145648	-35.11085	-36.01121	0.1796235

**Climate trait**	**K**	**p-value**	**lambda(λ)**	**logL(λ)**	**LR(λ=0)**	**p-value**
**PCA1**	**0.0201727**	**0.024**	**0.905784**	**-70.641**	**12.567**	**0.000392627**

(b)

Bdhn gene	Treatment	K	p-value	lambda(λ)	logL(λ)	LR(λ=0)	p-value
*Bdhn*1aW	W	0.231879	0.142	0.782475	-175.48	1.121	0.289703
***Bdhn*2W**		**0.34558**	**0.017**	**0.759422**	**-112.036**	**6.78197**	**0.00920834**
*Bdhn*3W		0.10957	0.603	6.64306E-05	-93.2539	0.00063134	1
***Bdhn*7W**		**0.433394**	**0.031**	**0.97584**	**-60.2457**	**3.42969**	**0.0640339**
*Bdhn*1aD	D	0.308794	0.051	0.502054	-194.065	0.710376	0.399319
*Bdhn*2D		0.211919	0.176	6.64306E-05	-208.371	0.00063094	1
***Bdhn*3D**		**0.433425**	**0.064**	**0.951732**	**-234.916**	**4.48918**	**0.0341101**
*Bdhn*7D		0.379302	0.083	0.917646	-195.278	1.74672	0.18629

Phenotypic trait	K	p-value	lambda(λ)	logL(λ)	LR(λ=0)	p-value
leaf_rwcW	0.00633355	0.137	6.61E-05	-21.1366	-21.1361	1
leaf_wcW	0.00601205	0.142	0.712778	-124.3118	-124.641	0.4171331
lmaW	0.0127346	0.067	0.5672695	-53.1973	-54.02652	0.1978138
proW	0.00751385	0.279	6.61E-05	-65.7397	-65.73915	1
**abvrgdW**	**0.00982265**	**0.118**	**0.9056281**	**-143.4905**	**-147.2048**	**0.006419007**
blwgrdW	0.00503958	0.192	0.372553	-117.8161	-117.602	1
ttlmassW	0.00760606	0.15	0.8313881	-153.3088	-155.4348	0.0392068
**rmrW**	**0.0198041**	**0.031**	**0.8500612**	**-65.57032**	**-69.23347**	**0.006795293**
WUE_W	0.00079528	0.921	6.61E-05	-9.565582	-9.565045	1
leafcW	0.00344133	0.413	6.61E-05	-84.79111	-84.79067	1
**leafnW**	**0.00111356**	**0.809**	**0.6412875**	**-71.1111**	**-72.40537**	**0.1076397**
**cnW**	**0.00101427**	**0.842**	**6.15E-01**	**-35.11085**	**-36.01121**	**0.1796235**
**leaf_rwcD**	**0.00595303**	**0.202**	**0.8240433**	**-70.83287**	**-71.94764**	**0.1353942**
leaf_wcD	0.0191719	0.029	0.5318181	-129.0792	-129.2025	0.6194317
lmaD	0.00655413	0.198	0.4014225	-60.3207	-60.04825	1
proD	0.00178069	0.645	6.61E-05	-118.2053	-118.2048	1
**abvrgdD**	**0.0548587**	**0.005**	**0.9862024**	**-132.3802**	**-137.5324**	**0.001327115**
blwgrdD	0.00799846	0.135	0.6778591	-112.6454	-113.232	0.2787568
ttlmassD	0.0254584	0.02	0.9586421	-144.0437	-147.1176	0.01315614
rmrD	0.0117638	0.1	0.8587116	-71.60054	-74.00027	0.02846858
WUE_D	0.0206935	0.023	0.5455282	-9.438273	-9.102205	1

**leafcD**	**0.00564166**	**0.206**	**6.61E-05**	**-86.81046**	**-86.80988**	**1**
**leafnD**	**0.0127895**	**0.066**	**0.8379002**	**-52.22653**	**-57.04403**	**0.001909058**
**cnD**	**0.015233**	**0.051**	**0.8350859**	**-24.49639**	**-28.06983**	**0.007509447**

**Climate trait**	**K**	**p-value**	**lambda(ʎ)**	**logL(ʎ)**	**LR(ʎ=0)**	**p-value**
**PCA1**	**0.462712**	**0.005**	**0.753963**	**-72.2802**	**9.38246**	**0.00219071**

Figure S1

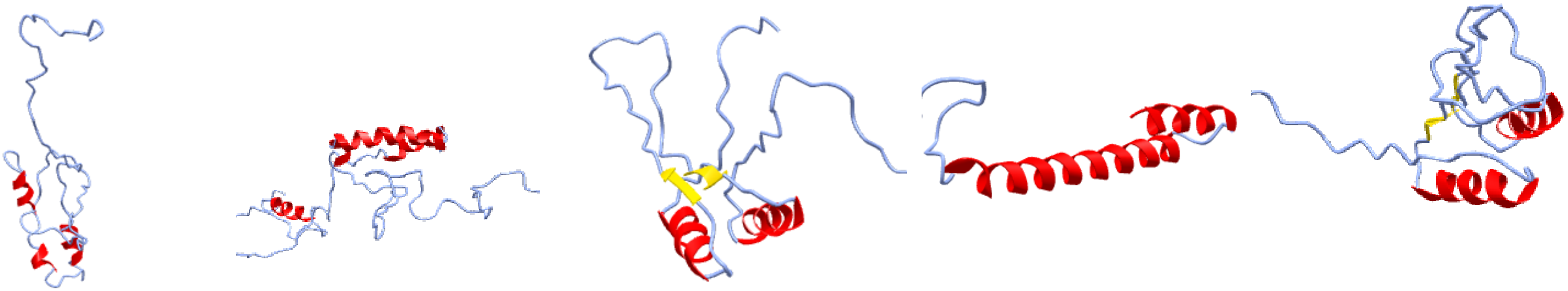

(A) Bdhn4 (B) Bdhn3 (C) Bdhn1 (D) Bdhn10 (E) BdhnBsyl1b

Complete sets of 3D structures for all *Brachypodium* species and proteins are available in the following links:

Brachypodium distachyon: http://zeta.uma.es/public/journal/brachy/raptorx/distachyon.html
Brachypodium hybridum D: http://zeta.uma.es/public/journal/brachy/raptorx/hybridumD.html
Brachypodium stacei: http://zeta.uma.es/public/journal/brachy/raptorx/stacei.html
Brachypodium hybridum S: http://zeta.uma.es/public/journal/brachy/raptorx/hybridumS.html
Brachypodium sylvaticum: http://zeta.uma.es/public/journal/brachy/raptorx/sylvaticum.html

Figure S2

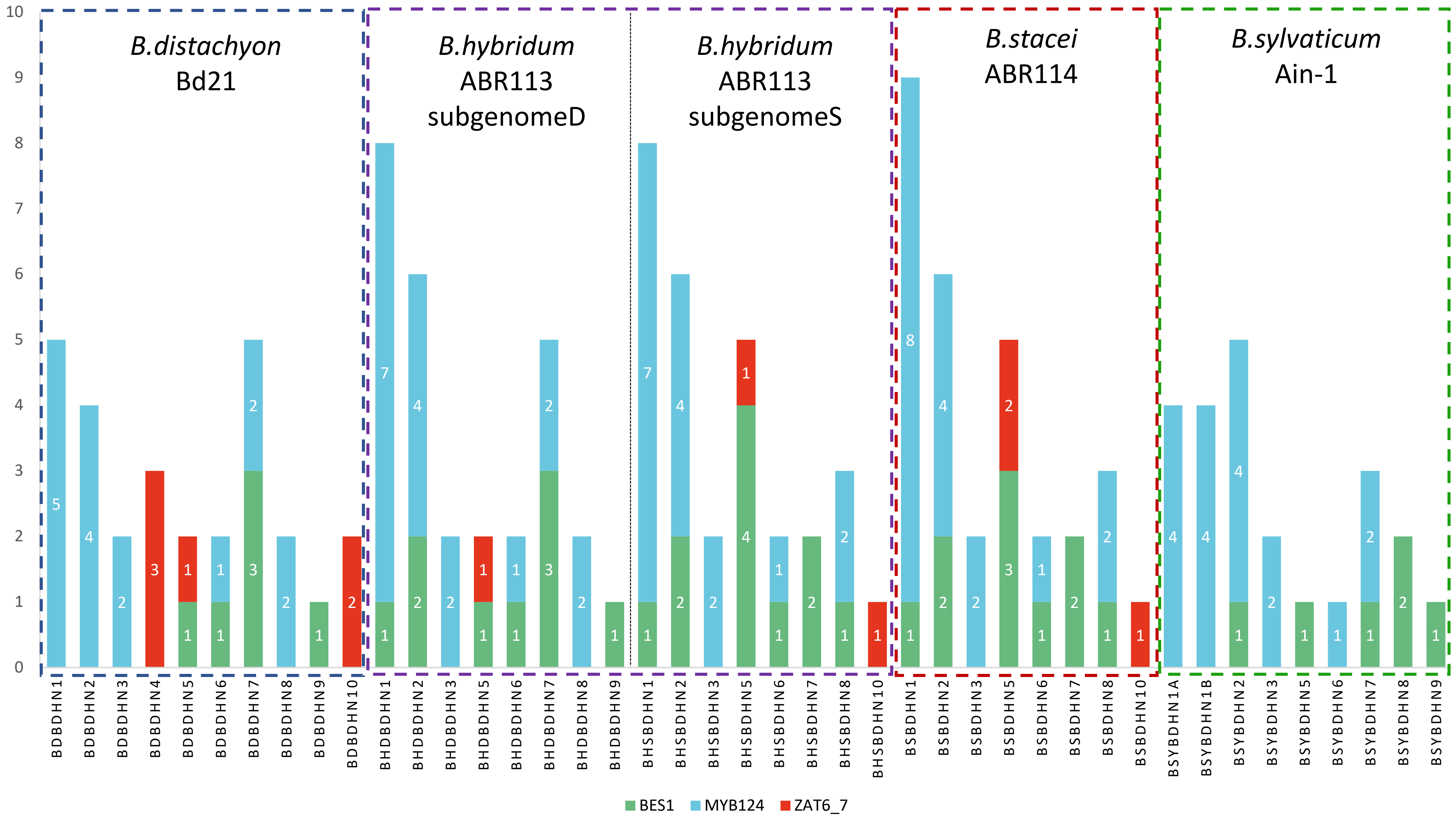
B.distachyon
Bd21
B.hybridum
ABR113
subgenomeD
B.hybridum
ABR113
subgenomeS
B.stacei
ABR114
B.sylvaticum
Ain-1
BDBDHN1
BDBDHN2
BDBDHN3
BDBDHN4
BDBDHN5
BDBDHN6
BDBDHN7
BDBDHN8
BDBDHN9
BDBDHN10
BHDBDHN1
BHDBDHN2
BHDBDHN3
BHDBDHN5
BHDBDHN6
BHDBDHN7
BHDBDHN8
BHDBDHN9
BHSBDHN1
BHSBDHN2
BHSBDHN3
BHSBDHN5
BHSBDHN6
BHSBDHN7
BHSBDHN8
BHSBDHN10
BSBDHN1
BSBDHN2
BSBDHN3
BSBDHN5
BSBDHN6
BSBDHN7
BSBDHN8
BSBDHN10
BSYBDHN1A
BSYBDHN1B
BSYBDHN2
BSYBDHN3
BSYBDHN5
BSYBDHN6
BSYBDHN7
BSYBDHN8
BSYBDHN9
BES1
MYB124
ZAT6_7

Figure S2

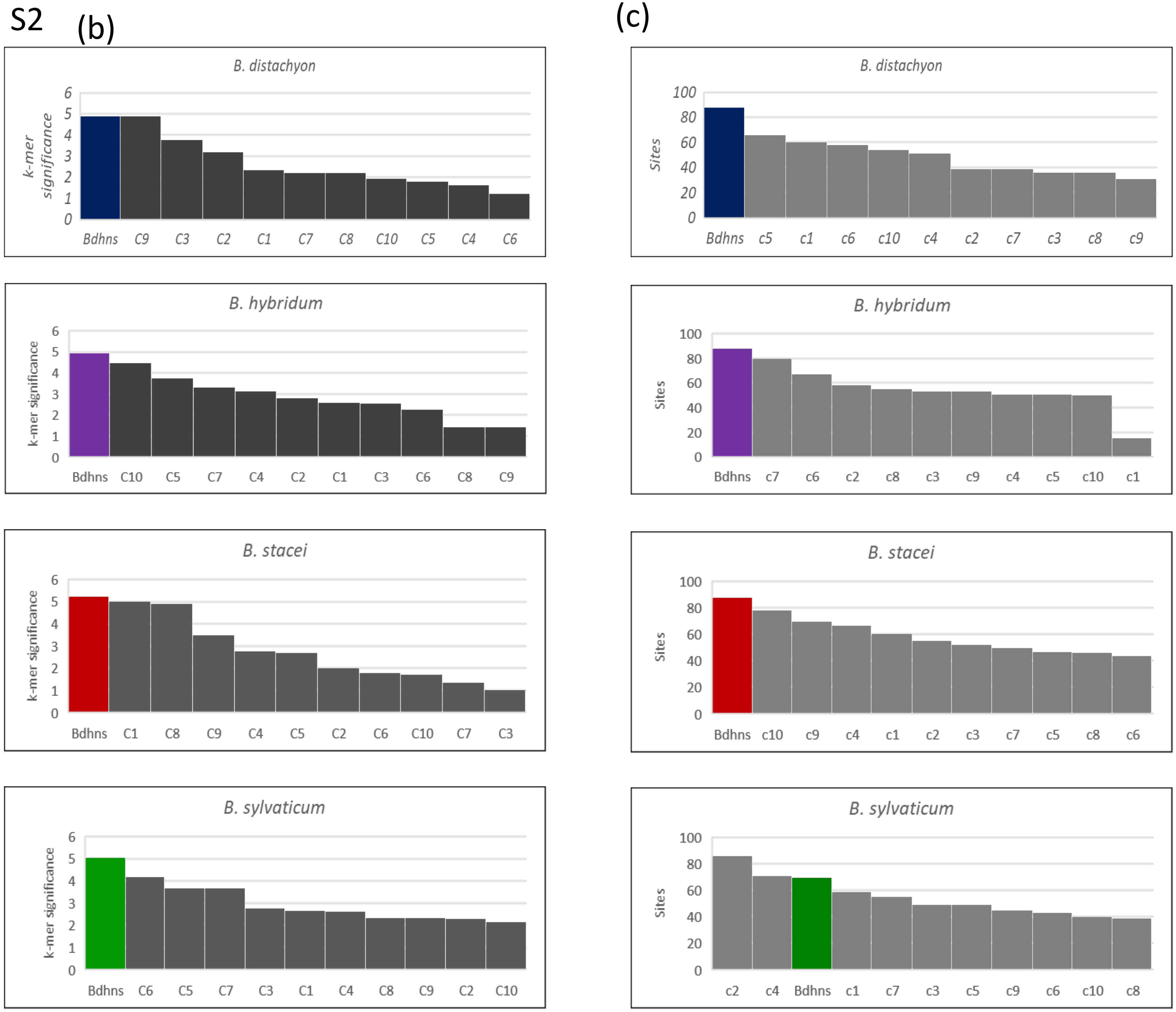

(b)
B. distachyon
k-mer significance
Bdhns C9 C3 C2 C1 C7 C8 C10 C5 C4 C6
B. hybridum
k-mer significance
Bdhns C10 C5 C7 C4 C2 C1 C3 C6 C8 C9
B. stacei
k-mer significance
Bdhns C1 C8 C9 C4 C5 C2 C6 C10 C7 C3
B. sylvaticum
k-mer significance
Bdhns C6 C5 C7 C3 C1 C4 C8 C9 C2 C10
(c)
B. distachyon
Sites
Bdhns c5 c1 c6 c10 c4 c2 c7 c3 c8 c9
B. hybridum
Sites
Bdhns c7 c6 c2 c8 c3 c9 c4 c5 c10 c1
B. stacei
Sites
Bdhns c10 c9 c4 c1 c2 c3 c7 c5 c8 c6
B. sylvaticum
Sites
c2 c4 Bdhns c1 c7 c3 c5 c9 c6 c10 c8

Figure S3

Shape	Meaning
□	Annotated as PF0257
△	Only similar to other specie's dehydrin
◇	Manually found
○	Belongs to an unknown chromosome

DHN Colour	B. distachyon DHN	Bd1	Bd2	Bd3	Bd4	Bd5
	Bdhn1				2	51
	Bdhn2			20	34	
	Bdhn3	54				
	Bdhn4			27	19	
	Bdhn5			34	19	
	Bdhn6			6	44	2
	Bdhn7			24	30	
	Bdhn8			22	33	
	Bdhn9		49			10
	Bdhn10	52			2	

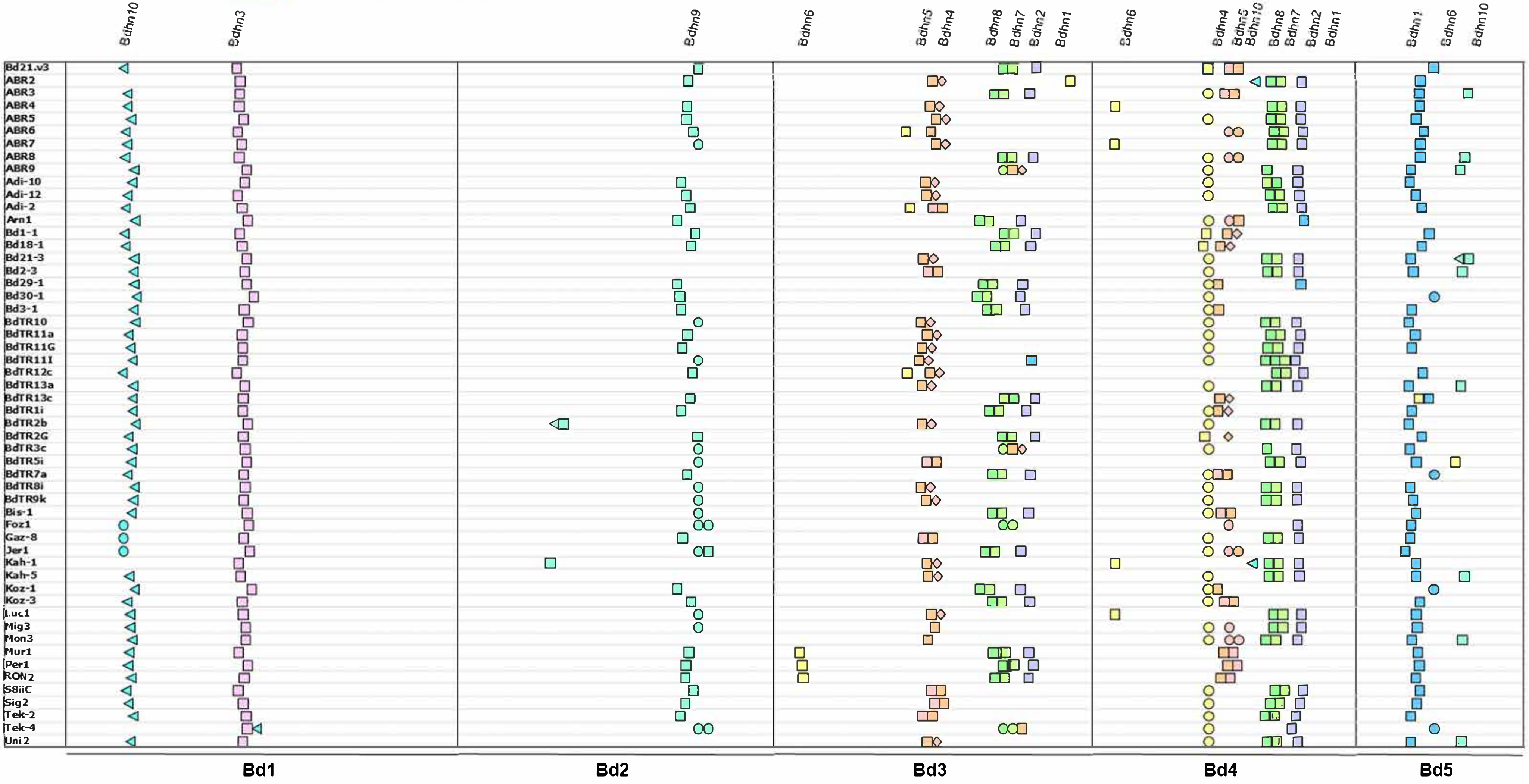

Figure S4

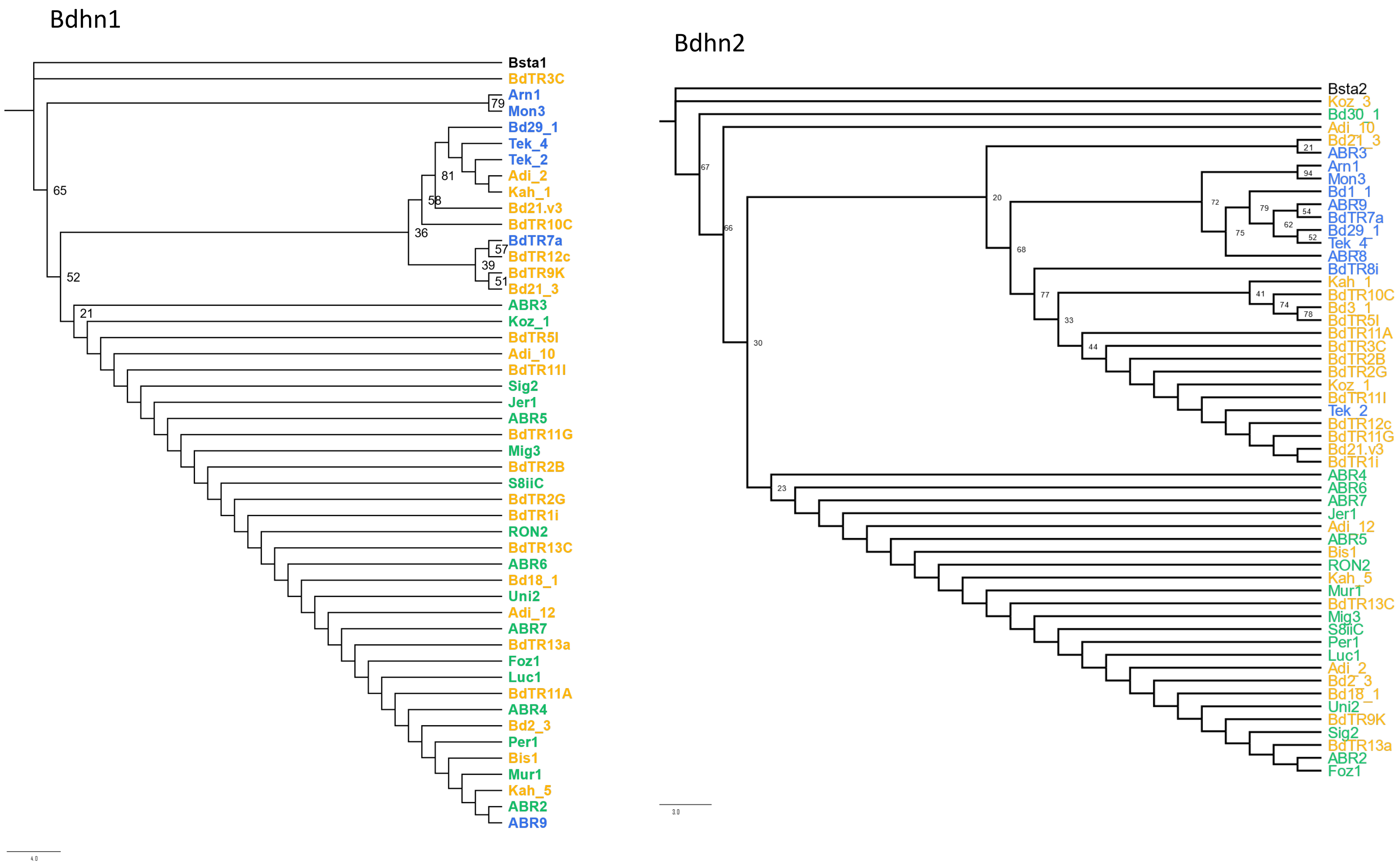

Bdhn1
Bsta1
BdTR3C
Arn1
Mon3
Bd29_1
Tek_4
Tek_2
Adi_2
Kah_1
Bd21.v3
BdTR10C
BdTR7a
BdTR12c
BdTR9K
Bd21_3
ABR3
Koz_1
BdTR5I
Adi_10
BdTR11I
Sig2
Jer1
ABR5
BdTR11G
Mig3
BdTR2B
S8iiC
BdTR2G
BdTR1i
RON2
BdTR13C
ABR6
Bd18_1
Uni2
Adi_12
ABR7
BdTR13a
Foz1
Luc1
BdTR11A
ABR4
Bd2_3
Per1
Bis1
Mur1
Kah_5
ABR2
ABR9
79
81
58
36
57
39
51
65
52
21
4.0
Bdhn2
Bsta2
Koz_3
Bd30_1
Adi_10
Bd21_3
ABR3
Arn1
Mon3
Bd1_1
ABR9
BdTR7a
Bd29_1
Tek_4
ABR8
BdTR8i
Kah_1
BdTR10C
Bd3_1
BdTR5I
BdTR11A
BdTR3C
BdTR2B
BdTR2G
Koz_1
BdTR11I
Tek_2
BdTR12c
BdTR11G
Bd21.v3
BdTR1i
ABR4
ABR6
ABR7
Jer1
Adi_12
ABR5
Bis1
RON2
Kah_5
Mur1
BdTR13C
Mig3
S8iiC
Per1
Luc1
Adi_2
Bd2_3
Bd18_1
Uni2
BdTR9K
Sig2
BdTR13a
ABR2
Foz1
67
66
30
21
94
20
72
79
54
62
52
75
68
77
41
74
78
33
44
23
3.0

Figure S4

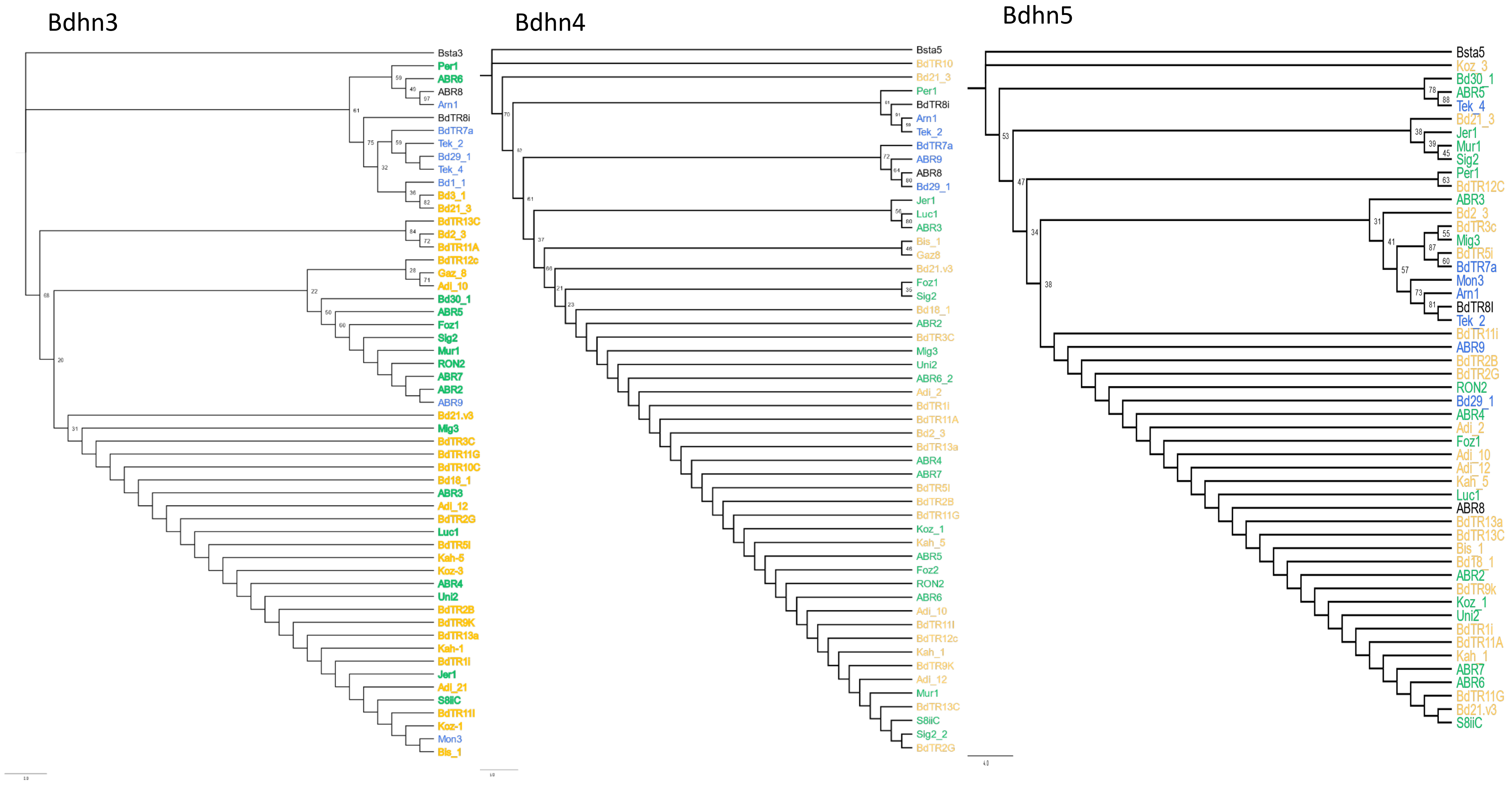
Bdhn3
Bsta3
Per1
ABR6
ABR8
Arn1
BdTR8i
BdTR7a
Tek_2
Bd29_1
Tek_4
Bd1_1
Bd3_1
Bd21_3
BdTR13C
Bd2_3
BdTR11A
BdTR12c
Gaz_8
Adi_10
Bd30_1
ABR5
Foz1
Sig2
Mur1
RON2
ABR7
ABR2
ABR9
Bd21.v3
Mig3
BdTR3C
BdTR11G
BdTR10C
Bd18_1
ABR3
Adi_12
BdTR2G
Luc1
BdTR5I
Kah-5
Koz-3
ABR4
Uni2
BdTR2B
BdTR9K
BdTR13a
Kah-1
BdTR1I
Jer1
Adi_21
S8iiC
BdTR11I
Koz-1
Mon3
Bis_1
Bdhn4
Bsta5
BdTR10
Bd21_3
Per1
BdTR8i
Arn1
Tek_2
BdTR7a
ABR9
ABR8
Bd29_1
Jer1
Luc1
ABR3
Bis_1
Gaz8
Bd21.v3
Foz1
Sig2
Bd18_1
ABR2
BdTR3C
Mig3
Uni2
ABR6_2
Adi_2
BdTR1I
BdTR11A
Bd2_3
BdTR13a
ABR4
ABR7
BdTR5I
BdTR2B
BdTR11G
Koz_1
Kah_5
ABR5
Foz2
RON2
ABR6
Adi_10
BdTR11I
BdTR12c
Kah_1
BdTR9K
Adi_12
Mur1
BdTR13C
S8iiC
Sig2_2
BdTR2G
Bdhn5
Bsta5
Koz_3
Bd30_1
ABR5
Tek_4
Bd21_3
Jer1
Mur1
Sig2
Per1
BdTR12C
ABR3
Bd2_3
BdTR3c
Mig3
BdTR5i
BdTR7a
Mon3
Arn1
BdTR8I
Tek_2
BdTR11i
ABR9
BdTR2B
BdTR2G
RON2
Bd29_1
ABR4
Adi_2
Foz1
Adi_10
Adi_12
Kah_5
Luc1
ABR8
BdTR13a
BdTR13C
Bis_1
Bd18_1
ABR2
BdTR9k
Koz_1
Uni2
BdTR1i
BdTR11A
Kah_1
ABR7
ABR6
BdTR11G
Bd21.v3
S8iiC

Figure S4

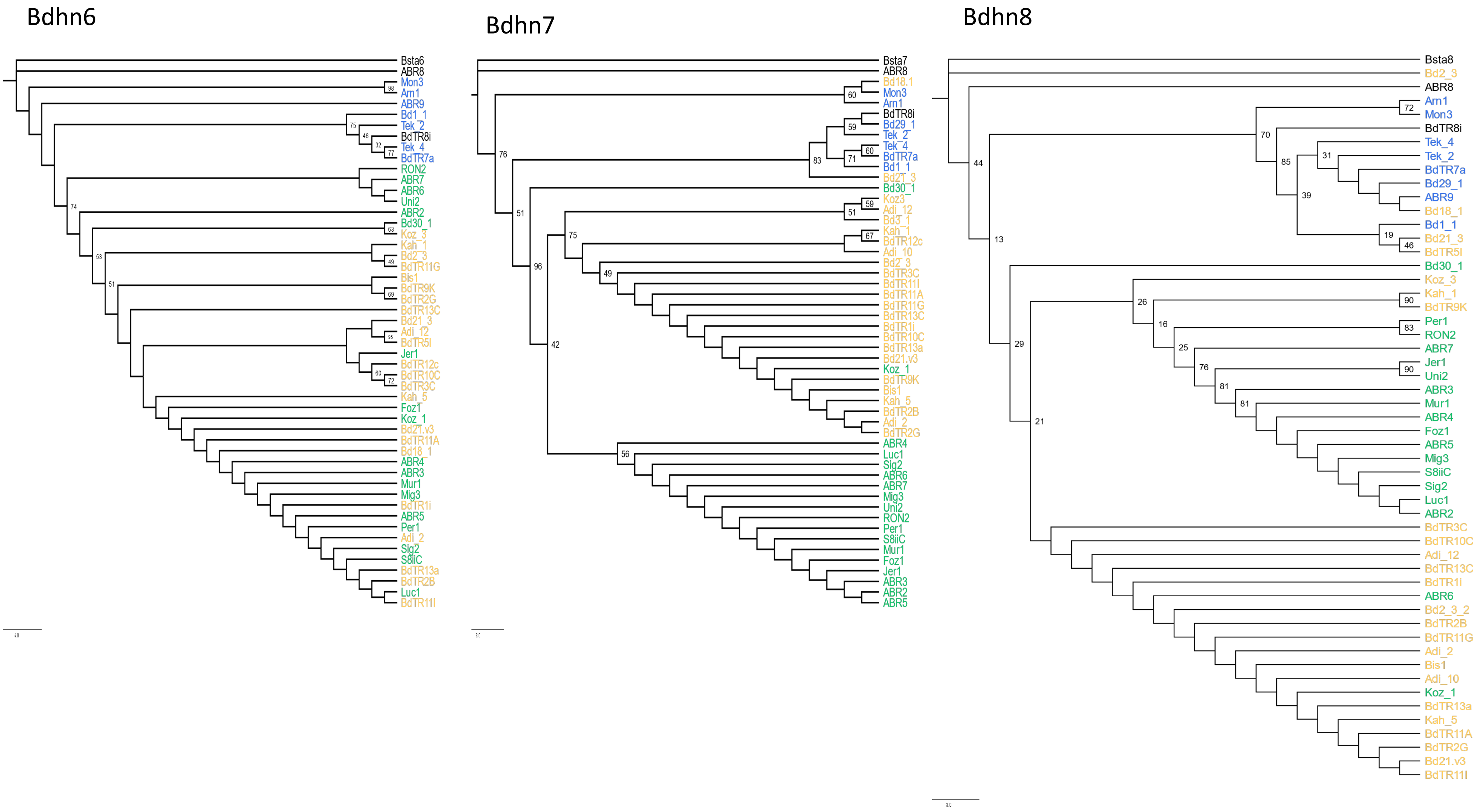
Bdhn6
Bsta6
ABR8
Mon3
Arn1
ABR9
Bd1_1
Tek_2
BdTR8i
Tek_4
BdTR7a
RON2
ABR7
ABR6
Uni2
ABR2
Bd30_1
Koz_3
Kah_1
Bd2_3
BdTR11G
Bis1
BdTR9K
BdTR2G
BdTR13C
Bd21_3
Adi_12
BdTR5I
Jer1
BdTR12c
BdTR10C
BdTR3C
Kah_5
Foz1
Koz_1
Bd21.v3
BdTR11A
Bd18_1
ABR4
ABR3
Mur1
Mig3
BdTR1i
ABR5
Per1
Adi_2
Sig2
S8iiC
BdTR13a
BdTR2B
Luc1
BdTR11I
Bdhn7
Bsta7
ABR8
Bd18.1
Mon3
Arn1
BdTR8i
Bd29_1
Tek_2
Tek_4
BdTR7a
Bd1_1
Bd21_3
Bd30_1
Koz3
Adi_12
Bd3_1
Kah_1
BdTR12c
Adi_10
Bd2_3
BdTR3C
BdTR11I
BdTR11A
BdTR11G
BdTR13C
BdTR1i
BdTR10C
BdTR13a
Bd21.v3
Koz_1
BdTR9K
Bis1
Kah_5
BdTR2B
Adi_2
BdTR2G
ABR4
Luc1
Sig2
ABR6
ABR7
Mig3
Uni2
RON2
Per1
S8iiC
Mur1
Foz1
Jer1
ABR3
ABR2
ABR5
Bdhn8
Bsta8
Bd2_3
ABR8
Arn1
Mon3
BdTR8i
Tek_4
Tek_2
BdTR7a
Bd29_1
ABR9
Bd18_1
Bd1_1
Bd21_3
BdTR5I
Bd30_1
Koz_3
Kah_1
BdTR9K
Per1
RON2
ABR7
Jer1
Uni2
ABR3
Mur1
ABR4
Foz1
ABR5
Mig3
S8iiC
Sig2
Luc1
ABR2
BdTR3C
BdTR10C
Adi_12
BdTR13C
BdTR1I
ABR6
Bd2_3_2
BdTR2B
BdTR11G
Adi_2
Bis1
Adi_10
Koz_1
BdTR13a
Kah_5
BdTR11A
BdTR2G
Bd21.v3
BdTR11I

Figure S4

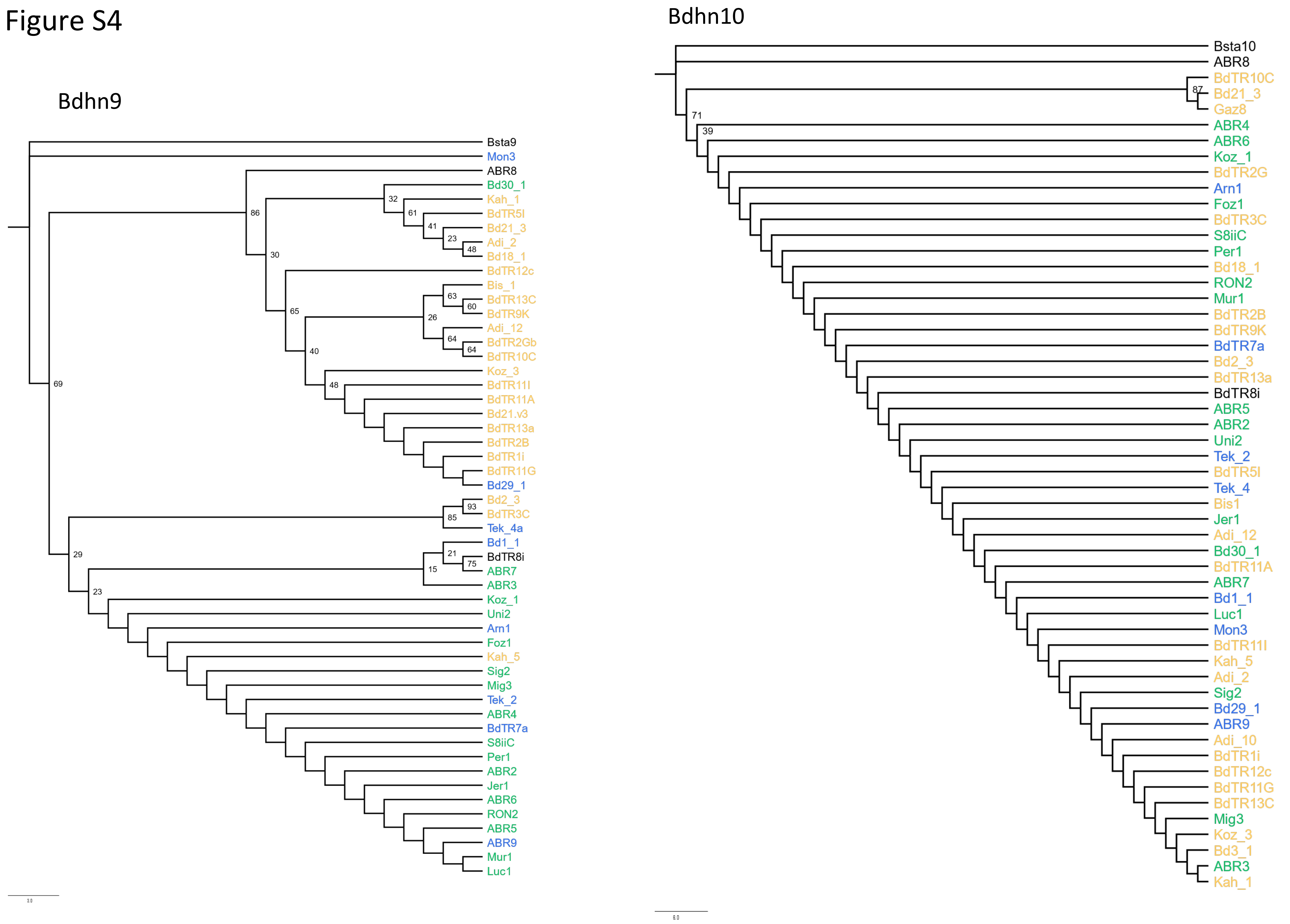
Bdhn9
Bsta9
Mon3
ABR8
Bd30_1
Kah_1
BdTR5I
Bd21_3
Adi_2
Bd18_1
BdTR12c
Bis_1
BdTR13C
BdTR9K
Adi_12
BdTR2Gb
BdTR10C
Koz_3
BdTR11I
BdTR11A
Bd21.v3
BdTR13a
BdTR2B
BdTR1i
BdTR11G
Bd29_1
Bd2_3
BdTR3C
Tek_4a
Bd1_1
BdTR8i
ABR7
ABR3
Koz_1
Uni2
Arn1
Foz1
Kah_5
Sig2
Mig3
Tek_2
ABR4
BdTR7a
S8iiC
Per1
ABR2
Jer1
ABR6
RON2
ABR5
ABR9
Mur1
Luc1
Bdhn10
Bsta10
ABR8
BdTR10C
Bd21_3
Gaz8
ABR4
ABR6
Koz_1
BdTR2G
Arn1
Foz1
BdTR3C
S8iiC
Per1
Bd18_1
RON2
Mur1
BdTR2B
BdTR9K
BdTR7a
Bd2_3
BdTR13a
BdTR8i
ABR5
ABR2
Uni2
Tek_2
BdTR5I
Tek_4
Bis1
Jer1
Adi_12
Bd30_1
BdTR11A
ABR7
Bd1_1
Luc1
Mon3
BdTR11I
Kah_5
Adi_2
Sig2
Bd29_1
ABR9
Adi_10
BdTR1i
BdTR12c
BdTR11G
BdTR13C
Mig3
Koz_3
Bd3_1
ABR3
Kah_1

Figure S5

#	Ecotypes	PCA1
1	ABR2	2.579269
2	ABR3	4.86108471
3	ABR4	5.69006454
4	ABR5	5.39529274
5	ABR6	3.40042965
6	ABR8	2.17586982
7	Adi10	-1.75668741
8	Adi12	-1.75668741
9	Adi2	-1.75668741
10	Bd1-1	-0.5973436
11	Bd18-1	0.73085546
12	Bd2-3	-5.71768222
13	Bd21-3	-3.51663046
14	Bd21control	-3.51663046
15	Bd3-1	-3.51663046
16	Bd30-1	-0.45762855
17	BdTR10c	0.88782452
18	BdTR11g	1.03334719
19	BdTR11i	0.04350879
20	BdTR1i	-0.28516673
21	BdTR2b	1.56035254
22	BdTR2g	2.60379124
23	BdTR5i	2.60379124
24	BdTR9k	1.12002182
25	Bis1	-3.66257669
26	Kah1	-3.54231265
27	Kah5	-3.54231265
28	Koz1	-3.2693441
29	Koz3	-3.2693441
30	RON2	5.47816162

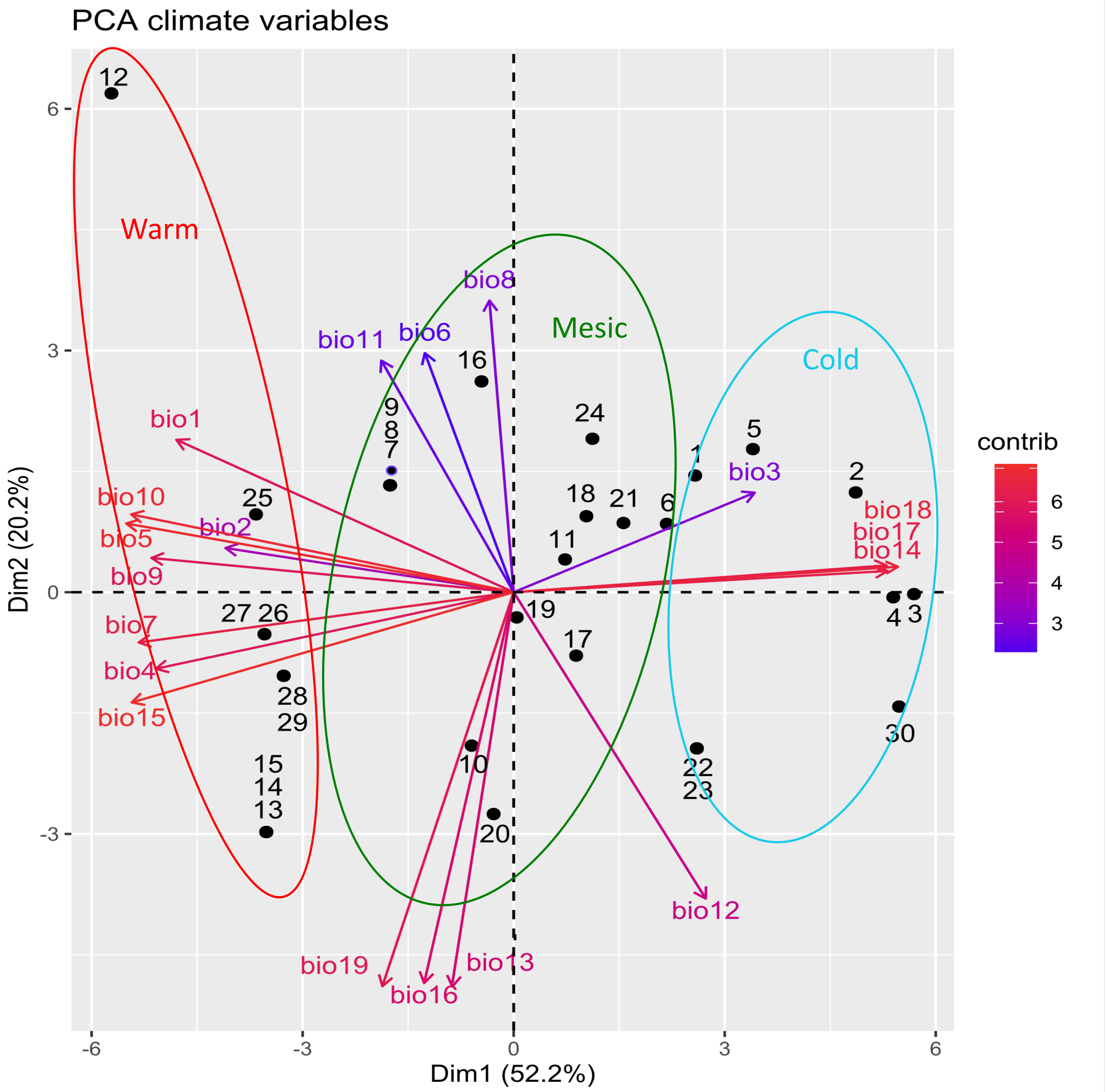

Figure S6

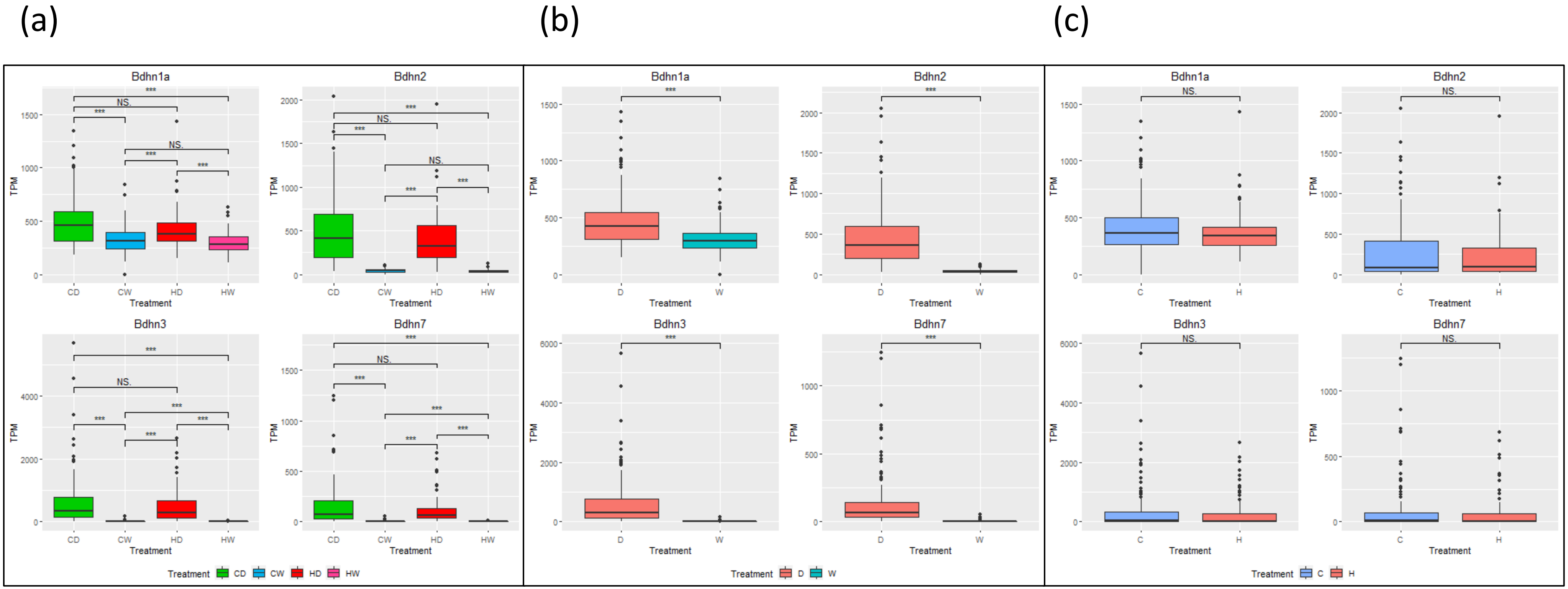
(a)
(b)
(c)
Bdhn1a
Bdhn2
Bdhn3
Bdhn7
TPM
Treatment
CD
CW
HD
HW
D
W
C
H
NS.
***
Treatment CD CW HD HW
Treatment D W
Treatment C H

Figure S7

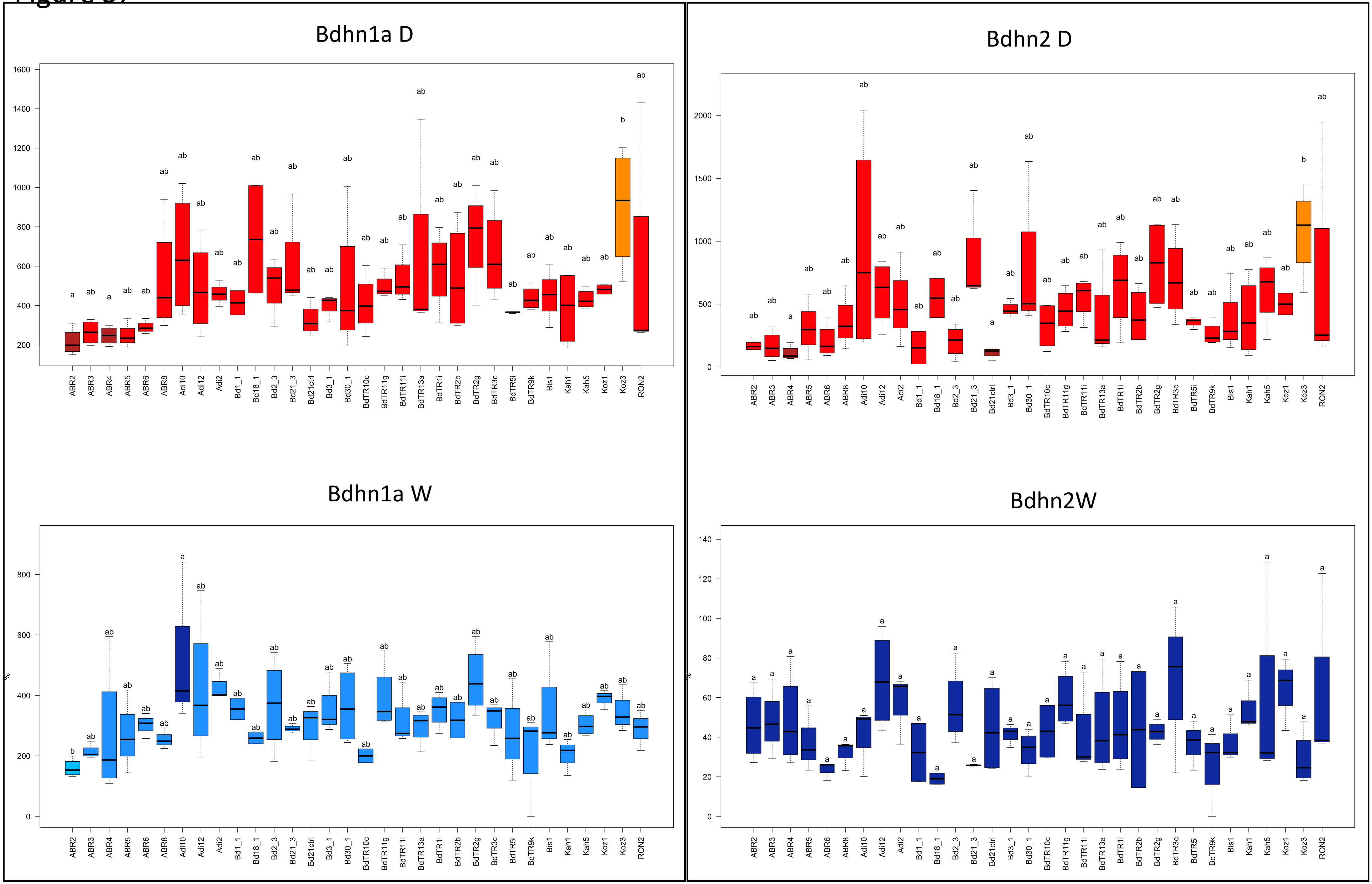

Bdhn1a D
1600
1400
1200
1000
800
600
400
200
ABR2 ABR3 ABR4 ABR5 ABR6 ABR8 Adi10 Adi12 Adi2 Bd1_1 Bd18_1 Bd2_3 Bd21_3 Bd21ctrl Bd3_1 Bd30_1 BdTR10c BdTR11g BdTR11i BdTR13a BdTR1i BdTR2b BdTR2g BdTR3c BdTR5i BdTR9k Bis1 Kah1 Kah5 Koz1 Koz3 RON2
Bdhn2 D
2000
1500
1000
500
0
ABR2 ABR3 ABR4 ABR5 ABR6 ABR8 Adi10 Adi12 Adi2 Bd1_1 Bd18_1 Bd2_3 Bd21_3 Bd21ctrl Bd3_1 Bd30_1 BdTR10c BdTR11g BdTR11i BdTR13a BdTR1i BdTR2b BdTR2g BdTR3c BdTR5i BdTR9k Bis1 Kah1 Kah5 Koz1 Koz3 RON2
Bdhn1a W
800
600
400
200
0
%
ABR2 ABR3 ABR4 ABR5 ABR6 ABR8 Adi10 Adi12 Adi2 Bd1_1 Bd18_1 Bd2_3 Bd21_3 Bd21ctrl Bd3_1 Bd30_1 BdTR10c BdTR11g BdTR11i BdTR13a BdTR1i BdTR2b BdTR2g BdTR3c BdTR5i BdTR9k Bis1 Kah1 Kah5 Koz1 Koz3 RON2
Bdhn2W
140
120
100
80
60
40
20
0
%
ABR2 ABR3 ABR4 ABR5 ABR6 ABR8 Adi10 Adi12 Adi2 Bd1_1 Bd18_1 Bd2_3 Bd21_3 Bd21ctrl Bd3_1 Bd30_1 BdTR10c BdTR11g BdTR11i BdTR13a BdTR1i BdTR2b BdTR2g BdTR3c BdTR5i BdTR9k Bis1 Kah1 Kah5 Koz1 Koz3 RON2

Figure S7

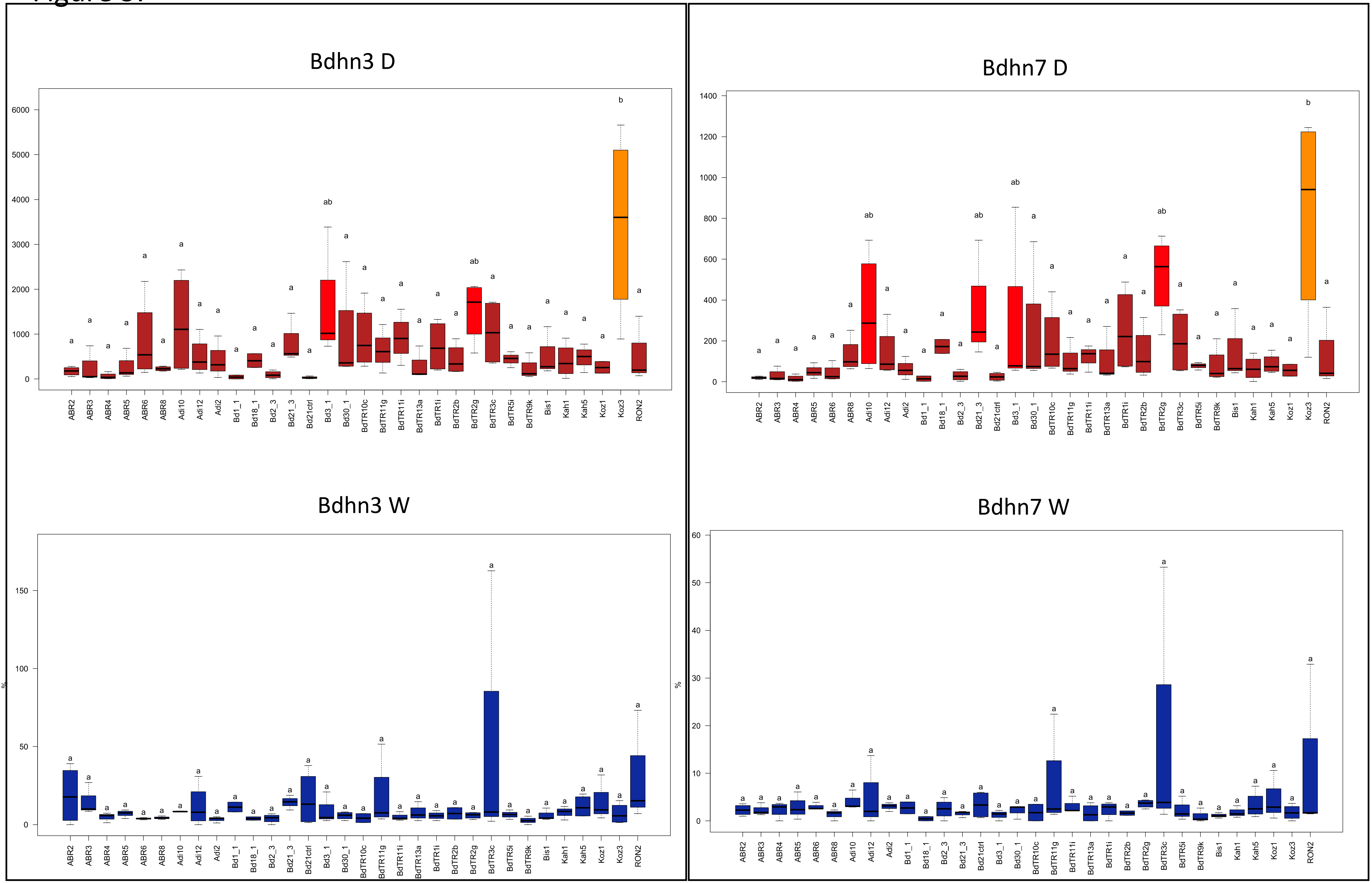

Bdhn3 D
Bdhn7 D
Bdhn3 W
Bdhn7 W
%
ABR2
ABR3
ABR4
ABR5
ABR6
ABR8
Adi10
Adi12
Adi2
Bd1_1
Bd18_1
Bd2_3
Bd21_3
Bd21ctrl
Bd3_1
Bd30_1
BdTR10c
BdTR11g
BdTR11i
BdTR13a
BdTR1i
BdTR2b
BdTR2g
BdTR3c
BdTR5i
BdTR9k
Bis1
Kah1
Kah5
Koz1
Koz3
RON2

Figure S8

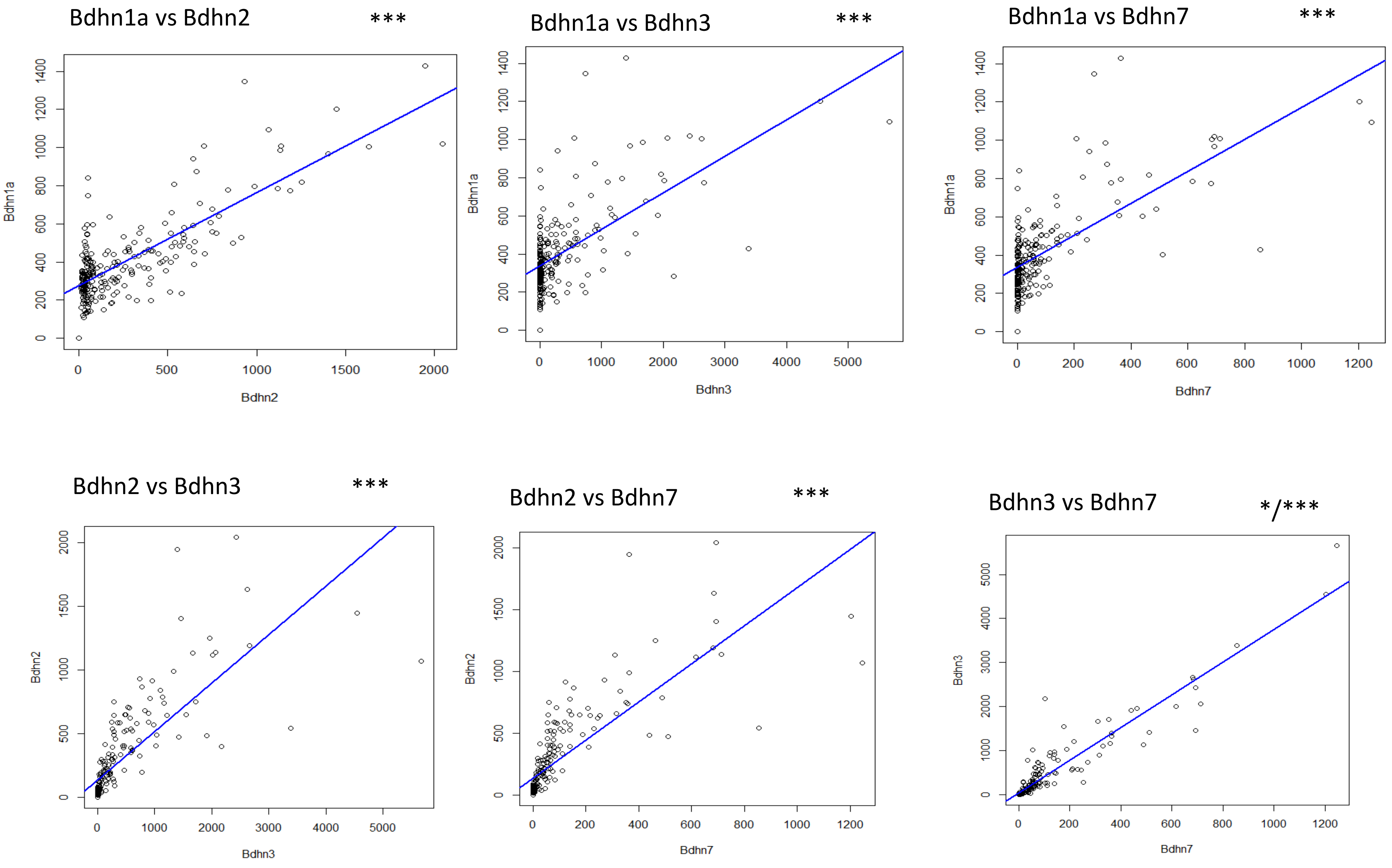

Bdhn1a vs Bdhn2 ***
Bdhn1a
Bdhn2
0 200 400 600 800 1000 1200 1400
0 500 1000 1500 2000
Bdhn1a vs Bdhn3 ***
Bdhn1a
Bdhn3
0 200 400 600 800 1000 1200 1400
0 1000 2000 3000 4000 5000
Bdhn1a vs Bdhn7 ***
Bdhn1a
Bdhn7
0 200 400 600 800 1000 1200 1400
0 200 400 600 800 1000 1200
Bdhn2 vs Bdhn3 ***
Bdhn2
Bdhn3
0 500 1000 1500 2000
0 1000 2000 3000 4000 5000
Bdhn2 vs Bdhn7 ***
Bdhn2
Bdhn7
0 500 1000 1500 2000
0 200 400 600 800 1000 1200
Bdhn3 vs Bdhn7 */***
Bdhn3
Bdhn7
0 1000 2000 3000 4000 5000
0 200 400 600 800 1000 1200

Figure S9

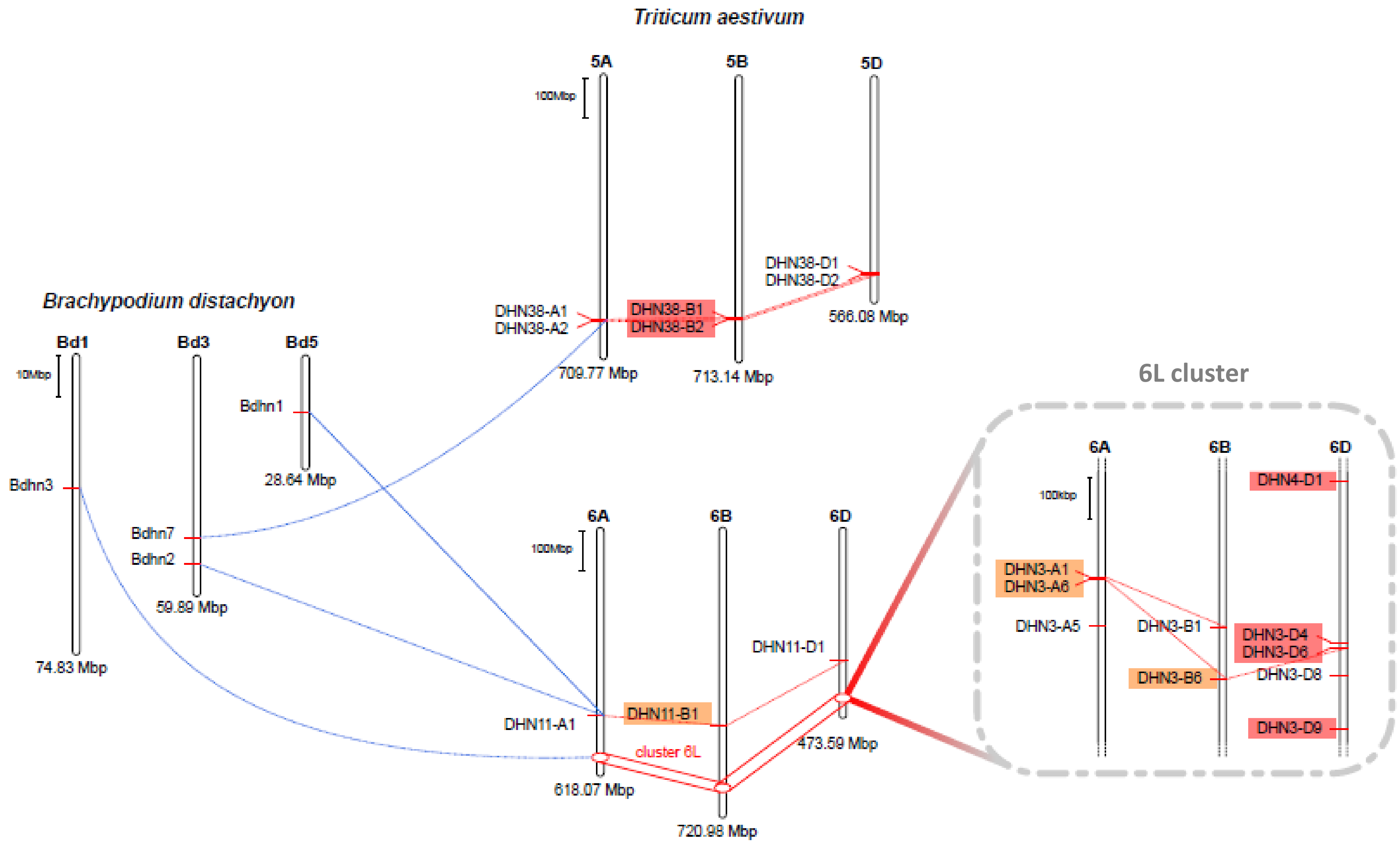

Triticum aestivum
5A
5B
5D
100Mbp
DHN38-D1
DHN38-D2
566.08 Mbp
DHN38-A1
DHN38-A2
DHN38-B1
DHN38-B2
709.77 Mbp
713.14 Mbp
Brachypodium distachyon
Bd1
Bd3
Bd5
10Mbp
Bdhn1
28.64 Mbp
Bdhn3
Bdhn7
Bdhn2
59.89 Mbp
74.83 Mbp
6L cluster
6A
6B
6D
100Mbp
DHN11-D1
DHN11-B1
DHN11-A1
cluster 6L
473.59 Mbp
618.07 Mbp
720.98 Mbp
100kbp
DHN4-D1
DHN3-A1
DHN3-A6
DHN3-A5
DHN3-B1
DHN3-D4
DHN3-D6
DHN3-B6
DHN3-D8
DHN3-D9

Figure S10

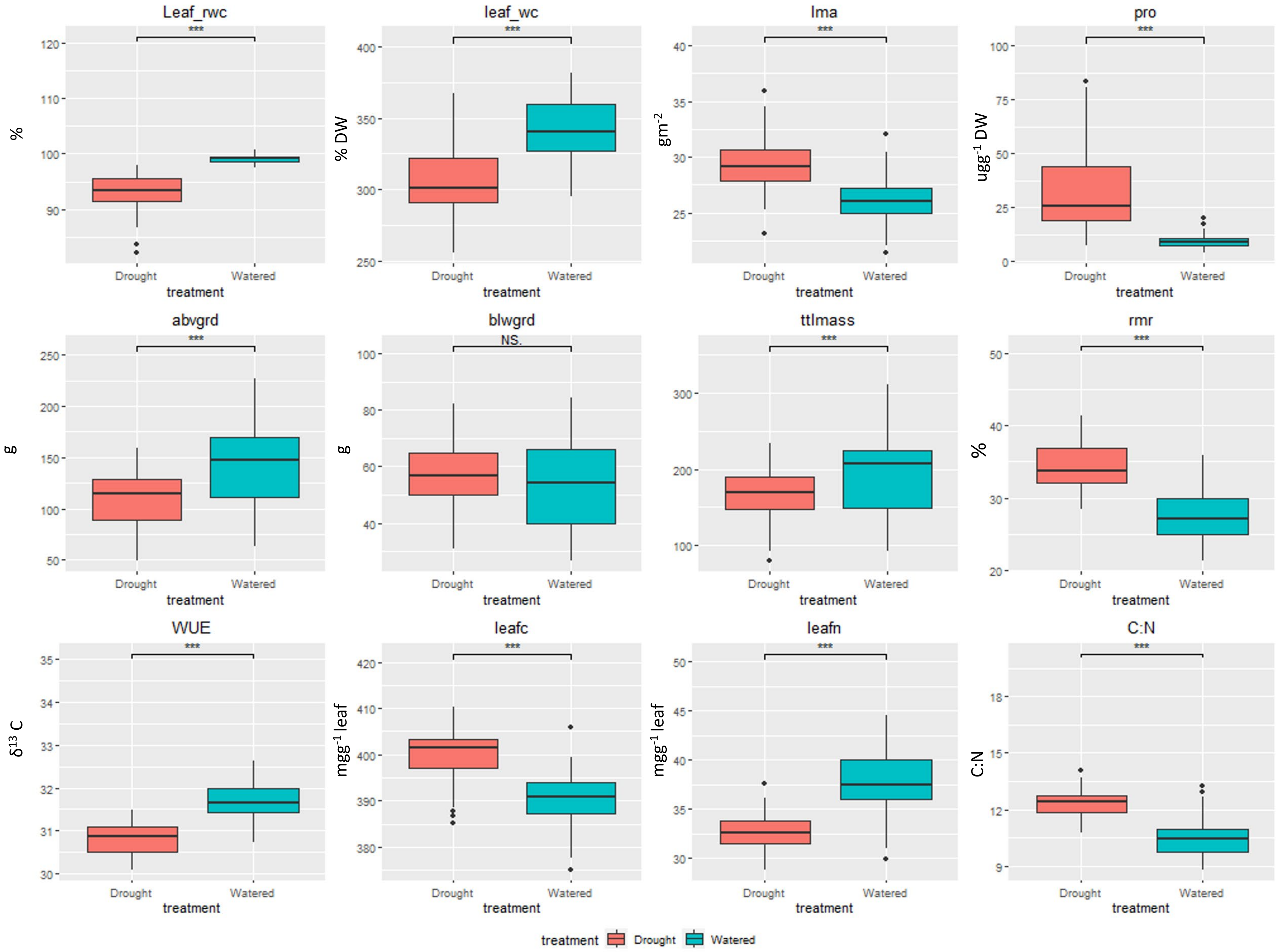

Leaf_rwc
leaf_wc
lma
pro
abvgrd
blwgrd
ttlmass
rmr
WUE
leafc
leafn
C:N
***
NS.
%
% DW
gm-2
ugg-1 DW
g
δ13 C
mgg-1 leaf
C:N
Drought
Watered
treatment
treatment Drought Watered

Figure S11

Bdhn1a vs phenotypic traits

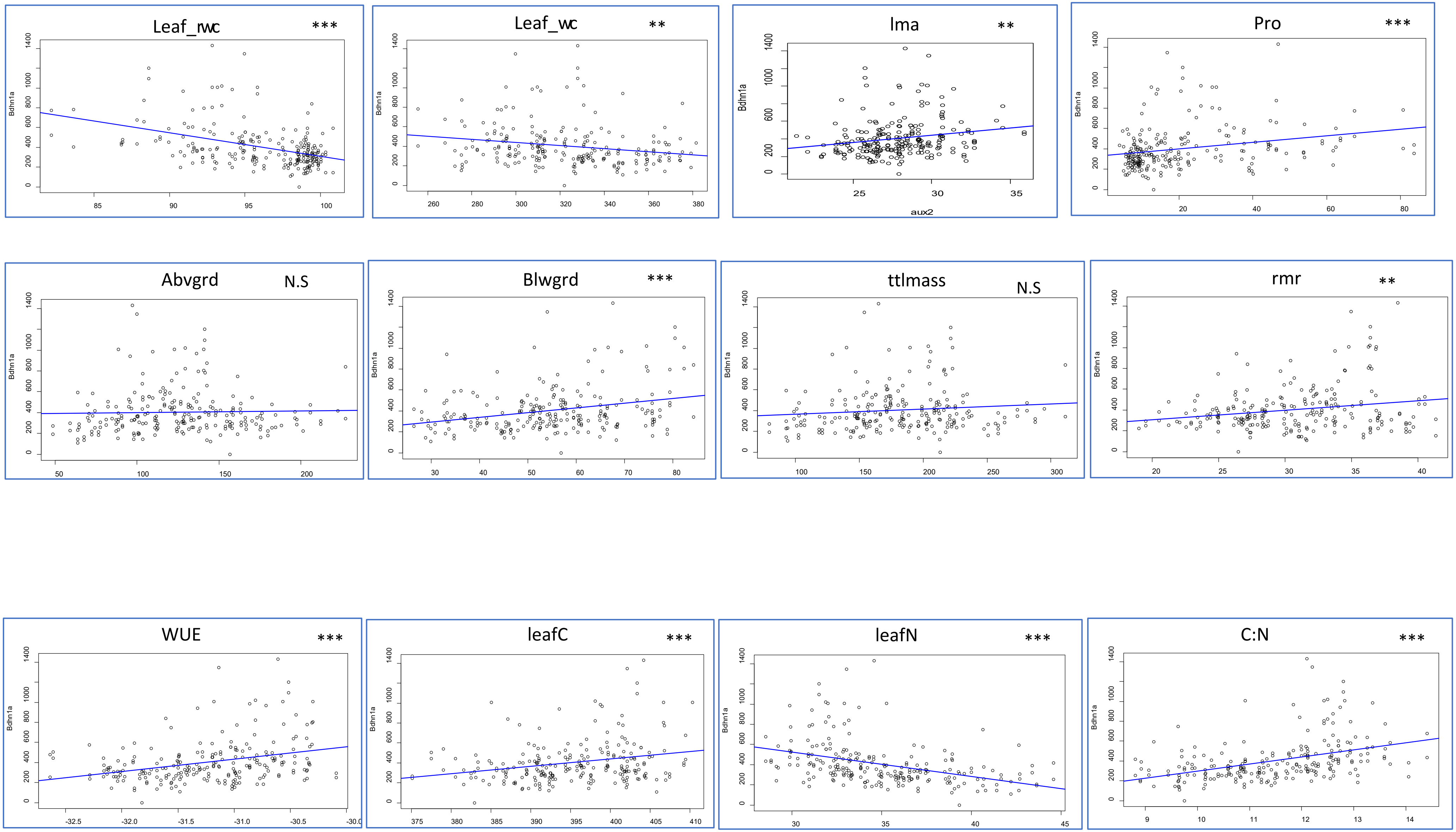

Figure S11

Bdhn2 vs phenotypic traits

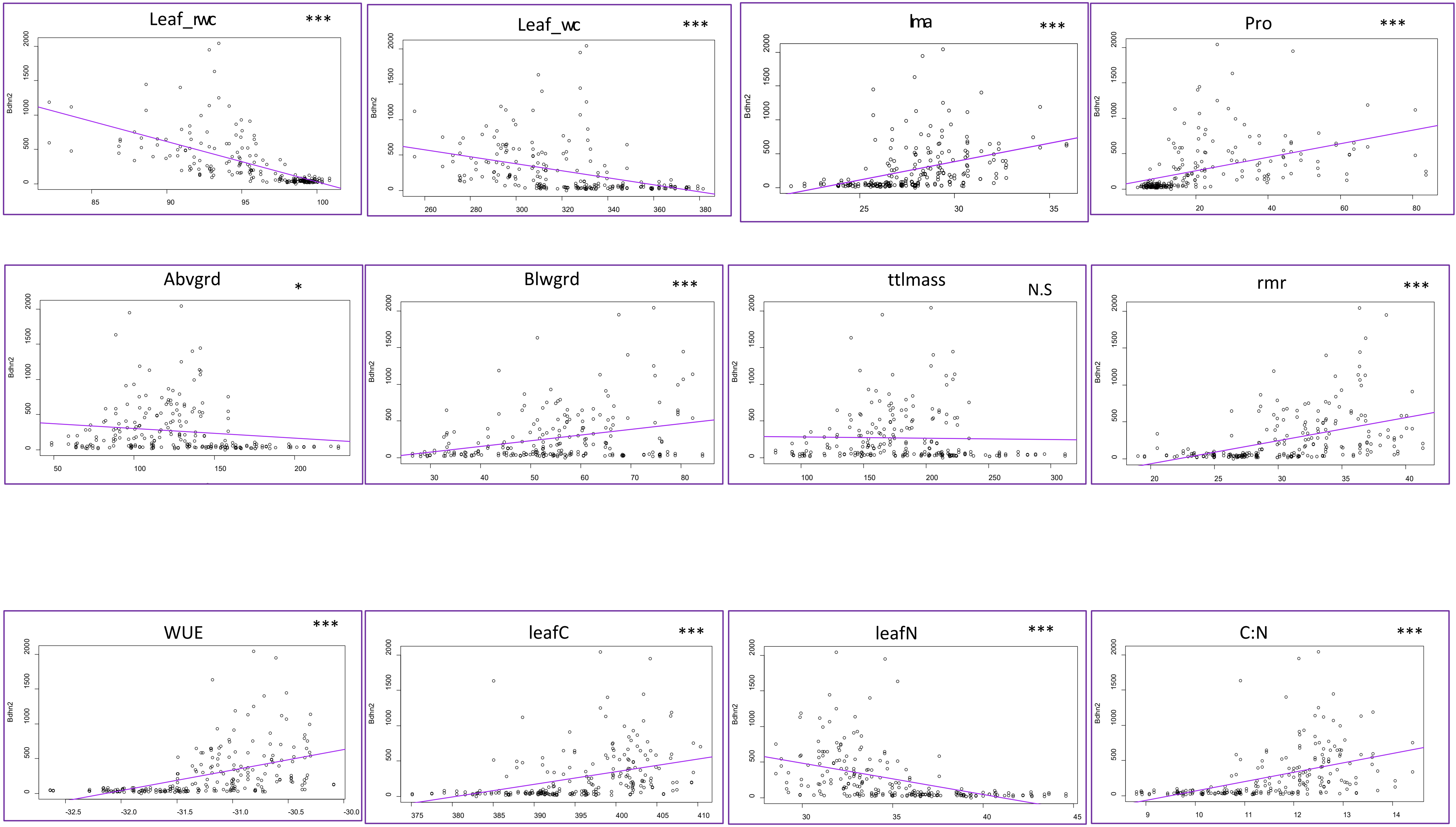

Figure S11

Bdhn3vs phenotypic traits

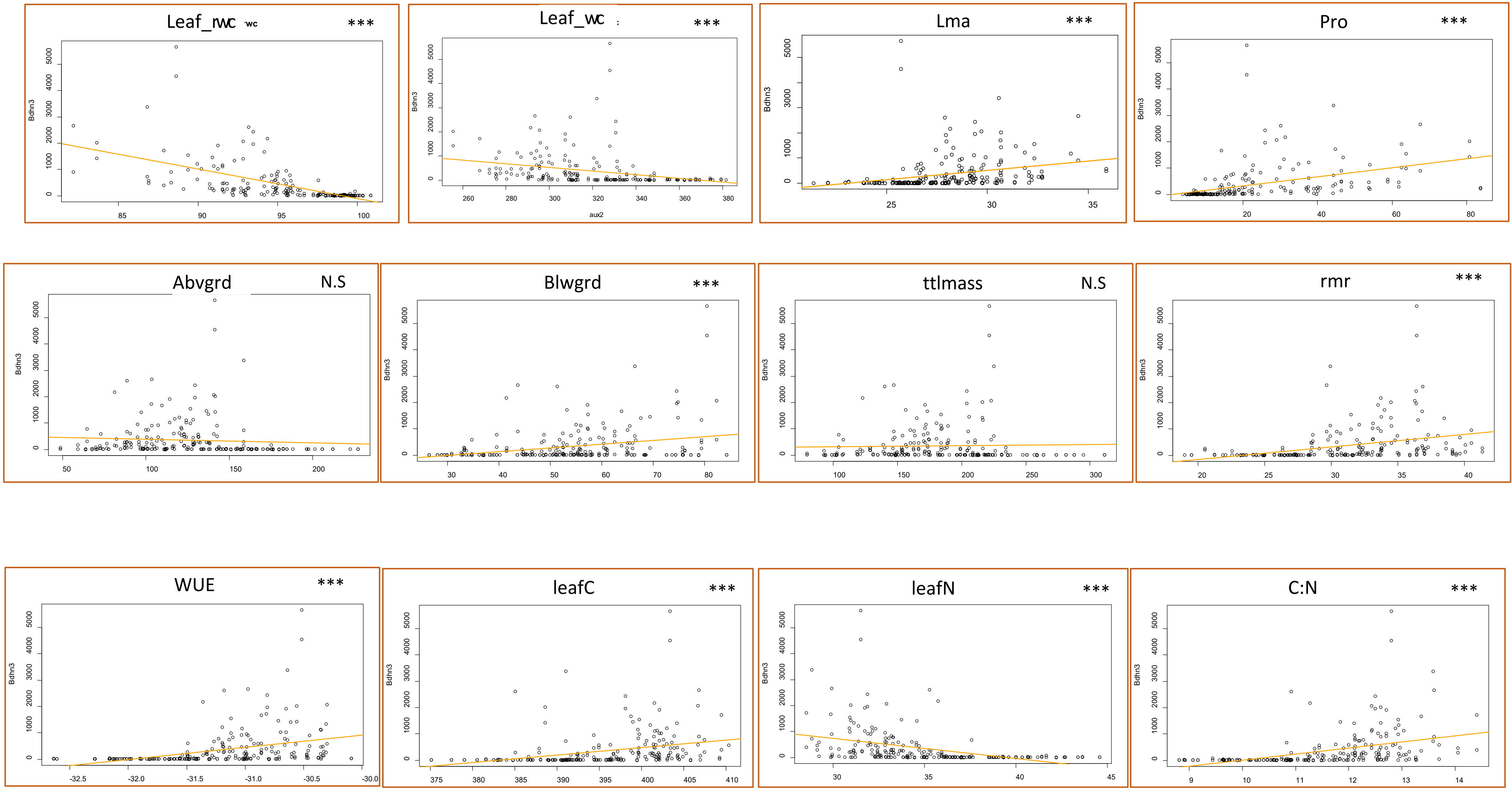

Figure S11

Bdhn7vs phenotypic traits

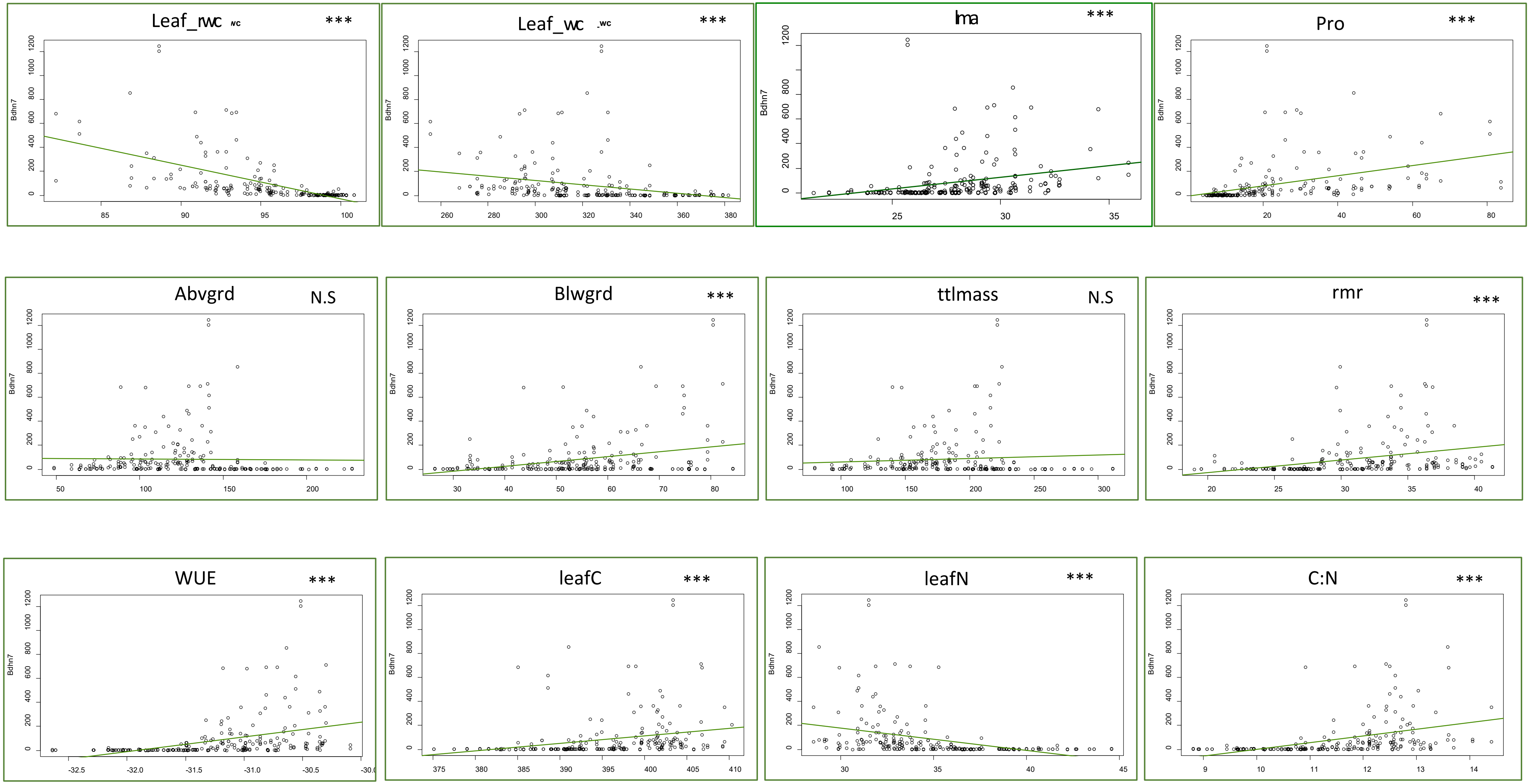

Figure S12

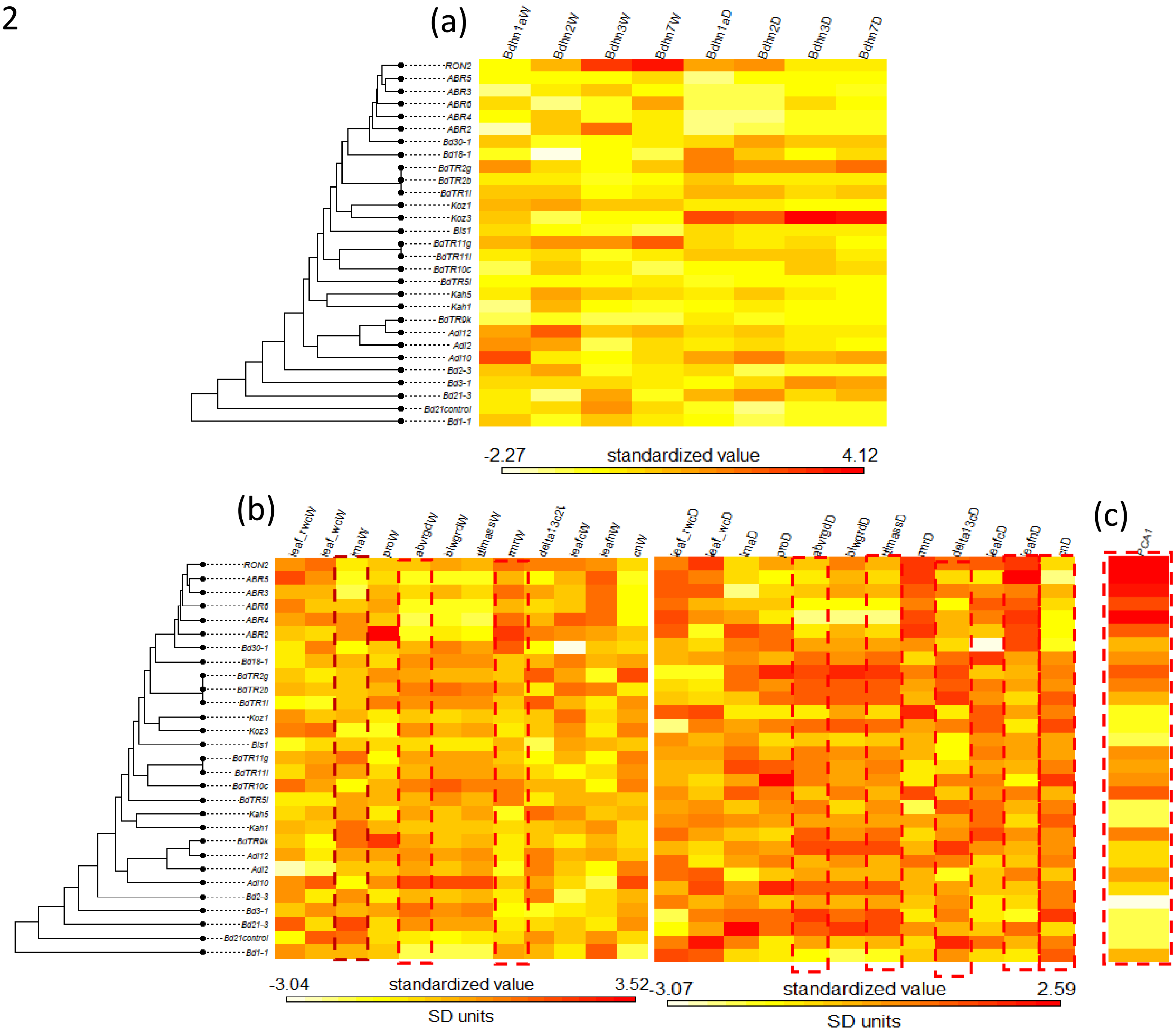

(a)
Bdhn1aW
Bdhn2W
Bdhn3W
Bdhn7W
Bdhn1aD
Bdhn2D
Bdhn3D
Bdhn7D
RON2
ABR5
ABR3
ABR6
ABR4
ABR2
Bd30-1
Bd18-1
BdTR2g
BdTR2b
BdTR1i
Koz1
Koz3
Bis1
BdTR11g
BdTR11I
BdTR10c
BdTR5i
Kah5
Kah1
BdTR9k
Adi12
Adi2
Adi10
Bd2-3
Bd3-1
Bd21-3
Bd21control
Bd1-1
-2.27
standardized value
4.12
(b)
-3.04
standardized value
3.52
SD units
-3.07
standardized value
2.59
SD units
(c)
PCA1

Supplementary Materials

Supplementary Materials S1. Branch-site aBSREL (adaptive Branch-Site Random Effects Likelihood) and BUSTED (Branch-Site Unrestricted Statistical Test for Episodic Diversification) and site MEME (Mixed Effects Model of Evolution) tests models for potential positive selections in each of the 10 Bdhn genes across the four studied species of *Brachypodium* (five genomes/subgenomes) conducted with Datamonkey2 (https://www.datamonkey.org/). Results from all selection tests are shown for **(a)** aBSREL and **(b)** BUSTED models, which searched for positive selection at branch-site across the entire phylogeny and at internal nodes or leaf nodes, and for **(c)** MEME (Mixed Effects Model of Evolution) model, which tested for potential diversifying selection at individual sites under a proportion of branches. Significant p-values (positive selection) are highlighted in bold.

(a) aBSREL

	aBSREL						
		All branches		internal branches		leaf branches	
**Bdhn**	**Name**	**Test p-value**	**ω distribution over sites**	**Test p-value**	**ω distribution over sites**	**Test p-value**	**ω distribution over sites**
Bdhn1_2	BDHNBDIS1	1	10000000000	1	10000000000	1	10000000000
Bdhn1_2	BDHNBDIS2	1	10000000000	1	10000000000	1	10000000000
Bdhn1_2	BDHNBHYBD1	1	10000000000	1	10000000000	1	10000000000
Bdhn1_2	BDHNBHYBD2	1	0.00	1	0.00	1	0.00
Bdhn1_2	BDHNBHYBS1	1	1.00	1	1.00	1	1.00
Bdhn1_2	BDHNBHYBS2	1	10000000000	1	10000000000	1	10000000000
Bdhn1_2	BDHNBSTA1	1	0.00	1	0.00	1	0.00
Bdhn1_2	BDHNBSTA2	1	10000000000	1	10000000000	1	10000000000
Bdhn1_2	BDHNBSYL1A	1	0.123	1	0.123	1	0.123

Bdhn1_2	BDHNBSYL1B	1	0.264	1	0.264	1	0.264
Bdhn1_2	BDHNBSYL2	1	0.00	1	0.00	1	0.00
Bdhn1_2	BDHNBSYL2		3.65		3.65		3.65
Bdhn1_2	Node10	1	0.151	1	0.151	1	0.151
Bdhn1_2	Node14	1	0.253	1	0.253	1	0.253
Bdhn1_2	Node17	1	0.00	1	0.00	1	0.00
Bdhn1_2	Node2	1	0.332	1	0.332	1	0.332
Bdhn1_2	Node5	1	0.152	1	0.152	1	0.152
Bdhn1_2	Node6	1	0.778	1	0.778	1	0.778
	Node7	1	0.00	0,8289	0.00	1	0.00
Bdhn1_2	Node7		7.74		7.74		7.74
Bdhn3	BDHNBDIS3	1	0.00	1	0.00	1	0.00
Bdhn3	BDHNBHYBD3	1	0.00	1	0.00	1	0.00
Bdhn3	BDHNBHYBS3	1	10000000000	1	10000000000	1	10000000000
Bdhn3	BDHNBSTA3	1	10000000000	1	10000000000	1	10000000000
Bdhn3	BDHNBSYL3	1	0.00	1	0.00	1	0.00
Bdhn3	Node1	1	0.121	1	0.121	1	0.121
	Node5	1	0.126	1	0.126	1	0.126
Bdhn4_5	BDHNBDIS4	1	0.592	1	0.592	1	0.592
Bdhn4_5	BDHNBDIS5	1	0.00	1	0.00	1	0.00
Bdhn4_5	BDHNBHYBS5	1	1.00	1	1.00	1	1.00
Bdhn4_5	BDHNBSTA5	1	0.0778	1	0.0778	1	0.0778
Bdhn4_5	BDHNBSYL5	1	0.261	1	0.261	1	0.261
Bdhn4_5	Node3	1	0.0646	1	0.0646	1	0.0646
Bdhn4_5	Node5	1	0.00	0,3577	0.00	1	0.00
Bdhn4_5	Node5		5.37		5.37		5.37
Bdhn6	BDHNBDIS6	1	0.593	1	0.593	1	0.593

Bdhn6	BDHNBHYBD6	1	0.239	1	0.239	1	0.239
Bdhn6	BDHNBHYBS6	0,278	0.00	1	0.00	0,2316	0.00
Bdhn6	BDHNBHYBS6		225		225		225
Bdhn6	BDHNBSTA6	1	0.00	1	0.00	1	0.00
Bdhn6	BDHNBSYL6	1	0.120	1	0.120	1	0.120
Bdhn6	Node1	0,5211	0.00	0,1042	0.00	1	0.00
Bdhn6	Node1		3.58		3.58		3.58
**Bdhn6**	Node5	0,1398	0.140	**0,0399**	**0.140**	1	0.140
Bdhn6	Node5		53.7		53.7		53.7
Bdhn7	BDHNBDIS7	1	0.279	1	0.0565	1	0.279
Bdhn7	BDHNBHYBD7	1	1.00	1	0.279	1	1.00
Bdhn7	BDHNBSTA7	1	0.0707	1	1.00	1	0.0707
Bdhn7	BDHNBSYL7	1	0.0180	1	0.0707	1	0.0180
Bdhn7	Node1	1	0.0565	1	0.0180	1	0.0565
Bdhn8	BDHNBDIS8	1	1.00	1	0.288	1	1.00
Bdhn8	BDHNBHYBD8	1	0.00	1	0.289	1	0.00
Bdhn8	BDHNBHYBS8	1	10000000000	1	1.00	1	10000000000
Bdhn8	BDHNBSTA8	1	1.00	1	0.00	1	1.00
Bdhn8	BDHNBSYL8	1	0.166	1	10000000000	1	0.166
Bdhn8	Node1	1	0.288	1	1.00	1	0.288
Bdhn8	Node2	1	0.289	1	0.166	1	0.289
Bdhn9	BDHNBDIS9	1	10000000000	1	0.417	1	10000000000
Bdhn9	BDHNBHYBD9	1	0.417	1	0.264	1	0.417
Bdhn9	BDHNBSTA9	0,1976	0.264	1	0.473	0,1581	0.264
Bdhn9	BDHNBSTA9		14.3	1	10000000000		14.3
Bdhn9	BDHNBSYL9	1	0.319		14.3	1	0.319
Bdhn9	Node1	1	0.473	1	0.319	1	0.473

Bdhn10	BDHBSYL10	1	0.00	1	0.00	1	1.00
Bdhn10	BDHNBDIS10	1	1.00	1	1.00	1	0.00
Bdhn10	BDHNBHYBD10	1	1.00	1	1.00	1	0.00
Bdhn10	BDHNBSTA10	1	0.00	1	0.00		100000
**Bdhn10**	Node1	0,1123	0.00	**0,0225**	**0.00**	1	0.00
Bdhn10	Node1		100000		100000	1	1.00

(b) BUSTED

ALL BRANCHES

BDHN1_2					all branches			
Model	log L	#. params	AICc	CV(SRV)	Branch set	ω1	ω2	ω3
Unconstrained model	-1723,4	43	3534,1	0,308	Test	0.00 (13.23%)	0.00 (78.46%)	2.90 (8.30%)
Constrained model	-1724,4	42	3534,1	0,349	Test	0.00 (25.62%)	0.00 (54.32%)	1.00 (20.06%)
found no evidence								

INTERNAL BRANCHES

Bdhn1_2					internal branches			
Model	log L	#. params	AICc	CV(SRV)	Branch set	ω1	ω2	ω3
Unconstrained model	-1723,3	48	3544,3	0,303	Test	0.00 (79.87%)	0.00 (13.27%)	3.57 (6.86%)
					Background	0.00 (83.59%)	0.53 (11.03%)	3.36 (5.39%)
Constrained model	-1724,1	47	3543,7	0,332	Test	0.00 (71.10%)	0.00 (9.40%)	1.00 (19.50%)
					Background	0.00 (83.54%)	0.52 (10.93%)	3.31 (5.54%)
found no evidence								

LEAF BRANCHES

Bdhn1_2					leaf branches			
Model	log L	#. params	AICc	CV(SRV)	Branch set	ω1	ω2	ω3
Unconstrained model	-1723,3	48	3544,3	0,302	Test	0.00 (59.60%)	0.00 (30.84%)	2.51 (9.55%)
					Background	0.00 (12.11%)	0.00 (81.16%)	3.65 (6.73%)
Constrained model	-1723,7	47	3542,9	0,319	Test	0.00 (67.21%)	0.00 (12.04%)	1.00 (20.75%)
					Background	0.00 (12.25%)	0.00 (80.94%)	3.60 (6.80%)
found no evidence								

BDHN3					all branches			
Model	log L	#. params	AICc	CV(SRV)	Branch set	ω1	ω2	ω3
Unconstrained model	-794,4	31	1653,2	0	Test	0.11 (100.00%)	0.14 (0.00%)	1.00 (0.00%)

Bdhn3					internal branches			
Model	log L	#. params	AICc	CV(SRV)	Branch set	ω1	ω2	ω3
Unconstrained model	-795,4	36	1666,1	2,902	Test	0.00 (55.36%)	0.00 (37.97%)	1.81 (6.68%)
					Background	0.19 (46.19%)	0.23 (53.81%)	0.67 (0.00%)

Bdhn3					leaf branches			
Model	log L	#. params	AICc	CV(SRV)	Branch set	ω1	ω2	ω3
Unconstrained model	-794,3	36	1663,8	0	Test	0.00 (21.45%)	0.03 (71.90%)	1.00 (6.65%)
					Background	0.12 (100.00%)	0.15 (0.00%)	0.17 (0.00%)

Model	log L	#. params	AICc	CV(SRV)	Branch set	ω1	ω2	ω3
found no evidence								

Model	log L	#. params	AICc	CV(SRV)	Branch set	ω1	ω2	ω3
Constrained model	-795,5	35	1664,2	2,913	Test	0.00 (79.03%)	0.00 (11.86%)	1.00 (9.11%)
					Background	0.20 (62.01%)	0.21 (15.83%)	0.23 (22.16%)
found no evidence								

Model	log L	#. params	AICc	CV(SRV)	Branch set	ω1	ω2	ω3
found no evidence								

Bdhn4_5 all branches

Model	log L	#. params	AICc	CV(SRV)	Branch set	ω1	ω2	ω3
Unconstrained model	-825,6	31	1715,8	0	Test	0.00 (55.23%)	0.00 (35.58%)	1.69 (9.19%)
Constrained model	-825,8	30	1714	0,218	Test	0.00 (60.08%)	0.00 (25.88%)	1.00 (14.04%)
found no evidence								

Bdhn4_5 internal branches

Model	log L	#. params	AICc	CV(SRV)	Branch set	ω1	ω2	ω3
Unconstrained model	-823,9	36	1723,1	0	Test	0.00 (9.54%)	0.00 (87.40%)	5.40 (3.07%)
					Background	0.18 (100.00%)	0.59 (0.00%)	2.79 (0.00%)
Constrained model	-825	35	1723,2	0,45	Test	0.00 (20.44%)	0.00 (71.09%)	1.00 (8.47%)
					Background	0.14 (96.77%)	1.00 (0.91%)	1.82 (2.32%)
found no evidence								

Bdhn4_5 leaf branches

Model	log L	#. params	AICc	CV(SRV)	Branch set	ω1	ω2	ω3
Unconstrained model	-823,9	36	1723,1	0	Test	0.18 (100.00%)	0.24 (0.00%)	1.00 (0.00%)
					Background	0.00 (4.78%)	0.00 (92.18%)	5.43 (3.04%)
found no evidence								

Bdhn6 all branches

Model	log L	#. params	AICc	CV(SRV)	Branch set	ω1	ω2	ω3
Unconstrained model	-2499	31	5060,8	1,097	Test	0.03 (68.37%)	0.20 (27.91%)	4.43 (3.72%)
Constrained model	-2499,7	30	5060,3	1,006	Test	0.00 (17.72%)	0.00 (62.95%)	1.00 (19.33%)
found no evidence								

Bdhn6 internal branches

Model	log L	#. params	AICc	CV(SRV)	Branch set	ω1	ω2	ω3
Unconstrained model	-2495,7	36	5064,5	8,319	Test	0.00 (1.56%)	0.00 (84.83%)	2.10 (13.60%)
					Background	0.02 (81.96%)	0.02 (12.97%)	2.17 (5.07%)
Constrained model	-2496,7	35	5064,4	8,424	Test	0.00 (78.00%)	0.00 (0.00%)	1.00 (22.00%)
					Background	0.02 (83.89%)	0.02 (11.73%)	2.81 (4.38%)
found no evidence								

Bdhn6 leaf branches

Model	log L	#. params	AICc	CV(SRV)	Branch set	ω1	ω2	ω3
Unconstrained model	-2494,8	36	5062,7	7,66	Test	0.03 (6.36%)	0.03 (88.61%)	2.05 (5.03%)
					Background	0.00 (80.21%)	1.00 (18.39%)	23.84 (1.40%)
Constrained model	-2495	35	5061	9,451	Test	0.02 (8.26%)	0.02 (81.41%)	1.00 (10.33%)
					Background	0.00 (80.32%)	1.00 (18.26%)	23.58 (1.42%)
found no evidence								

Bdhn7 all branches

Bdhn7 internal branches

Bdhn7 leaf branches

Model	log L	#. params	AICc	CV(SRV)	Branch set	ω1	ω2	ω3
Unconstrained model	-893,8	29	1848,1	1,003	Test	0.04 (89.57%)	0.05 (10.43%)	1.51 (0.00%)

Phylogenetic Alignment cannot be rendered for this job

found no evidence

Model	log L	#. params	AICc	CV(SRV)	Branch set	ω1	ω2	ω3
Unconstrained model	-892	34	1855,4	0,994	Test	0.04 (100.00%)	0.12 (0.00%)	1.39 (0.00%)
					Background	0.00 (97.33%)	0.02 (0.00%)	20.76 (2.67%)

Phylogenetic Alignment cannot be rendered for this job.

found no evidence

Model	log L	#. params	AICc	CV(SRV)	Branch set	ω1	ω2	ω3
Unconstrained model	-892	34	1855,4	0,994	Test	0.00 (3.75%)	0.00 (93.59%)	20.77 (2.66%)
					Background	0.04 (100.00%)	0.19 (0.00%)	14.28 (0.00%)
Constrained model	-893,6	33	1856,5	1,007	Test	0.00 (5.89%)	0.00 (88.30%)	1.00 (5.81%)
					Background	0.04 (100.00%)	0.18 (0.00%)	14.64 (0.00%)

found no evidence

Bdhn8 all branches

Model	log L	#. params	AICc	CV(SRV)	Branch set	ω1	ω2	ω3
Unconstrained model	-919,8	31	1904	0,436	Test	0.01 (45.60%)	0.02 (32.73%)	1.09 (21.67%)
Constrained model	-919,8	30	1901,8	0,441	Test	0.00 (39.91%)	0.00 (35.61%)	1.00 (24.48%)

found no evidence

Bdhn8 internal branches

Model	log L	#. params	AICc	CV(SRV)	Branch set	ω1	ω2	ω3
Unconstrained model	-919,4	36	1914	0,453	Test	0.00 (75.71%)	1.00 (3.37%)	1.38 (20.92%)
					Background	0.06 (77.14%)	0.29 (18.09%)	1.79 (4.77%)
Constrained model	-919,4	35	1911,9	0,478	Test	0.00 (69.94%)	1.00 (26.07%)	1.00 (3.99%)
					Background	0.06 (78.01%)	0.32 (17.31%)	1.75 (4.68%)

found no evidence

Bdhn8 leaf branches

Model	log L	#. params	AICc	CV(SRV)	Branch set	ω1	ω2	ω3
Unconstrained model	-919,4	36	1914	0,45	Test	0.09 (93.50%)	1.00 (0.00%)	1.57 (6.50%)
					Background	0.00 (76.27%)	0.00 (0.00%)	1.35 (23.72%)
Constrained model	-919,4	35	1911,8	0,455	Test	0.06 (86.67%)	0.92 (0.00%)	1.00 (13.33%)
					Background	0.00 (75.76%)	0.00 (0.04%)	1.32 (24.20%)

found no evidence

Bdhn9 all branches

Model	log L	#. params	AICc	CV(SRV)	Branch set	ω1	ω2	ω3
Unconstrained model	-1187,7	29	2435,3	1,256	Test	0.00 (40.10%)	0.00 (15.17%)	1.00 (44.73%)

Bdhn9 internal branches

Model	log L	#. params	AICc	CV(SRV)	Branch set	ω1	ω2	ω3
Unconstrained model	-1187,6	34	2445,8	1,105	Test	0.40 (0.00%)	0.41 (100.00%)	1.88 (0.00%)

Bdhn9 leaf branches

Model	log L	#. params	AICc	CV(SRV)	Branch set	ω1	ω2	ω3
Unconstrained model	-1187,6	34	2445,9	1,274	Test	0.00 (48.79%)	0.70 (33.75%)	1.16 (17.46%)
					Background	0.41 (100.00%)	0.47 (0.00%)	0.83 (0.00%)
Constrained model	-1187,6	33	2443,8	1,321	Test	0.00 (45.67%)	0.72 (41.39%)	1.00 (12.95%)

Branch set	ω1	ω2	ω3
Background	0.41 (99.39%)	0.42 (0.60%)	0.42 (0.01%)

found no evidence

found no evidence

found no evidence

Bdhn10 all_branches

Model	log L	#. params	AICc	CV(SRV)	Branch set	ω1	ω2	ω3
Unconstrained model	-337,9	29	738,1	1,968	Test	0.00 (9.95%)	0.00 (89.52%)	9999999171.60 (0.53%)
Constrained model	-338,8	28	737,6	1,463	Test	0.00 (0.00%)	0.00 (100.00%)	1.00 (0.00%)
found no evidence								

Bdhn10 internal_branches

Model	log L	#. params	AICc	CV(SRV)	Branch set	ω1	ω2	ω3
Unconstrained model	-337,3	34	748,6	4,159	Test	0.00 (0.00%)	0.07 (100.00%)	1.15 (0.00%)
					Background	0.00 (100.00%)	0.49 (0.00%)	1.14 (0.00%)
found no evidence								

Bdhn10 leaf_branches

Model	log L	#. params	AICc	CV(SRV)	Branch set	ω1	ω2	ω3
Unconstrained model	-337,1	34	748,2	2,353	Test	0.00 (100.00%)	0.03 (0.00%)	1.16 (0.00%)
					Background	0.00 (16.97%)	0.00 (81.95%)	10000.00 (1.09%)
found no evidence								

(c) MEME

MEME results															
BDHN1_2		BDHN3		BDHN4_5		BDHN6		BDHN7		BDHN8		BDHN9		BDHN10	
Site	p-value	Site	p-value	Site	p-value	Site	p-value	Site	p-value	Site	p-value	Site	p-value	Site	p-value
**139**	**0,04**	1	1	1	1	**266**	**0,04**	1	1	1	1	1	1	1	1
**163**	**0,02**	2	1	2	1	**412**	**0,05**	2	0,67	2	1	2	1	2	1
1	1	3	1	3	1	1	1	3	1	3	0,67	3	1	3	1
2	1	4	1	4	1	2	1	4	1	4	1	4	1	4	1
3	0,67	5	0,67	5	1	3	0,67	5	1	5	1	5	1	5	1
4	1	6	1	6	1	4	1	6	0,67	6	0,67	6	1	6	1
5	0,67	7	1	7	1	5	1	7	1	7	1	7	1	7	1
6	0,33	8	0,67	8	0,67	6	0,67	8	1	8	0,55	8	0,67	8	1
7	0,67	9	1	9	1	7	1	9	1	9	1	9	1	9	1
8	1	10	1	10	1	8	1	10	1	10	0,5	10	1	10	1
9	0,45	11	1	11	1	9	1	11	1	11	1	11	1	11	1
10	1	12	1	12	0,67	10	0,67	12	1	12	1	12	1	12	1
11	1	13	1	13	1	11	1	13	1	13	1	13	1	13	1
12	1	14	0,67	14	1	12	1	14	0,55	14	0,55	14	1	14	1
13	0,37	15	1	15	1	13	0,34	15	1	15	1	15	1	15	1
14	1	16	1	16	1	14	1	16	1	16	1	16	1	16	0,67
15	1	17	1	17	0,67	15	0,67	17	1	17	1	17	1	17	1
16	0,67	18	1	18	1	16	1	18	1	18	1	18	0,67	18	1
17	1	19	1	19	1	17	1	19	1	19	0,67	19	1	19	1
18	0,67	20	1	20	1	18	1	20	1	20	1	20	1	20	1
19	1	21	1	21	1	19	1	21	0,67	21	0,67	21	1	21	1

20	1	22	1	22	1	20	0,52	22	1	22	0,67	22	1	22	1
21	1	23	1	23	1	21	1	23	0,67	23	0,67	23	1	23	1
22	1	24	1	24	1	22	1	24	0,67	24	0,67	24	1	24	1
23	0,67	25	1	25	0,32	23	0,67	25	0,67	25	0,67	25	1	25	1
24	0,67	26	0,48	26	1	24	0,67	26	1	26	1	26	1	26	1
25	0,67	27	0,67	27	1	25	0,67	27	1	27	1	27	1	27	1
26	1	28	1	28	1	26	0,36	28	1	28	0,67	28	1	28	0,67
27	1	29	1	29	1	27	0,35	29	1	29	0,31	29	1	29	1
28	0,67	30	1	30	1	28	1	30	1	30	1	30	1	30	1
29	1	31	1	31	1	29	1	31	1	31	1	31	1	31	1
30	1	32	0,67	32	1	30	0,67	32	1	32	1	32	1	32	1
31	1	33	1	33	1	31	1	33	0,67	33	1	33	1	33	1
32	1	34	1	34	1	32	1	34	0,67	34	0,67	34	1	34	1
33	0,67	35	0,67	35	1	33	0,34	35	1	35	1	35	1	35	1
34	1	36	1	36	0,67	34	1	36	1	36	1	36	0,4	36	1
35	0,52	37	1	37	1	35	0,37	37	1	37	0,55	37	1	37	1
36	1	38	1	38	1	36	1	38	0,37	38	1	38	0,67	38	1
37	1	39	1	39	0,67	37	0,67	39	0,67	39	0,38	39	0,67	39	1
38	1	40	1	40	1	38	1	40	1	40	0,67	40	1	40	0,67
39	1	41	1	41	1	39	0,67	41	1	41	1	41	0,42	41	0,67
40	1	42	1	42	1	40	0,67	42	1	42	1	42	1	42	1
41	1	43	1	43	1	41	1	43	1	43	1	43	1	43	1
42	1	44	1	44	1	42	1	44	1	44	1	44	1	44	1
43	1	45	1	45	1	43	1	45	1	45	0,67	45	1	45	1
44	1	46	1	46	1	44	0,67	46	1	46	1	46	0,67	46	1
45	1	47	1	47	1	45	1	47	1	47	1	47	1	47	1
46	1	48	1	48	1	46	0,08	48	1	48	1	48	0,62	48	1
47	1	49	1	49	1	47	0,56	49	1	49	1	49	1	49	1

48	0,67	50	0,45	50	1	48	1	50	0,67	50	1	50	1	50	1
49	0,07	51	1	51	0,57	49	1	51	1	51	0,53	51	1	51	0,67
50	1	52	1	52	1	50	1	52	1	52	0,67	52	0,67	52	1
51	1	53	1	53	0,67	51	0,67	53	1	53	1	53	0,22	53	1
52	1	54	1	54	1	52	0,34	54	1	54	1	54	1	54	1
53	1	55	0,67	55	0,67	53	0,67	55	1	55	1	55	0,39	55	1
54	1	56	1	56	1	54	0,25	56	1	56	1	56	0,67	56	1
55	1	57	1	57	1	55	0,67	57	1	57	1	57	0,67	57	1
56	1	58	1	58	1	56	0,52	58	1	58	0,67	58	1	58	1
57	1	59	1	59	1	57	1	59	1	59	0,28	59	1	59	1
58	1	60	1	60	1	58	1	60	1	60	0,67	60	1	60	1
59	1	61	1	61	1	59	1	61	1	61	1	61	1	61	1
60	0,5	62	1	62	1	60	1	62	1	62	0,44	62	0,67	62	1
61	1	63	1	63	1	61	1	63	1	63	0,54	63	1	63	0,67
62	1	64	1	64	0,67	62	0,67	64	1	64	1	64	0,56	64	1
63	0,45	65	0,67	65	1	63	0,39	65	1	65	1	65	0,12	65	1
64	0,67	66	1	66	1	64	1	66	1	66	1	66	0,45	66	1
65	0,67	67	1	67	1	65	1	67	1	67	1	67	0,52	67	1
66	1	68	1	68	1	66	0,24	68	1	68	1	68	1	68	1
67	0,45	69	1	69	0,52	67	0,67	69	1	69	0,47	69	1	69	1
68	0,49	70	1	70	1	68	1	70	1	70	1	70	0,26	70	1
69	1	71	1	71	1	69	1	71	1	71	0,67	71	1	71	0,67
70	0,67	72	1	72	1	70	1	72	1	72	1	72	1	72	1
71	1	73	1	73	0,67	71	1	73	1	73	1	73	1	73	1
72	0,52	74	1	74	0,67	72	1	74	1	74	1	74	0,67	74	1
73	1	75	0,37	75	1	73	1	75	1	75	1	75	0,67	75	1
74	1	76	1	76	1	74	0,67	76	1	76	1	76	1	76	1
75	1	77	1	77	0,67	75	0,42	77	0,67	77	1	77	1	77	1

76	1	78	1	78	1	76	1	78	1	78	1	78	1	78	1
77	1	79	1	79	1	77	0,67	79	0,67	79	1	79	1	79	1
78	0,67	80	1	80	0,67	78	1	80	1	80	1	80	1	80	1
79	1	81	0,67	81	1	79	0,46	81	1	81	1	81	1	81	1
80	1	82	1	82	1	80	1	82	1	82	1	82	0,36	82	1
81	1	83	1	83	1	81	0,29	83	0,67	83	1	83	1	83	1
82	1	84	1	84	1	82	0,23	84	1	84	1	84	1	84	1
83	0,67	85	1	85	0,67	83	0,21	85	1	85	1	85	0,36	85	1
84	1	86	1	86	1	84	0,18	86	1	86	1	86	1	86	1
85	1	87	1	87	1	85	0,67	87	1	87	1	87	1	87	1
86	0,67	88	1	88	1	86	0,38	88	1	88	1	88	1	88	1
87	1	89	0,67	89	0,67	87	1	89	1	89	1	89	1	89	1
88	1	90	0,18	90	1	88	1	90	1	90	1	90	1	90	1
89	1	91	1	91	0,55	89	0,56	91	1	91	1	91	0,67	91	1
90	0,67	92	1	92	0,67	90	1	92	1	92	1	92	0,42	92	1
91	1	93	0,47	93	1	91	0,22	93	1	93	1	93	0,67	93	1
92	1	94	0,67	94	1	92	0,67	94	1	94	1	94	1	94	1
93	1	95	1	95	1	93	1	95	1	95	1	95	0,67	95	1
94	1	96	1	96	1	94	1	96	1	96	1	96	1	96	1
95	1	97	1	97	1	95	0,39	97	1	97	1	97	1	97	1
96	1	98	1	98	1	96	0,67	98	1	98	1	98	1	98	1
97	1	99	1	99	1	97	1	99	0,67	99	1	99	0,37	99	1
98	0,16	100	1	100	0,67	98	0,67	100	0,67	100	1	100	1	100	1
99	0,67	101	1	101	1	99	1	101	1	101	0,67	101	0,67	101	1
100	1	102	1	102	1	100	0,67	102	1	102	1	102	1	102	1
101	0,67	103	1	103	1	101	1	103	1	103	0,67	103	1	103	1
102	0,67	104	1	104	1	102	1	104	1	104	1	104	1	104	1
103	1	105	1	105	0,67	103	1	105	1	105	1	105	1	105	1

104	0,67	106	1	106	1	104	0,67	106	1	106	0,67	106	0,67	106	1
105	1	107	1	107	0,58	105	1	107	1	107	0,67	107	1	107	1
106	1	108	1	108	1	106	1	108	1	108	1	108	1	108	1
107	1	109	0,38	109	0,58	107	0,67	109	1	109	0,4	109	1		
108	1	110	0,67	110	0,45	108	1	110	1	110	1	110	1		
109	1	111	1	111	1	109	1	111	0,67	111	1	111	1		
110	0,67	112	1	112	0,67	110	1	112	0,67	112	1	112	1		
111	0,67	113	0,67	113	1	111	0,67	113	0,67	113	1	113	1		
112	0,67	114	0,67	114	0,4	112	0,67	114	1	114	0,67	114	1		
113	0,67	115	1	115	0,67	113	0,67	115	0,67	115	0,67	115	1		
114	1	116	1	116	1	114	1	116	1	116	0,67	116	1		
115	1	117	1	117	1	115	1	117	1	117	0,67	117	1		
116	1	118	0,45	118	1	116	1	118	0,67	118	0,67	118	0,61		
117	1	119	1	119	1	117	1	119	1	119	1	119	1		
118	1	120	1	120	1	118	1	120	1	120	0,36	120	1		
119	1	121	1	121	1	119	1	121	0,67	121	1	121	0,45		
120	1	122	1	122	1	120	1	122	0,67	122	1	122	1		
121	0,67	123	1	123	1	121	0,34	123	0,67	123	1	123	1		
122	0,67	124	1	124	1	122	0,67	124	1	124	1	124	0,48		
123	0,67	125	1	125	1	123	0,67	125	1	125	0,67	125	1		
124	1	126	1	126	1	124	1	126	1	126	0,67	126	1		
125	1	127	1	127	0,25	125	0,67	127	0,67	127	0,67	127	1		
126	1	128	1	128	0,67	126	0,67	128	0,67	128	1	128	0,49		
127	0,67	129	1	129	0,4	127	1	129	0,08	129	1	129	1		
128	1	130	1	130	1	128	0,67	130	1	130	0,67	130	1		
129	1	131	1	131	1	129	0,67	131	1	131	1	131	1		
130	1	132	1	132	1	130	0,49	132	1	132	1	132	0,59		
131	0,54	133	0,67	133	1	131	0,38	133	1	133	1	133	0,2		

132	1	134	1	134	1	132	1	134	1	134	1	134	1
133	0,67	135	0,27	135	0,67	133	0,67	135	1	135	1	135	1
134	0,67	136	1	136	1	134	1	136	1	136	1	136	1
135	0,22	137	1	137	1	135	1	137	0,67	137	1	137	1
136	1	138	1	138	0,67	136	1	138	0,67	138	0,38	138	1
137	0,29	139	0,47	139	0,32	137	1	139	0,67	139	1	139	1
138	1	140	0,67	140	0,3	138	1	140	1	140	1	140	1
140	0,67	141	1	141	0,67	139	1	141	0,67	141	1	141	1
141	0,67	142	1	142	0,67	140	1	142	1	142	1	142	1
142	0,67	143	1	143	1	141	1	143	0,67	143	1	143	1
143	0,67	144	1	144	1	142	0,67	144	0,67	144	0,36	144	1
144	0,44	145	1	145	1	143	1	145	0,67	145	1	145	1
145	0,18	146	1	146	1	144	1	146	1	146	1	146	0,67
146	0,67	147	0,67	147	1	145	1	147	1	147	1	147	1
147	0,67	148	1	148	1	146	1	148	1	148	0,37	148	1
148	1	149	1	149	1	147	0,67	149	1	149	1	149	1
149	1	150	1	150	1	148	1	150	1	150	0,35	150	1
150	0,67	151	1	151	0,67	149	0,67	151	1	151	1	151	1
151	0,3	152	1	152	0,67	150	1	152	1	152	0,67	152	1
152	1	153	1	153	1	151	1	153	1	153	1	153	0,39
153	0,67	154	0,67	154	1	152	1	154	1	154	0,56	154	1
154	0,67	155	1	155	1	153	0,67	155	1	155	0,38	155	1
155	0,67	156	1	156	1	154	1	156	1	156	1	156	1
156	1	157	1	157	1	155	0,37	157	1	157	1	157	1
157	0,67	158	1	158	1	156	0,67	158	0,67	158	1	158	1
158	1	159	1	159	1	157	0,67	159	1	159	1	159	1
159	1	160	1	160	1	158	0,67	160	0,67	160	1	160	0,31
160	1	161	1	161	1	159	1	161	0,67	161	1	161	0,67

161	0,67	162	1	162	1	160	0,67	162	1	162	1	162	1
162	0,67	163	1	163	1	161	1	163	1	163	1	163	0,67
164	1	164	1	164	1	162	0,33	164	0,67	164	1	164	0,67
165	0,41	165	1			163	1	165	1	165	1	165	1
166	1	166	1			164	1	166	0,67	166	1	166	1
167	1	167	1			165	1	167	0,67	167	1	167	1
168	1	168	1			166	1	168	1	168	1	168	1
169	0,67	169	0,56			167	0,67	169	1	169	1	169	1
170	1	170	1			168	1	170	1	170	0,67	170	1
171	0,08					169	1	171	1	171	0,38	171	1
172	0,67					170	0,67	172	1	172	1	172	1
173	1					171	1	173	1	173	1	173	1
174	1					172	1	174	1			174	1
175	1					173	1	175	1			175	1
176	0,33					174	1	176	1			176	1
177	1					175	1	177	1			177	1
178	1					176	1	178	1			178	1
179	0,67					177	1	179	1			179	1
180	1					178	0,67	180	1			180	1
181	1					179	0,67	181	1			181	1
182	1					180	1	182	1			182	0,07
183	1					181	1	183	1			183	1
184	1					182	1	184	1			184	1
185	1					183	1					185	1
186	1					184	1					186	1
187	1					185	1					187	0,67
188	0,67					186	0,67					188	1

189	0,67
190	1
191	1
192	1
193	0,52
194	1
195	1
196	1
197	0,32
198	0,65
199	0,67
200	1
201	1
202	1
203	1
204	1
205	0,67
206	0,3
207	0,23
208	1
209	0,67
210	1
211	0,67
212	1
213	1
214	0,27
215	0,67
216	0,39

187	1
188	1
189	1
190	0,67
191	0,67
192	1
193	0,67
194	0,43
195	0,67
196	1
197	1
198	1
199	0,2
200	0,67
201	0,67
202	1
203	1
204	1
205	1
206	1
207	1
208	1
209	1
210	1
211	1
212	1
213	1
214	1

189	1
190	1
191	0,67
192	1
193	1
194	0,47
195	0,47
196	1
197	1
198	0,38
199	1
200	1
201	1
202	1
203	1
204	1
205	0,67
206	1
207	1
208	1
209	1
210	1
211	1
212	1
213	1
214	1
215	1
216	1

217	1
218	0,44
219	0,67
220	0,67
221	1
222	0,67
223	1
224	0,67
225	0,45
226	1
227	1
228	1
229	1
230	1
231	1
232	1
233	1
234	0,67
235	1
236	1
237	1
238	1
239	1
240	1
241	0,38
242	1
243	1
244	1

215	1
216	1
217	1
218	1
219	1
220	1
221	1
222	1
223	1
224	1
225	1
226	1
227	1
228	1
229	0,33
230	0,67
231	1
232	0,67
233	1
234	0,67
235	1
236	0,67
237	0,67
238	0,67
239	1
240	1
241	0,67
242	1

217	1
218	1
219	1
220	1
221	1
222	1
223	1
224	1
225	1
226	1
227	1
228	1
229	1
230	1
231	1
232	1
233	1

245	1
246	1
247	1
248	0,67
249	0,42
250	1
251	1
252	1
253	1
254	1
255	0,67
256	0,67
257	0,67
258	1
259	0,67
260	1
261	0,39
262	1
263	1

243	1
244	1
245	1
246	1
247	1
248	1
249	1
250	1
251	1
252	1
253	0,67
254	1
255	1
256	1
257	1
258	0,67
259	0,07
260	1
261	0,37
262	1
263	0,67
264	0,67
265	1
267	1
268	0,38
269	0,67
270	1
271	1

272	0,67
273	1
274	1
275	1
276	1
277	1
278	1
279	1
280	1
281	1
282	1
283	1
284	1
285	1
286	1
287	1
288	1
289	1
290	1
291	1
292	1
293	1
294	1
295	1
296	0,67
297	1
298	1
299	1

300	1
301	1
302	1
303	1
304	1
305	1
306	1
307	1
308	1
309	1
310	1
311	1
312	0,67
313	1
314	1
315	1
316	0,67
317	1
318	1
319	1
320	1
321	1
322	1
323	1
324	1
325	1
326	1
327	1

328	1
329	1
330	1
331	1
332	1
333	1
334	1
335	1
336	1
337	1
338	1
339	1
340	1
341	1
342	1
343	1
344	1
345	0,67
346	1
347	1
348	1
349	1
350	1
351	1
352	1
353	1
354	0,67
355	0,09

356	0,67
357	1
358	1
359	1
360	0,32
361	0,67
362	1
363	1
364	1
365	1
366	1
367	1
368	1
369	1
370	1
371	0,67
372	0,67
373	1
374	0,62
375	1
376	0,25
377	0,67
378	1
379	1
380	1
381	1
382	1
383	1

384	0,67
385	1
386	1
387	1
388	1
389	0,67
390	1
391	1
392	0,67
393	1
394	0,5
395	1
396	1
397	0,5
398	0,52
399	0,67
400	1
401	1
402	1
403	1
404	1
405	1
406	0,67
407	1
408	1
409	1
410	0,67
411	0,67

413	1
414	1
415	1
416	0,67
417	1
418	1
419	1
420	1
421	1
422	1
423	1
424	1
425	1
426	0,67
427	1
428	0,67
429	1
430	1
431	0,67
432	1
433	1
434	0,67
435	0,67
436	0,67
437	1
438	1
439	1
440	0,67

	441	1	
	442	0,67	
	443	0,33	
	444	1	
	445	1	
	446	1	
	447	1	
	448	1	
	449	0,67	
	450	1	
	451	1	
	452	1	
	453	1	
	454	0,67	
	455	1	
	456	1	
	457	1	
	458	1	
	459	1	
	460	1	
	461	1	
	462	1	
	463	1	
	464	1	
	465	1	
	466	1	
	467	0,5	

Supplementary Materials S2. BLAST results for *Bdhn*4 and *Bdhn*5 after manual curation of the original sequences.

Variety	BDHN	Location	% identity	Align len	Strands	Target from	Target to	Bitscore	# identical
ABR2	Bdhn4	pseudomolecule_3	96	494	+/+	21107895	21108383	791,162	473
ABR2	Bdhn5	pseudomolecule_3	95	540	+/+	21106163	21106698	843.46	513
ABR3	Bdhn4	pseudomolecule_4	96	423	+/-	20641618	20641198	677.55	405
ABR3	Bdhn5	pseudomolecule_4	96	423	+/-	20639924	20639505	677.55	405
ABR4	Bdhn4	pseudomolecule_3	100	435	+/-	19329768	19329334	785,752	435
ABR4	Bdhn5	pseudomolecule_3	100	544	+/-	19331564	19331021	982,319	544
ABR5	Bdhn4	pseudomolecule_3	100	435	+/-	18704044	18703610	785,752	435
ABR5	Bdhn5	pseudomolecule_3	100	435	+/-	18704044	18703610	785,752	435
ABR6	Bdhn4	pseudomolecule_8	100	435	+/+	29945020	29945454	785.752	435
ABR6	Bdhn5	pseudomolecule_3	100	544	+/-	23110805	23110262	982.319	544
ABR7	Bdhn4	pseudomolecule_3	100	435	+/-	21081433	21080999	785,752	435
ABR7	Bdhn5	pseudomolecule_3	100	544	+/-	21083229	21082686	982,319	544
ABR8	Bdhn4	pseudomolecule_8	100	432	+/+	54933510	54933941	780,342	432
ABR8	Bdhn5	pseudomolecule_8	100	544	+/+	54931705	54932248	982,319	544
ABR9	Bdhn4	pseudomolecule_3	100	432	+/-	21723159	21722728	780,342	432
ABR9	Bdhn5	pseudomolecule_3	100	544	+/-	21724964	21724421	982,319	544
Adi-10	Bdhn4	pseudomolecule_3	100	435	+/-	10831396	10830962	785,752	435
Adi-10	Bdhn5	pseudomolecule_3	100	544	+/-	10833192	10832649	982,319	544
Adi-12	Bdhn4	pseudomolecule_3	96	494	+/-	14671241	14670753	791,162	473
Adi-12	Bdhn5	pseudomolecule_3	95	540	+/-	14669556	14669021	843.46	513
Adi-2	Bdhn4	pseudomolecule_3	100	435	+/-	18208306	18207872	785,752	435
Adi-2	Bdhn5	pseudomolecule_3	100	544	+/-	18210102	18209559	982,319	544
Arn1	Bdhn4	pseudomolecule_3	100	432	+/-	21541017	21540586	780,342	432
Arn1	Bdhn5	pseudomolecule_3	100	544	+/-	21542822	21542279	982,319	544
Bd18-1	Bdhn4	pseudomolecule_4	95	426	+/-	25722056	25721634	672,139	406
Bd18-1	Bdhn5	pseudomolecule_4	100	629	+/-	25723949	25723321	1135.61	629

Bd21-3	Bdhn4	pseudomolecule_4	100	379	+/-	20822271	20821893	684,763	379
Bd21-3	Bdhn5	pseudomolecule_4	100	435	+/-	20820640	20820206	785,752	435
Bd2-3	Bdhn4	pseudomolecule_3	96	494	+/-	14370920	14370432	791,162	473
Bd2-3	Bfhn5	pseudomolecule_3	95	540	+/-	14369235	14368700	843.46	513
Bd29-1	Bdhn4	pseudomolecule_3	100	432	+/-	13503849	13503418	780,342	432
Bd29-1	Bdhn5	pseudomolecule_3	100	544	+/-	13505654	13505111	982,319	544
BdTR11a	Bdhn4	pseudomolecule_3	96	494	+/-	12991466	12990978	791,162	473
BdTR11a	Bdhn5	pseudomolecule_3	95	540	+/-	12989781	12989246	843.46	513
BdTR11g	Bdhn4	pseudomolecule_3	96	494	+/-	17897188	17896700	791,162	473
BdTR11g	Bdhn5	pseudomolecule_3	95	540	+/-	17895503	17894968	843.46	513
BdTR12c	Bdhn4	pseudomolecule_3	100	435	+/-	13785224	13784790	785,752	435
BdTR12c	Bdhn5	pseudomolecule_3	100	437	+/-	13785931	13785495	789,358	437
BdTR13a	Bdhn4	pseudomolecule_3	96	494	+/-	22341144	22340656	791,162	473
BdTR13a	Bdhn5	pseudomolecule_3	95	540	+/-	22339459	22338924	843.46	513
BdTR13c	Bdhn4	pseudomolecule_3	96	494	+/-	13768291	13767803	791,162	473
BdTR13c	Bdhn5	pseudomolecule_3	95	540	+/-	13766606	13766071	843.46	513
BdTR1i	Bdhn4	pseudomolecule_4	96	494	+/-	22473688	22473200	791,162	473
BdTR1i	Bdhn5	pseudomolecule_4	95	540	+/-	22472003	22471468	843.46	513
BdTR2b	Bdhn4	pseudomolecule_4	96	494	+/-	15389921	15389433	791,162	473
BdTR2b	Bdhn5	pseudomolecule_4	95	540	+/-	15388236	15387701	843.46	513
BdTR2g	Bdhn4	pseudomolecule_3	96	494	+/-	13205201	13204713	791,162	473
BdTR2g	Bdhn5	pseudomolecule_3	95	540	+/-	13203516	13202981	843.46	513
BdTR5i	Bdhn4	pseudomolecule_3	96	494	+/-	12824085	12823597	791,162	473
BdTR5i	Bdhn4	pseudomolecule_3	96	494	+/-	12824085	12823597	791,162	473
BdTR5i	Bdhn5	pseudomolecule_3	95	540	+/-	12822400	12821865	843.46	513
BdTR5i	Bdhn5	pseudomolecule_3	95	540	+/-	12822400	12821865	843.46	513
BdTR7a	Bdhn4	pseudomolecule_3	100	432	+/-	19078503	19078072	780,342	432
BdTR7a	Bdhn5	pseudomolecule_3	94	547	+/-	19078491	19077959	825,426	512
BdTR8i	Bdhn4	pseudomolecule_4	96	423	+/-	18828134	18827714	673,943	404

BdTR8i	Bdhn5	pseudomolecule_4	96	423	+/-	18826440	18826021	673,943	404
BdTR9k	Bdhn4	pseudomolecule_3	96	494	+/-	13048836	13048348	791,162	473
BdTR9k	Bdhn5	pseudomolecule_3	95	540	+/-	13047151	13046616	843.46	513
Bis-1	Bdhn4	pseudomolecule_3	96	494	+/-	15209617	15209129	785,752	472
Bis-1	Bdhn5	pseudomolecule_3	95	540	+/-	15207932	15207397	838,049	512
Foz1	Bdhn4a	pseudomolecule_4	100	423	+/-	17367198	17366776	764.111	423
Foz1	Bdhn4b	pseudomolecule_4	100	435	+/-	17380499	17380065	785.752	435
Jer1	Bdhn4	pseudomolecule_3	96	423	+/-	15415773	15415356	691,977	408
Jer1	Bdhn5	pseudomolecule_3	94	547	+/-	15414082	15413547	839,853	515
Kah-1	Bdhn4	pseudomolecule_7	100	435	+/-	73209957	73209523	785.752	435
Kah-1	Bdhn5	pseudomolecule_7	100	544	+/-	66459104	66458561	982.319	544
Kah-5	Bdhn4	pseudomolecule_3	96	494	+/-	17918740	17918252	791,162	473
Kah-5	Bdhn5	pseudomolecule_3	95	540	+/-	17917055	17916520	843.46	513
Koz-1	Bdhn4	pseudomolecule_3	96	494	+/-	18855091	18854603	791,162	473
Koz-1	Bdhn5	pseudomolecule_3	95	540	+/-	18853406	18852871	843.46	513
Luc1	Bdhn4	pseudomolecule_4	100	432	+/-	20923424	20922993	780.342	432
Luc1	Bdhn5	pseudomolecule_4	100	629	+/-	20925314	20924686	1135.61	629
Mig3	Bdhn4	pseudomolecule_3	96	494	+/-	19262494	19262006	791,162	473
Mig3	Bdhn5	pseudomolecule_3	95	540	+/-	19260809	19260274	843.46	513
Mur1	Bdhn4	pseudomolecule_8	97	434	+/+	62525719	62526149	710.01	419
Mur1	Bdhn5	pseudomolecule_8	98	539	+/+	62527300	62527835	913,791	527
Sig2	Bdhn4a	pseudomolecule_3	100	435	+/-	17897303	17896869	785,752	435
Sig2	Bdhn4b	pseudomolecule_3	100	435	+/-	17895616	17895182	780,342	434
Sig2	Bdhn5	pseudomolecule_3	99	934	+/-	17896001	17895069	1645.96	926
Uni2	Bdhn4	pseudomolecule_3	96	494	+/-	15702075	15701587	791,162	473
Uni2	Bdhn5	pseudomolecule_3	95	540	+/-	15700390	15699855	843.46	513

Supplementary Materials S3: ***Brachypodium distachyon*** **drought and temperature stress experiments: grown and treatment conditions**

As described in Des Marais et al. (2017) plants were grown in 600 ml of Profile porous ceramic rooting medium (Profile Products, Buffalo Grove, IL, USA) in Deepot D40H pots (650 ml; Stuewe & Sons, Tangent, OR, USA). The dry weight of each pot was recorded to provide a baseline for the calculation of soil water content (WC). Pots were saturated with a 1:50 dilution of Liquid Grow Plant Food (Dyna-Gro, Richmond, CA, USA) by bottom watering and allowed to drip overnight to field capacity (FC). Two seeds were sown per pot and the pots were weighed to determine FC. The WC of each pot was calculated as (FC) – (dry weight); these WCs provided daily watering targets during the dry-down. Pots were cold stratified at 6°C for 14 d to ensure synchronized germination.

Plants were moved as plots on sequential days from the cold to the glasshouse. All plants germinated within 3 d, after which each pot was thinned to a single plant. During the initial growth period of 21 d, all plants were exposed to ambient glasshouse temperatures with daily highs ranging from 23°C to 28°C and night-time lows from 14°C to 18°C. Natural sunlight was supplemented by artificial lighting to ensure light levels of 400–1000 lmol m_2 photosynthetically active radiation (PAR; mean of 825 lmol m_2) for 10 h d_1 (short-day conditions to prevent rapid flowering). Plants were bottom watered every second day with fresh water and once per week with fertilizer-supplemented water.

Following 21 d of initial growth, each plant received one of four treatments for 10 d in a split-plot design: Cool Wet (CW), Cool Dry (CD), Hot Wet (HW) or Hot Dry (HD), implemented as two plots of each temperature treatment with soil water treatment and genotype fully randomized within each plot. On the 22nd day of growth, each block of plants was transferred to one of four insulated open-top boxes measuring 100 cm wide by 240 cm long by 61 cm high. We placed Redi-Heat seedling mats in the bottom of two of these boxes, which resulted in an increase of c. 10°C above ambient glasshouse temperature; these plants constituted the 'Hot' treatment in which daytime highs were c. 35°C. The two plots of plants without seedling mats constituted the 'Cool' treatment, in which daytime highs were c. 25°C. Within each plot, plants were randomly

assigned to irrigation control (‘Wet’) or restriction (‘Dry’) treatments. Wet plants were watered to FC every second day with fresh water. Dry plants were hand watered daily by pipette such that the soil water was reduced by no more than 5% each day. Harvests began on the 11th day after the beginning of treatment and consisted of 8 d of phenotyping. Pot weights were recorded at harvest and showed that the pots of Dry plants averaged 45.7% soil WC (equivalent to 0.29 g H2Og_1 soil, SE = 0.3%), whereas Wet plants averaged 85.2% soil WC (0.54 g H2Og_1 soil, SE = 0.3%), regardless of temperature treatment. We have shown previously that 40–45% WC in Profile medium corresponds to soil water potentials of c._1.2 MPa and results in a significant reduction in leaf relative water content (RWC) in B. distachyon. Blocks were harvested on sequential days, such that plants had spent the same amount of time from germination through treatment. Control plants did not receive this initial period of stress treatment. On the 22nd day of growth, plants were placed in a 6°C walk-in growth chamber with 270 lmolm_2 PAR for 12 h d_1 to stimulate flowering. After 12 wk of vernalization, plants were returned to the glasshouse under identical conditions as the initial growth period, allowed to acclimate for 5 d, and assigned to temperature and water treatments, as earlier. On the 11th day of treatment, the seedling mats were turned off and water was withheld from all plants until senescence; for most plants, this occurred within 14 d.

Supplementary Materials S4. Values of 12 drought-response phenotypic traits [leaf_rwc (relative water content in leaf); leaf_wc (water content in leaf); lma (leaf mass per área); pro (leaf proline content); abvrgd (above ground biomass); blwgrd (below ground biomass); ttlmass (total mass); rmr (root mass ratio); delta13c (carbon isotope, a proxy for lifetime integrated WUE); leafc (leaf carbon content); leafn (leaf nitrogen content); cn (leaf carbon/nitrogen ratio)] assessed in 32 *Brachypodium distachyon* ecotypes under watered (W) and drought (D) conditions, and hot (H) and cold (C) conditions (Des Marais et al. 2017)

Watered:

	ecotype	leaf_rwc	leaf_wc	lma	pro	abvrgd	blwgrd	ttlmass	rmr	delta13c	leafc	leafn	cn
BA053_HW_ABR2	ABR2	99,153	308,91	28,068	20,0352	98,05	45,15	143,2	31,0668	-31,559	397,29	41,3905	9,6409
BA085_HW_ABR2	ABR2	99,153	308,91	28,068	20,0352	98,05	45,15	143,2	31,0668	-31,559	397,29	41,3905	9,6409
BA403_CW_ABR2	ABR2	99,0252	345,9	26,0716	14,9916	67,6	34,725	102,32	35,8975	-31,0204	391,14	37,0268	10,8998
BA447_CW_ABR2	ABR2	99,0252	345,9	26,0716	14,9916	67,6	34,725	102,32	35,8975	-31,0204	391,14	37,0268	10,8998
BA096_HW_ABR3	ABR3	99,1544	334,96	24,1441	5,3975	138,53	68,175	206,7	33,0524	-30,9512	392,31	37,6329	10,5542
BA413_CW_ABR3	ABR3	99,2622	344,45	22,9499	11,1867	100,68	44,675	145,35	30,4608	-31,7756	388,03	43,64	8,9042
BA419_CW_ABR3	ABR3	99,2622	344,45	22,9499	11,1867	100,68	44,675	145,35	30,4608	-31,7756	388,03	43,64	8,9042
BA040_HW_ABR4	ABR4	98,0294	348,33	27,9507	11,581	63,725	29,85	93,575	31,6254	-31,4833	405,79	42,1945	9,6571
BA043_HW_ABR4	ABR4	98,0294	348,33	27,9507	11,581	63,725	29,85	93,575	31,6254	-31,4833	405,79	42,1945	9,6571
BA477_CW_ABR4	ABR4	100,85	367,39	26,4197	5,9426	63,9332	28,836	92,6436	31,2371	-31,8883	389,73	42,6503	9,1625
BA508_CW_ABR4	ABR4	100,85	367,39	26,4197	5,9426	63,9332	28,836	92,6436	31,2371	-31,8883	389,73	42,6503	9,1625
BA161_HW_ABR5	ABR5	100,35	326,17	26,828	5,4634	103,47	47,075	150,55	31,0022	-32,0899	388,94	40,7959	9,5684
BA479_CW_ABR5	ABR5	100,04	369,42	22,095	8,7886	74,425	26,425	100,85	26,1643	-32,0318	390,46	44,5742	8,8093
BA502_CW_ABR5	ABR5	100,04	369,42	22,095	8,7886	74,425	26,425	100,85	26,1643	-32,0318	390,46	44,5742	8,8093
BA153_HW_ABR6	ABR6	99,7671	341,87	25,7345	12,5268	84,875	34,05	118,93	28,3888	-31,6356	394,87	41,1856	9,6499
BA437_CW_ABR6	ABR6	99,7067	330,68	25,8882	8,9714	77,6	29,4	107	26,7787	-32,0715	386,89	42,6801	9,0705
BA452_CW_ABR6	ABR6	99,7067	330,68	25,8882	8,9714	77,6	29,4	107	26,7787	-32,0715	386,89	42,6801	9,0705
BA008_HW_ABR8	ABR8	98,3205	348,4	27,9114	8,9252	201,2	46,475	247,68	19,0137	-31,1487	384,03	30,6539	12,5536
BA360_CW_ABR8	ABR8	99,4822	365,28	27,9182	7,53	158,13	38,15	196,27	19,5398	-31,7883	382,51	35,7896	10,8115
BA517_CW_ABR8	ABR8	99,4822	365,28	27,9182	7,53	158,13	38,15	196,27	19,5398	-31,7883	382,51	35,7896	10,8115

BA105_HW_Adi10	Adi10	99,5952	368,16	24,5397	11,9688	222,53	73,6296	294,77	24,8531	-31,5077	389,82	31,0325	12,6508
BA428_CW_Adi10	Adi10	99,4321	375,27	24,2527	10,4201	227,2	84,275	311,48	27,1766	-31,6098	387,13	33,1554	11,9649
BA520_CW_Adi10	Adi10	99,4321	375,27	24,2527	10,4201	227,2	84,275	311,48	27,1766	-31,6098	387,13	33,1554	11,9649
BA041_HW_Adi12	Adi12	99,6679	309,95	27,7541	11,0422	176,3	59,45	235,75	24,9133	-31,0063	390,76	36,9663	10,6362
BA044_HW_Adi12	Adi12	99,6679	309,95	27,7541	11,0422	176,3	59,45	235,75	24,9133	-31,0063	390,76	36,9663	10,6362
BA407_CW_Adi12	Adi12	99,1611	335,81	25,6211	9,891	161,4	56,125	217,52	24,964	-31,5626	391,09	40,6269	9,6303
BA423_CW_Adi12	Adi12	99,1611	335,81	25,6211	9,891	161,4	56,125	217,52	24,964	-31,5626	391,09	40,6269	9,6303
BA176_HW_Adi2	Adi2	98,717	301,12	26,5199	7,0812	157,53	52,625	210,15	24,545	-31,4804	390,68	33,8382	11,6383
BA357_CW_Adi2	Adi2	97,504	310,82	25,2906	9,3671	163,35	48,675	212,02	23,2089	-31,165	388,99	35,112	11,2449
BA468_CW_Adi2	Adi2	97,504	310,82	25,2906	9,3671	163,35	48,675	212,02	23,2089	-31,165	388,99	35,112	11,2449
BA354_CW_Bd1.1	Bd1.1	99,4871	340,96	26,0514	8,6774	70,85	28,025	98,875	28,2736	-31,6875	386,51	43,3848	8,9165
BA496_CW_Bd1.1	Bd1.1	99,4871	340,96	26,0514	8,6774	70,85	28,025	98,875	28,2736	-31,6875	386,51	43,3848	8,9165
BA060_HW_Bd18.1	Bd18.1	98,0056	330,2	26,3515	9,0038	166,53	52,55	219,08	23,6441	-31,8404	397,53	36,541	11,1618
BA063_HW_Bd18.1	Bd18.1	98,0056	330,2	26,3515	9,0038	166,53	52,55	219,08	23,6441	-31,8404	397,53	36,541	11,1618
BA375_CW_Bd18.1	Bd18.1	99,3363	359,22	26,0987	6,3918	175,23	75,775	251	29,9302	-31,5193	392,79	39,0647	10,1452
BA446_CW_Bd18.1	Bd18.1	99,3363	359,22	26,0987	6,3918	175,23	75,775	251	29,9302	-31,5193	392,79	39,0647	10,1452
BA086_HW_Bd2.3	Bd2.3	98,93	312,26	27,7804	4,9665	150,93	57,825	208,75	27,4164	-30,7961	384,77	34,4474	11,3351
BA114_HW_Bd2.3	Bd2.3	98,93	312,26	27,7804	4,9665	150,93	57,825	208,75	27,4164	-30,7961	384,77	34,4474	11,3351
BA487_CW_Bd2.3	Bd2.3	100,14	334,18	27,1921	9,3872	134,68	39,55	174,23	23,4222	-31,737	379	38,5101	9,953
BA492_CW_Bd2.3	Bd2.3	100,14	334,18	27,1921	9,3872	134,68	39,55	174,23	23,4222	-31,737	379	38,5101	9,953
BA069_HW_Bd21	Bd21	98,5239	362,35	30,453	7,6058	111,7	36,875	148,58	24,381	-31,4144	395,78	39,9974	9,8958
BA112_HW_Bd21	Bd21	98,5239	362,35	30,453	7,6058	111,7	36,875	148,58	24,381	-31,4144	395,78	39,9974	9,8958
BA386_CW_Bd21	Bd21	98,5417	379,23	25,9302	7,9891	98,175	32,925	131,1	27,3518	-31,4395	381,6	39,2661	9,7509
BA456_CW_Bd21	Bd21	98,5417	379,23	25,9302	7,9891	98,175	32,925	131,1	27,3518	-31,4395	381,6	39,2661	9,7509
BA054_HW_Bd21.3	Bd21.3	100,05	332,87	29,2984	7,2565	163,6	60,5	224,1	26,8571	-32,0124	391,3	35,0021	11,1932
BA171_HW_Bd21.3	Bd21.3	100,05	332,87	29,2984	7,2565	163,6	60,5	224,1	26,8571	-32,0124	391,3	35,0021	11,1932
BA458_CW_Bd21.3	Bd21.3	99,7714	333,71	30,3696	9,4632	172,53	51,775	224,3	21,8706	-31,7525	386,84	35,5076	10,928
BA007_HW_Bd3.1	Bd3.1	98,874	332,27	27,1868	13,1448	212,03	75,775	287,8	26,7347	-31,8078	389,5	36,0408	10,8927

BA012_HW_Bd3.1	Bd3.1	98,874	332,27	27,1868	13,1448	212,03	75,775	287,8	26,7347	-31,8078	389,5	36,0408	10,8927
BA429_CW_Bd3.1	Bd3.1	99,0539	374,33	25,8342	7,8476	182,28	49,1	231,38	21,25	-32,6372	383,12	36,6662	10,4676
BA434_CW_Bd3.1	Bd3.1	99,0539	374,33	25,8342	7,8476	182,28	49,1	231,38	21,25	-32,6372	383,12	36,6662	10,4676
BA005_HW_Bd30.1	Bd30.1	98,4703	359,5	23,884	8,8716	153,97	68,45	222,43	31,0447	-31,6055	375,12	36,2257	10,3997
BA018_HW_Bd30.1	Bd30.1	98,4703	359,5	23,884	8,8716	153,97	68,45	222,43	31,0447	-31,6055	375,12	36,2257	10,3997
BA417_CW_Bd30.1	Bd30.1	99,0166	362,25	24,4767	7,6702	146,13	72,575	218,7	33,5068	-32,6119	377,51	38,9337	9,7014
BA474_CW_Bd30.1	Bd30.1	99,0166	362,25	24,4767	7,6702	146,13	72,575	218,7	33,5068	-32,6119	377,51	38,9337	9,7014
BA384_CW_BdTR10c	BdTR10c	99,8547	346,45	24,4013	9,2635	179,83	78,85	258,67	30,1185	-31,6498	390,6	34,091	11,4878
BA436_CW_BdTR10c	BdTR10c	99,8547	346,45	24,4013	9,2635	179,83	78,85	258,67	30,1185	-31,6498	390,6	34,091	11,4878
BA021_HW_BdTR11g	BdTR11g	99,1281	342,14	28,9609	6,3157	170,22	60,925	231,15	26,2552	-31,9907	390,9	33,0382	12,0813
BA032_HW_BdTR11g	BdTR11g	99,1281	342,14	28,9609	6,3157	170,22	60,925	231,15	26,2552	-31,9907	390,9	33,0382	12,0813
BA406_CW_BdTR11g	BdTR11g	99,2126	355,56	28,0629	6,6034	154,58	58,075	212,65	27,246	-32,1586	391,45	36,1167	10,867
BA493_CW_BdTR11g	BdTR11g	99,2126	355,56	28,0629	6,6034	154,58	58,075	212,65	27,246	-32,1586	391,45	36,1167	10,867
BA042_HW_BdTR11i	BdTR11i	99,1827	340,06	27,1923	7,462	187,9	68,55	256,45	26,5132	-31,4161	392,72	31,2194	12,9344
BA377_CW_BdTR11i	BdTR11i	98,712	364,69	26,6228	7,9247	133,58	51,275	184,85	27,554	-32,162	380,52	38,5626	9,9428
BA526_CW_BdTR11i	BdTR11i	98,712	364,69	26,6228	7,9247	133,58	51,275	184,85	27,554	-32,162	380,52	38,5626	9,9428
BA022_HW_BdTR13a	BdTR13a	98,952	312,12	29,7385	9,0134	132,82	50,4	183,23	27,7306	-32,1181	385,73	36,5552	10,5869
BA174_HW_BdTR13a	BdTR13a	98,952	312,12	29,7385	9,0134	132,82	50,4	183,23	27,7306	-32,1181	385,73	36,5552	10,5869
BA371_CW_BdTR13a	BdTR13a	99,2984	358,92	26,589	6,1106	127,58	47,95	175,53	27,1558	-32,0346	401,71	39,5929	10,1458
BA405_CW_BdTR13a	BdTR13a	99,2984	358,92	26,589	6,1106	127,58	47,95	175,53	27,1558	-32,0346	401,71	39,5929	10,1458
BA070_HW_BdTR1i	BdTR1i	98,5431	310,66	24,9114	10,1893	147,75	54,4	202,15	25,0125	-30,7296	396,33	33,834	11,7319
BA155_HW_BdTR1i	BdTR1i	98,5431	310,66	24,9114	10,1893	147,75	54,4	202,15	25,0125	-30,7296	396,33	33,834	11,7319
BA469_CW_BdTR1i	BdTR1i	98,4507	310,13	26,3632	12,4554	196,35	77,3	273,65	28,3002	-31,4978	383,39	37,4971	10,2676
BA486_CW_BdTR1i	BdTR1i	98,4507	310,13	26,3632	12,4554	196,35	77,3	273,65	28,3002	-31,4978	383,39	37,4971	10,2676
BA378_CW_BdTR2b	BdTR2b	99,1642	334,79	25,5897	9,0804	184,43	74,325	258,75	28,4258	-31,974	396,22	40,6959	9,8107
BA503_CW_BdTR2b	BdTR2b	99,1642	334,79	25,5897	9,0804	184,43	74,325	258,75	28,4258	-31,974	396,22	40,6959	9,8107
BA107_HW_BdTR2g	BdTR2g	98,915	322,42	25,504	9,6107	205,55	75,675	281,23	26,9508	-30,797	390,86	29,8763	13,2668
BA167_HW_BdTR2g	BdTR2g	98,915	322,42	25,504	9,6107	205,55	75,675	281,23	26,9508	-30,797	390,86	29,8763	13,2668

BA445_CW_BdTR2g	BdTR2g	99,141	321,23	26,0647	11,9884	98,575	36,125	134,7	27,1502	-31,3031	394,67	36,8124	10,8874
BA527_CW_BdTR2g	BdTR2g	99,141	321,23	26,0647	11,9884	98,575	36,125	134,7	27,1502	-31,3031	394,67	36,8124	10,8874
BA071_HW_BdTR3c	BdTR3c	99,8607	313,18	26,5389	11,8327	173,03	64,825	237,85	27,34	-32,1045	391,97	32,472	12,0845
BA362_CW_BdTR3c	BdTR3c	99,8703	346,56	24,0444	6,7756	175,03	51,075	226,1	22,5456	-31,5366	393,11	34,2062	11,5274
BA427_CW_BdTR3c	BdTR3c	99,8703	346,56	24,0444	6,7756	175,03	51,075	226,1	22,5456	-31,5366	393,11	34,2062	11,5274
BA160_HW_BdTR5i	BdTR5i	99,7791	320,7	25,8099	6,1914	134,38	55,975	190,35	27,948	-32,0524	385,3	36,6785	10,646
BA372_CW_BdTR5i	BdTR5i	98,1287	326,87	27,244	10,9901	144,7	68,275	212,98	31,5617	-31,8874	392,29	38,8932	10,1175
BA464_CW_BdTR5i	BdTR5i	98,1287	326,87	27,244	10,9901	144,7	68,275	212,98	31,5617	-31,8874	392,29	38,8932	10,1175
BA125_HW_BdTR9k	BdTR9k	99,7491	303,76	28,6036	17,2965	142,33	40,2	182,53	22,0741	-31,5799	391,38	36,5103	10,7677
BA383_CW_BdTR9k	BdTR9k	98,5867	321,85	27,9127	13,0488	156,5	56,85	213,35	26,5012	-31,8205	382,92	39,3275	9,7471
BA515_CW_BdTR9k	BdTR9k	98,5867	321,85	27,9127	13,0488	156,5	56,85	213,35	26,5012	-31,8205	382,92	39,3275	9,7471
BA156_HW_Bis1	Bis1	98,3617	327,15	23,8597	4,6496	117	38,625	155,63	26,3028	-32,2874	390,43	37,3066	10,5286
BA385_CW_Bis1	Bis1	98,4401	329,93	26,5137	9,2391	111,93	43,575	155,5	28,3158	-32,284	392,75	38,7511	10,1582
BA390_CW_Bis1	Bis1	98,4401	329,93	26,5137	9,2391	111,93	43,575	155,5	28,3158	-32,284	392,75	38,7511	10,1582
BA169_HW_Kah1	Kah1	99,8647	295,11	32,0676	7,8515	124,48	39	163,48	23,3447	-31,9871	395,11	38,6626	10,2481
BA356_CW_Kah1	Kah1	98,9119	354,35	26,2315	10,063	142,6	53,875	196,48	27,3753	-31,6866	388,89	40,3501	9,6491
BA420_CW_Kah1	Kah1	98,9119	354,35	26,2315	10,063	142,6	53,875	196,48	27,3753	-31,6866	388,89	40,3501	9,6491
BA047_HW_Kah5	Kah5	98,5779	328,12	27,4317	8,0542	156,68	46,025	202,7	22,9428	-31,0487	391,78	36,9904	10,6068
BA158_HW_Kah5	Kah5	98,5779	328,12	27,4317	8,0542	156,68	46,025	202,7	22,9428	-31,0487	391,78	36,9904	10,6068
BA399_CW_Kah5	Kah5	99,1395	358,79	25,6867	6,6676	166,13	54,575	220,7	24,1437	-31,4011	394,39	37,4447	10,5928
BA482_CW_Kah5	Kah5	99,1395	358,79	25,6867	6,6676	166,13	54,575	220,7	24,1437	-31,4011	394,39	37,4447	10,5928
BA157_HW_Koz1	Koz1	100,34	346,18	24,6393	5,522	111,05	37,025	148,08	24,9715	-31,3085	399,44	37,1463	10,8321
BA501_CW_Koz1	Koz1	99,1951	330,62	27,2562	8,2662	148,05	51,025	199,08	25,7406	-31,9008	396,07	36,3917	10,9175
BA511_CW_Koz1	Koz1	99,1951	330,62	27,2562	8,2662	148,05	51,025	199,08	25,7406	-31,9008	396,07	36,3917	10,9175
BA057_HW_Koz3	Koz3	100,34	346,18	24,6393	5,522	111,05	37,025	148,08	24,9715	-31,3085	399,44	37,1463	10,8321
BA074_HW_Koz3	Koz3	99,2253	381,63	21,3916	3,8404	163,05	57,1	220,15	26,1094	-31,6468	394,63	34,0051	11,7823
BA411_CW_Koz3	Koz3	99,4638	369,31	23,8853	6,3061	197,3	66,325	263,62	24,9763	-32,1301	389,47	34,9537	11,1881
BA484_CW_Koz3	Koz3	99,4638	369,31	23,8853	6,3061	197,3	66,325	263,62	24,9763	-32,1301	389,47	34,9537	11,1881

BA026_HW_Ron2	Ron2	99,478618	362,4171	24,399536	7,125719	137,53043	65,785677	202,9648	31,672994	-31,23899	397,85106	38,796531	10,323291
BA151_HW_Ron2	Ron2	99,478618	362,4171	24,399536	7,125719	137,53043	65,785677	202,9648	31,672994	-31,23899	397,85106	38,796531	10,323291
BA409_CW_Ron2	Ron2	99,540782	374,22254	24,769726	9,395806	125,2623	55,360467	181,36405	30,77086	-31,47118	392,49618	41,641172	9,437255
BA431_CW_Ron2	Ron2	99,540782	374,22254	24,769726	9,395806	125,2623	55,360467	181,36405	30,77086	-31,47118	392,49618	41,641172	9,437255

Drought:

	ecotype	leaf_rwc	leaf_wc	lma	pro	abvrgd	blwgrd	ttlmass	rmr	delta13c	leafc	leafn	cn
BA030_HD_ABR2	ABR2	95,5672	275,21	32,1891	40,0867	88,4	64,925	153,32	41,368	-30,9942	402,01	35,3526	11,415
BA101_HD_ABR2	ABR2	95,5672	275,21	32,1891	40,0867	88,4	64,925	153,32	41,368	-30,9942	402,01	35,3526	11,415
BA366_CD_ABR2	ABR2	96,27	275,61	29,9968	38,994	76,375	44,475	120,85	36,4106	-31,0081	392,34	34,9823	11,23
BA439_CD_ABR2	ABR2	96,27	275,61	29,9968	38,994	76,375	44,475	120,85	36,4106	-31,0081	392,34	34,9823	11,23
BA006_HD_ABR3	ABR3	94,1465	296,47	26,5479	20,5059	101,18	62,475	163,65	38,0076	-30,9528	400,56	32,8802	12,1867
BA103_HD_ABR3	ABR3	94,1465	296,47	26,5479	20,5059	101,18	62,475	163,65	38,0076	-30,9528	400,56	32,8802	12,1867
BA418_CD_ABR3	ABR3	97,6832	354,52	23,1046	16,4585	87,375	52,4	139,78	37,0066	-30,3755	401,97	34,0628	11,8359
BA465_CD_ABR3	ABR3	97,6832	354,52	23,1046	16,4585	87,375	52,4	139,78	37,0066	-30,3755	401,97	34,0628	11,8359
BA038_HD_ABR4	ABR4	95,6684	299,6	30,4712	17,7323	59,125	33,625	92,75	36,419	-30,9893	406,96	34,8378	11,7046
BA170_HD_ABR4	ABR4	95,6684	299,6	30,4712	17,7323	59,125	33,625	92,75	36,419	-30,9893	406,96	34,8378	11,7046
BA368_CD_ABR4	ABR4	97,5747	326,08	27,4177	13,1446	48,725	30,75	79,475	39,3827	-30,98	397,56	36,1803	11,0352
BA521_CD_ABR4	ABR4	97,5747	326,08	27,4177	13,1446	48,725	30,75	79,475	39,3827	-30,98	397,56	36,1803	11,0352
BA024_HD_ABR5	ABR5	93,9699	301,28	28,602	15,5567	88,5	59,475	147,97	39,6646	-31,2665	403,55	37,5765	10,8271
BA104_HD_ABR5	ABR5	93,9699	301,28	28,602	15,5567	88,5	59,475	147,97	39,6646	-31,2665	403,55	37,5765	10,8271
BA454_CD_ABR5	ABR5	97,8872	326,65	25,3111	7,2641	72,297	46,4	118,49	39,1138	-31,0825	386,6	35,9327	10,7333
BA522_CD_ABR5	ABR5	97,8872	326,65	25,3111	7,2641	72,297	46,4	118,49	39,1138	-31,0825	386,6	35,9327	10,7333
BA037_HD_ABR6	ABR6	94,3688	291,38	28,1391	31,3657	81,25	41,45	122,7	33,747	-31,3617	401,51	35,7275	11,2734
BA099_HD_ABR6	ABR6	94,3688	291,38	28,1391	31,3657	81,25	41,45	122,7	33,747	-31,3617	401,51	35,7275	11,2734
BA416_CD_ABR6	ABR6	95,7619	309,23	27,7202	17,3763	64,675	39,5	104,18	37,3018	-31,2286	407,43	33,2924	12,275
BA523_CD_ABR6	ABR6	95,7619	309,23	27,7202	17,3763	64,675	39,5	104,18	37,3018	-31,2286	407,43	33,2924	12,275

BA100_HD_ABR8	ABR8	92,1823	307,46	32,7175	37,0386	129,1	33,25	162,35	20,5232	-31,3316	392,46	32,4176	12,2406
BA143_HD_ABR8	ABR8	92,1823	307,46	32,7175	37,0386	129,1	33,25	162,35	20,5232	-31,3316	392,46	32,4176	12,2406
BA415_CD_ABR8	ABR8	95,8411	348,43	29,1515	13,8568	95,75	33,2	128,95	26,3473	-31,325	393,59	31,1215	12,7443
BA506_CD_ABR8	ABR8	95,8411	348,43	29,1515	13,8568	95,75	33,2	128,95	26,3473	-31,325	393,59	31,1215	12,7443
BA049_HD_Adi10	Adi10	91,5314	335,69	28,3401	83,7468	138,23	60,8	199,02	30,4882	-30,8597	396,7	31,5821	12,5705
BA052_HD_Adi10	Adi10	91,5314	335,69	28,3401	83,7468	138,23	60,8	199,02	30,4882	-30,8597	396,7	31,5821	12,5705
BA478_CD_Adi10	Adi10	93,4741	330,73	29,3769	25,8306	129,5	74,5	204	36,3975	-30,8103	398,07	31,8895	12,499
BA513_CD_Adi10	Adi10	93,4741	330,73	29,3769	25,8306	129,5	74,5	204	36,3975	-30,8103	398,07	31,8895	12,499
BA036_HD_Adi12	Adi12	92,6729	290,48	29,3319	37,4857	158,68	75,7	234,37	32,3829	-30,3287	399,63	32,1182	12,5141
BA140_HD_Adi12	Adi12	92,6729	290,48	29,3319	37,4857	158,68	75,7	234,37	32,3829	-30,3287	399,63	32,1182	12,5141
BA455_CD_Adi12	Adi12	91,5266	291,54	29,3439	22,6328	124,07	65,375	189,45	34,5262	-30,3494	401,56	32,3216	12,4274
BA525_CD_Adi12	Adi12	91,5266	291,54	29,3439	22,6328	124,07	65,375	189,45	34,5262	-30,3494	401,56	32,3216	12,4274
BA050_HD_Adi2	Adi2	94,7074	280,35	29,122	44,1721	106,6	50,6	157,2	32,0808	-30,4593	397,18	30,3205	13,1419
BA094_HD_Adi2	Adi2	94,7074	280,35	29,122	44,1721	106,6	50,6	157,2	32,0808	-30,4593	397,18	30,3205	13,1419
BA500_CD_Adi2	Adi2	95,5478	286,92	28,7405	33,3583	95,05	64,975	160,03	40,5204	-30,9863	394,32	32,8584	12,1132
BA509_CD_Adi2	Adi2	95,5478	286,92	28,7405	33,3583	95,05	64,975	160,03	40,5204	-30,9863	394,32	32,8584	12,1132
BA025_HD_Bd1.1	Bd1.1	96,6513	318,06	27,8895	22,4793	69,95	32,675	102,62	32,6184	-30,7095	396,41	32,6329	12,2739
BA051_HD_Bd1.1	Bd1.1	96,6513	318,06	27,8895	22,4793	69,95	32,675	102,62	32,6184	-30,7095	396,41	32,6329	12,2739
BA442_CD_Bd1.1	Bd1.1	97,5714	338,57	26,4631	9,8706	72,9	34,675	107,58	31,9464	-30,8879	396,28	29,2359	13,6961
BA475_CD_Bd1.1	Bd1.1	97,5714	338,57	26,4631	9,8706	72,9	34,675	107,58	31,9464	-30,8879	396,28	29,2359	13,6961
BA093_HD_Bd18.1	Bd18.1	89,0917	287,91	32,5382	40,6435	132,28	54,4	186,67	29,1314	-30,5819	405,25	33,6488	12,0622
BA453_CD_Bd18.1	Bd18.1	95,8214	323,02	25,8254	12,4704	122,7	66,6	189,51	34,814	-30,5654	410,32	32,0128	12,8275
BA023_HD_Bd2.3	Bd2.3	92,9477	290,3	30,6912	32,8335	115,75	55,225	170,97	32,1404	-30,4738	397	29,9625	13,2597
BA088_HD_Bd2.3	Bd2.3	92,9477	290,3	30,6912	32,8335	115,75	55,225	170,97	32,1404	-30,4738	397	29,9625	13,2597
BA353_CD_Bd2.3	Bd2.3	96,4096	313,1	27,9172	16,2772	103,6	49,8667	153,26	32,3381	-30,9876	391,61	32,8971	11,9916
BA494_CD_Bd2.3	Bd2.3	96,4096	313,1	27,9172	16,2772	103,6	49,8667	153,26	32,3381	-30,9876	391,61	32,8971	11,9916
BA056_HD_Bd21	Bd21	92,1978	329,74	31,3491	19,6817	112,55	45,45	158	28,7482	-30,0903	404,42	32,2814	12,6108
BA163_HD_Bd21	Bd21	92,1978	329,74	31,3491	19,6817	112,55	45,45	158	28,7482	-30,0903	404,42	32,2814	12,6108

BA459_CD_Bd21	Bd21	97,234	367,57	28,5431	14,0697	96,7	52,45	149,15	35,0758	-30,3526	403,03	32,3337	12,5448
BA499_CD_Bd21	Bd21	97,234	367,57	28,5431	14,0697	96,7	52,45	149,15	35,0758	-30,3526	403,03	32,3337	12,5448
BA097_HD_Bd21.3	Bd21.3	86,9049	295,54	35,8918	58,7702	131,23	79,325	210,55	35,7497	-31,192	394,95	34,5557	11,4413
BA111_HD_Bd21.3	Bd21.3	86,9049	295,54	35,8918	58,7702	131,23	79,325	210,55	35,7497	-31,192	394,95	34,5557	11,4413
BA430_CD_Bd21.3	Bd21.3	90,9053	311,26	31,4101	20,4713	136,3	69,35	205,65	33,7667	-30,7154	398,97	33,7282	11,8389
BA512_CD_Bd21.3	Bd21.3	90,9053	311,26	31,4101	20,4713	136,3	69,35	205,65	33,7667	-30,7154	398,97	33,7282	11,8389
BA166_HD_Bd3.1	Bd3.1	90,2132	299,88	29,9041	29,8525	124,08	60,5	184,57	33,1324	-30,6968	402,3	32,1438	12,552
BA398_CD_Bd3.1	Bd3.1	86,8178	321,96	30,5611	44,2785	158,58	66,425	225	29,9478	-30,6395	391,02	28,8347	13,5893
BA422_CD_Bd3.1	Bd3.1	86,8178	321,96	30,5611	44,2785	158,58	66,425	225	29,9478	-30,6395	391,02	28,8347	13,5893
BA079_HD_Bd30.1	Bd30.1	90,7217	272,28	32,0642	49,0163	128,28	60,325	188,6	32,1573	-31,1346	387,86	34,6917	11,3551
BA162_HD_Bd30.1	Bd30.1	90,7217	272,28	32,0642	49,0163	128,28	60,325	188,6	32,1573	-31,1346	387,86	34,6917	11,3551
BA425_CD_Bd30.1	Bd30.1	93,1622	309,6	27,8919	30,0273	88,65	51,325	139,98	36,8631	-31,1783	385,03	35,2732	10,9211
BA481_CD_Bd30.1	Bd30.1	93,1622	309,6	27,8919	30,0273	88,65	51,325	139,98	36,8631	-31,1783	385,03	35,2732	10,9211
BA121_HD_BdTR10c	BdTR10c	93,1128	277,44	29,1343	61,7782	131,55	52,475	184,02	28,5702	-30,7721	403,39	29,1462	14,0575
BA173_HD_BdTR10c	BdTR10c	93,1128	277,44	29,1343	61,7782	131,55	52,475	184,02	28,5702	-30,7721	403,39	29,1462	14,0575
BA421_CD_BdTR10c	BdTR10c	91,2539	307,27	27,9445	62,4874	114,12	57,2	171,33	33,5289	-30,65	402,13	31,7225	12,7066
BA440_CD_BdTR10c	BdTR10c	91,2539	307,27	27,9445	62,4874	114,12	57,2	171,33	33,5289	-30,65	402,13	31,7225	12,7066
BA059_HD_BdTR11g	BdTR11g	89,9507	294,07	31,8587	37,5698	120,03	60,225	180,25	33,1338	-31,1966	402,96	31,3896	12,8764
BA090_HD_BdTR11g	BdTR11g	89,9507	294,07	31,8587	37,5698	120,03	60,225	180,25	33,1338	-31,1966	402,96	31,3896	12,8764
BA397_CD_BdTR11g	BdTR11g	95,3823	312,72	28,7297	15,1499	116,6	57,7	174,3	33,1039	-31,4914	400,42	34,3049	11,7522
BA510_CD_BdTR11g	BdTR11g	95,3823	312,72	28,7297	15,1499	116,6	57,7	174,3	33,1039	-31,4914	400,42	34,3049	11,7522
BA102_HD_BdTR11i	BdTR11i	89,3787	295,88	32,3145	63,7474	126,55	57,3	183,85	31,4774	-31,1197	399,71	30,9229	12,9344
BA128_HD_BdTR11i	BdTR11i	89,3787	295,88	32,3145	63,7474	126,55	57,3	183,85	31,4774	-31,1197	399,71	30,9229	12,9344
BA408_CD_BdTR11i	BdTR11i	94,9355	306,93	30,6664	24,7061	121,07	52,35	173,43	30,7026	-31,104	399,6	33,201	12,1142
BA450_CD_BdTR11i	BdTR11i	94,9355	306,93	30,6664	24,7061	121,07	52,35	173,43	30,7026	-31,104	399,6	33,201	12,1142
BA361_CD_BdtR12c	BdtR12c	95,5726	328,56	25,4422	24,1003	105,52	53,1	158,63	33,6257	-31,4268	396,58	33,3719	11,8987
BA061_HD_BdTR13a	BdTR13a	92,1344	297,05	30,5101	27,5649	104,93	44,75	149,67	30,0713	-31,4933	394,35	35,1597	11,2545
BA394_CD_BdTR13a	BdTR13a	94,9504	299,69	29,8083	16,802	99,975	54	153,98	34,9891	-31,1377	402,13	33,0339	12,2083

BA460_CD_BdTR13a	BdTR13a	94,9504	299,69	29,8083	16,802	99,975	54	153,98	34,9891	-31,1377	402,13	33,0339	12,2083
BA108_HD_BdTR1i	BdTR1i	90,9792	285,17	28,2368	54,0095	128,2	55,85	184,05	29,9311	-30,355	401,84	30,8815	13,0362
BA134_HD_BdTR1i	BdTR1i	90,9792	285,17	28,2368	54,0095	128,2	55,85	184,05	29,9311	-30,355	401,84	30,8815	13,0362
BA457_CD_BdTR1i	BdTR1i	92,2691	298,73	27,4487	30,9165	137,37	79,3	216,68	36,5266	-30,3089	401,02	31,1322	12,9409
BA480_CD_BdTR1i	BdTR1i	92,2691	298,73	27,4487	30,9165	137,37	79,3	216,68	36,5266	-30,3089	401,02	31,1322	12,9409
BA003_HD_BdTR2b	BdTR2b	88,3067	275,41	30,6612	46,3035	142,63	62,4602	205,23	30,4213	-30,4491	401,48	32,0488	12,6043
BA091_HD_BdTR2b	BdTR2b	88,3067	275,41	30,6612	46,3035	142,63	62,4602	205,23	30,4213	-30,4491	401,48	32,0488	12,6043
BA389_CD_BdTR2b	BdTR2b	92,8529	297,14	29,4203	18,8064	128,22	76,15	204,38	36,9819	-30,4727	403,21	33,3256	12,1192
BA472_CD_BdTR2b	BdTR2b	92,8529	297,14	29,4203	18,8064	128,22	76,15	204,38	36,9819	-30,4727	403,21	33,3256	12,1192
BA124_HD_BdTR2g	BdTR2g	83,6304	255,64	30,6727	80,7185	141,48	74,775	216,25	34,4993	-30,5584	388,58	30,9619	12,6063
BA165_HD_BdTR2g	BdTR2g	83,6304	255,64	30,6727	80,7185	141,48	74,775	216,25	34,4993	-30,5584	388,58	30,9619	12,6063
BA364_CD_BdTR2g	BdTR2g	92,8345	295,51	29,707	28,9141	140,7	82,275	222,98	36,2953	-30,2991	406,68	32,9221	12,4293
BA369_CD_BdTR2g	BdTR2g	92,8345	295,51	29,707	28,9141	140,7	82,275	222,98	36,2953	-30,2991	406,68	32,9221	12,4293
BA073_HD_BdTR3c	BdTR3c	87,8504	267,87	30,6517	44,774	103,1	53,15	156,25	34,0107	-30,8192	409,45	28,5468	14,4109
BA172_HD_BdTR3c	BdTR3c	87,8504	267,87	30,6517	44,774	103,1	53,15	156,25	34,0107	-30,8192	409,45	28,5468	14,4109
BA370_CD_BdTR3c	BdTR3c	94,1643	307,36	27,969	14,2319	109,42	63,775	173,2	36,8347	-30,8598	398,76	29,8799	13,3589
BA424_CD_BdTR3c	BdTR3c	94,1643	307,36	27,969	14,2319	109,42	63,775	173,2	36,8347	-30,8598	398,76	29,8799	13,3589
BA065_HD_BdTR5i	BdTR5i	91,8255	282,44	32,7004	53,7029	100,55	65,95	166,5	38,6272	-31,1833	399,66	33,8388	11,906
BA082_HD_BdTR5i	BdTR5i	91,8255	282,44	32,7004	53,7029	100,55	65,95	166,5	38,6272	-31,1833	399,66	33,8388	11,906
BA470_CD_BdTR5i	BdTR5i	94,8835	312	29,218	19,9079	90,6	55,7	146,3	37,1162	-30,8629	401,42	32,836	12,3188
BA473_CD_BdTR5i	BdTR5i	94,8835	312	29,218	19,9079	90,6	55,7	146,3	37,1162	-30,8629	401,42	32,836	12,3188
BA002_HD_BdTR9k	BdTR9k	95,6189	297,03	28,4399	17,6973	143,73	65,025	208,75	32,7704	-30,5734	409,21	32,5471	12,7073
BA033_HD_BdTR9k	BdTR9k	95,6189	297,03	28,4399	17,6973	143,73	65,025	208,75	32,7704	-30,5734	409,21	32,5471	12,7073
BA363_CD_BdTR9k	BdTR9k	94,807	309,37	26,8249	21,5214	122,8	51,775	174,58	29,77	-30,9229	401,68	33,5643	12,0002
BA410_CD_BdTR9k	BdTR9k	94,807	309,37	26,8249	21,5214	122,8	51,775	174,58	29,77	-30,9229	401,68	33,5643	12,0002
BA110_HD_Bis1	Bis1	91,5204	276,88	34,1313	34,8535	117,17	54,9398	171,97	32,1238	-31,0275	399,55	32,1223	12,4379
BA373_CD_Bis1	Bis1	94,8878	312,91	26,9505	14,4399	87,15	41,475	128,63	33,8045	-31,4936	401,23	33,1728	12,1146
BA519_CD_Bis1	Bis1	94,8878	312,91	26,9505	14,4399	87,15	41,475	128,63	33,8045	-31,4936	401,23	33,1728	12,1146

BA046_HD_Kah1	Kah1	93,0147	310,34	29,7299	39,7369	110,85	55,575	166,42	33,7495	-30,7397	402,08	30,9699	13,0816
BA168_HD_Kah1	Kah1	93,0147	310,34	29,7299	39,7369	110,85	55,575	166,42	33,7495	-30,7397	402,08	30,9699	13,0816
BA382_CD_Kah1	Kah1	95,019	320,6	28,6329	22,407	107,22	55,45	162,68	33,7389	-31,0315	403,87	31,9183	12,7028
BA495_CD_Kah1	Kah1	95,019	320,6	28,6329	22,407	107,22	55,45	162,68	33,7389	-31,0315	403,87	31,9183	12,7028
BA048_HD_Kah5	Kah5	91,6097	292,11	29,2052	46,3743	122,93	48,325	171,25	28,4994	-30,3344	404,2	31,2071	12,9653
BA095_HD_Kah5	Kah5	91,6097	292,11	29,2052	46,3743	122,93	48,325	171,25	28,4994	-30,3344	404,2	31,2071	12,9653
BA401_CD_Kah5	Kah5	94,604	331,21	26,7106	20,6909	121,47	48,75	170,23	28,6406	-30,663	402,6	33,2662	12,1173
BA489_CD_Kah5	Kah5	94,604	331,21	26,7106	20,6909	121,47	48,75	170,23	28,6406	-30,663	402,6	33,2662	12,1173
BA089_HD_Koz.3	Koz.3	82,1711	293,25	34,5124	67,5646	103,62	43,625	147,25	29,6711	-30,9751	406,79	29,9398	13,6053
BA395_CD_Koz1	Koz1	95,9543	332,48	26,6403	16,1547	82,2	56,775	138,98	40,0757	-31,2827	404,89	32,4497	12,5778
BA507_CD_Koz1	Koz1	95,9543	332,48	26,6403	16,1547	82,2	56,775	138,98	40,0757	-31,2827	404,89	32,4497	12,5778
BA081_HD_Koz3	Koz3	82,1711	293,25	34,5124	67,5646	103,62	43,625	147,25	29,6711	-30,9751	406,79	29,9398	13,6053
BA388_CD_Koz3	Koz3	88,6229	328	25,7217	20,9671	141,17	80,425	221,6	36,4373	-30,5143	403,35	31,5167	12,7978
BA467_CD_Koz3	Koz3	88,6229	328	25,7217	20,9671	141,17	80,425	221,6	36,4373	-30,5143	403,35	31,5167	12,7978
BA035_HD_Ron2	Ron2	92,82645	327,96356	28,296603	46,865344	97,16903	67,562792	164,90518	38,5043	-30,60872	404,18678	34,5693	12,098827
BA379_D_Ron2	Ron2	95,496932	339,58655	26,694638	22,464309	91,12377	61,060894	153,78871	39,112	-30,60334	404,29112	35,929524	11,507316
BA432_D_Ron2	Ron2	95,496932	339,58655	26,694638	22,464309	91,12377	61,060894	153,78871	39,112	-30,60334	404,29112	35,929524	11,507316
